# Design, synthesis and biological evaluation of novel diosgenin–benzoic acid mustard hybrids with potential anti-proliferative activities in human hepatoma HepG2 cells

**DOI:** 10.1080/14756366.2022.2070161

**Published:** 2022-06-02

**Authors:** Jinling Zhang, Wenbao Wang, Yanzhao Tian, Liwei Ma, Lin Zhou, Hao Sun, Yukun Ma, Huiling Hou, Xiaoli Wang, Jin Ye, Xiaobo Wang

**Affiliations:** a College of Pharmacy, Qiqihar Medical University, Qiqihar, Heilongjiang, P. R. China; b Chinese People’s Liberation Army Logistics Support Force No. 967 Hospital, Dalian, P. R. China

**Keywords:** Diosgenin, benzoic acid mustard, hybrid, cytotoxicity, structure–activity relationships

## Abstract

To discover new lead compounds with anti-tumour activities, in the present study, natural diosgenin was hybridised with the reported benzoic acid mustard pharmacophore. The *in vitro* cytotoxicity of the resulting newly synthesised hybrids (**8**–**10**, **14a**–**14f**, and **15a**–**15f**) was then evaluated in three tumour cells (HepG2, MCF-7, and HeLa) as well as normal GES-1 cells. Among them, **14f** possessed the most potential anti-proliferative activity against HepG2 cells, with an IC_50_ value of 2.26 µM, which was 14.4-fold higher than that of diosgenin (IC_50_ = 32.63 µM). Furthermore, it showed weak cytotoxicity against GES-1 cells (IC_50_ > 100 µM), thus exhibiting good antiproliferative selectivity between normal and tumour cells. Moreover, **14f** could induce G0/G1 arrest and apoptosis of HepG2 cells. From a mechanistic perspective, **14f** regulated cell cycle-related proteins (CDK2, CDK4, CDK6, cyclin D1 and cyclin E1) as well mitochondrial apoptosis pathway-related proteins (Bax, Bcl-2, caspase 9, and caspase 3). These findings suggested that hybrid **14f** serves as a promising anti-hepatoma lead compound that deserves further research.

## Graphical Abstract

**Figure:**
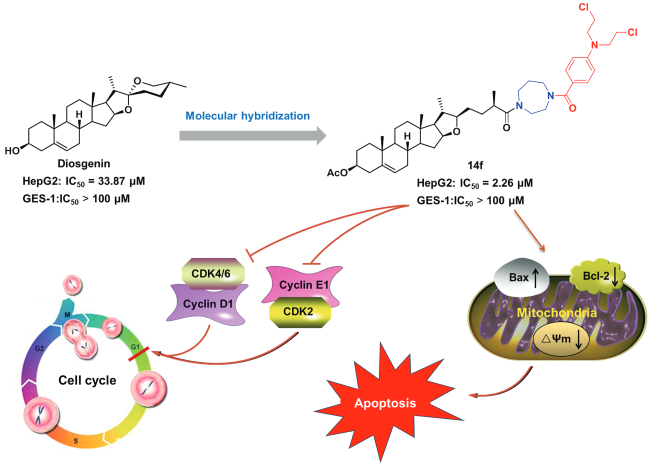

## 1. Introduction

As of September 2019, approximately 84.3% (156 out of 185) of anticancer small molecules approved by the FDA are natural products or their derivatives^1^, which affirms the position of natural products as a focal point for finding structural and medicinal inspiration for drug discovery. Natural steroids and their synthetic derivatives are attracting increasing research interest for their promising anti-tumour activities and potential utilisation in the discovery and design of new anti-tumour agents^2–4^.

**Figure 1.**
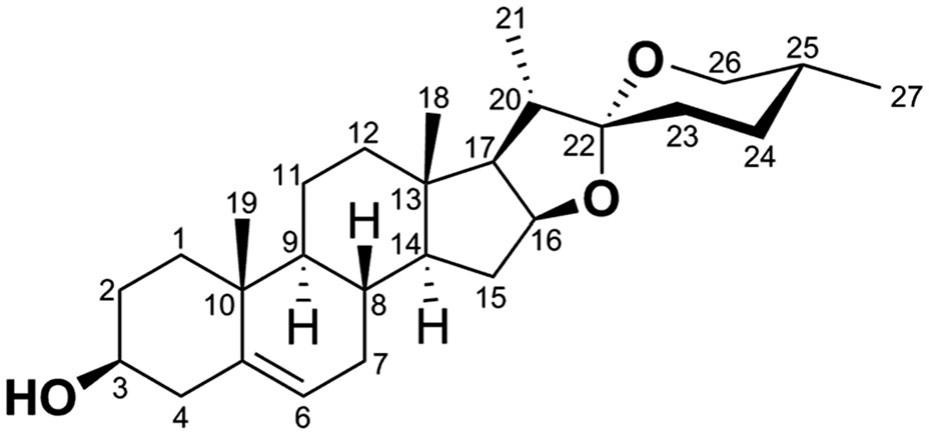
Structure and numbering scheme of diosgenin (DSG, **1**).

Diosgenin (DSG, Figure 1) is a natural steroidal sapogenin isolated from fenugreek seeds and the roots of wild yam (*Dioscorea villosa*)^5^. It possesses many pharmacological functions, such as anti-oxidation, anti-inflammatory, cardiovascular protection, neuroprotection, hypolipidemic, anti-diabetic, and anti-cancer, etc^6^.

In-depth studies on the anti-tumour effects and mechanistic patterns of DSG have revealed that it could regulate multiple genes and several signalling pathways in various types of human cancers. For instance, DSG could arrest the cell cycle at the G2/M phase by regulating the Cdc25C-Cdc2-cyclin B pathway in human breast cancer cells^7^, exert tumour-suppressive function by inhibiting Cdc20 in osteosarcoma cells^8^, induce mitochondria-mediated apoptosis in human cholangiocarcinoma cells and apoptosis via suppression of Skp2 in human breast cancer cells^5^^,^^9^, inhibit the activation of cAMP/PKA/CREB pathway in colorectal cancer cells^6^, and so on. Although DSG possess extensive anti-cancer activity, the application of DSG for cancer therapy was limited by its moderate potency. Therefore, it is important to optimise the scaffold of DSG to obtain promising anti-tumour compounds with improved inhibitory effect.

Nitrogen mustards, which are a type of DNA bifunctional alkylating agents, are developed as clinically useful anti-cancer agents, and include compounds such as chlorambucil, mechlorethamine, melphalan, cyclophosphamide, and estramustine. They exert cytotoxicity by binding to DNA, cross-linking the two chains, and preventing cell replication^10^^,^^11^.

**Figure 2.**
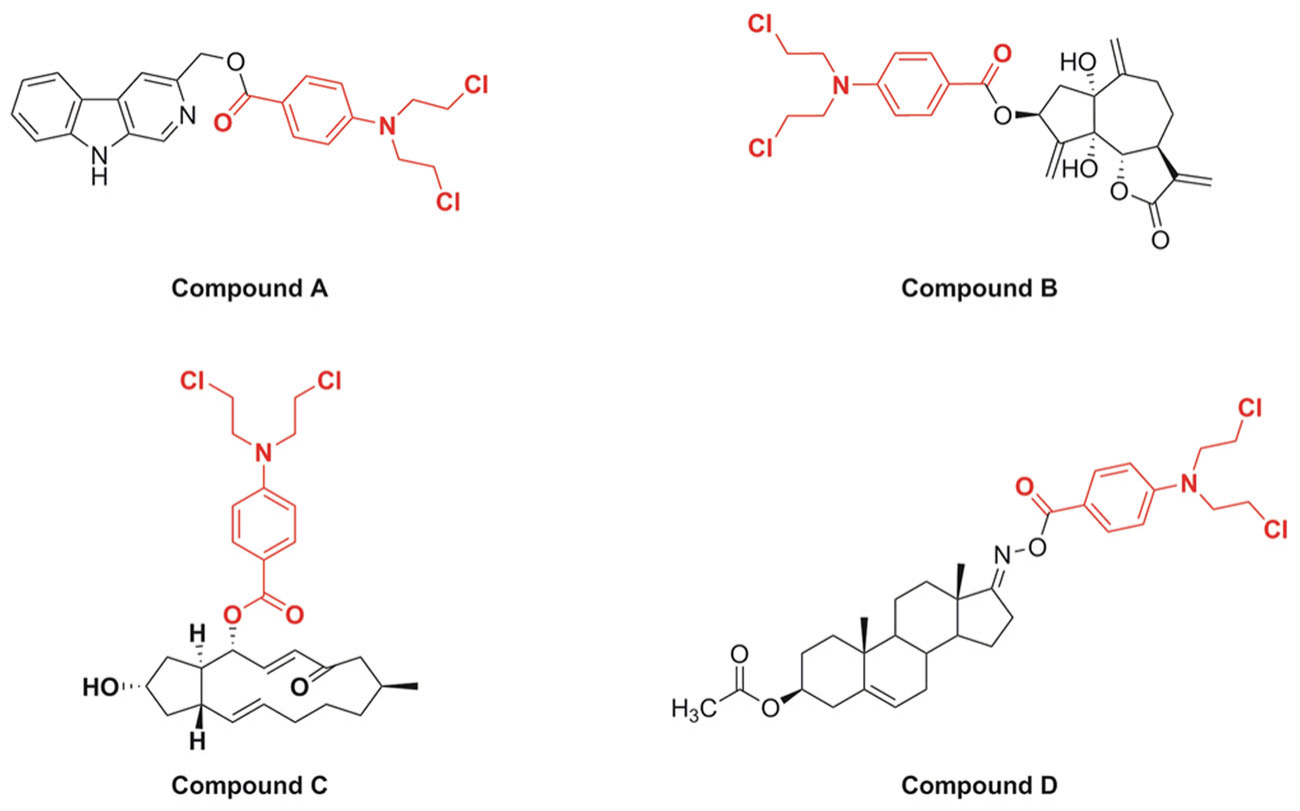
Chemical structures of several examples of natural product and benzoic acid mustard hybrids.

Molecular hybridisation is a widely used strategy in drug discovery, which forms new molecular entities by incorporating two or more bioactive substructures through suitable linkages^12–16^. These hybridised molecules possess improved or new biological properties relative to their individual components^17^. Recently, it has been reported that the hybridisation of natural products with nitrogen mustards provides new strategies for discovering anti-cancer molecules with improved anti-cancer effect, selectivity, and reduced toxicity. For example (Figure 2), a series of *β*-carboline derivatives with nitrogen mustard moieties synthesised by Sun et al. showed potent inhibitory activities in human breast carcinoma cells (MCF-7 and MDA-MB-231); especially, compound **A** containing benzoic acid mustard possessed significant anti-proliferative activity against MCF-7 cells^18^. Compound **B**, which was obtained by introducing a benzoic acid mustard fragment displayed the highest anti-proliferative properties against cervical cancer HeLa cells^19^. In addition, Han et al. synthesised a series of novel conjugates of brefeldin A and nitrogen mustards and found that compound **C** was the most active derivative against Bel-7402 cells^20^. Compound **D**, which was synthesised by conjugating the D-ring-derived androstene oxime with benzoic acid mustard, exhibited the most outstanding effect on inhibiting the growth of ovarian cancer IGROV1 cells^21^. These findings, coupled with the anti-cancer profiles of DSG and benzoic acid mustard, promoted us to further explore the anti-tumour potential of DSG–benzoic acid mustard hybrids.

To the best of our knowledge, there are few reports on the synthesis and biological activity of DSG–benzoic acid mustard derivatives with amide-amide linkages. Therefore, in this study, with the aim of finding new kind of DSG derivatives with improved anti-tumour activity, selectivity, and reduced toxicity, and further explore their structure–activity relationships, fifteen novel DSG–benzoic acid mustard hybrids bearing diversified linkers were designed and synthesised using the molecular hybridisation strategy. The cytotoxic activities of hybrids were evaluated against three cancer cell lines (HepG-2, MCF-7, and HeLa) and normal GES-1 cells. Furthermore, in-depth anti-proliferative mechanisms of the most potent compound, **14f**, including cell cycle progression, expression of cell cycle-related genes and proteins, induction of apoptosis, changes in mitochondrial membrane potential (MMP) and expression of apoptosis-related genes and proteins were explored as well. Taken together, these new results might give researchers some inspiration for structural modification of other natural products, to facilitate discovery of novel anti-tumour agents.

## 2. Experimental

### 2.1. Materials and methods

All reagents were obtained from chemical and biological companies. Anhydrous solvents were dried through routine protocols. DSG derivatives **5**, **6**, and **11** were prepared using our published procedures^22^. The purity of the compounds was measured by HPLC by using a Waters Symmetry C18 (4.6 × 250 mm, 5 µm) column and its peak UV detection was 254 nm. HPLC conditions: methanol/water with 90:10; flow rate 1.0 ml/min. The purity of target compounds was >95% by the analysis of HPLC. Nuclear magnetic resonance (NMR) spectra were recorded using a AVANCE NEO 600 (^1^H, 600 MHz; ^13 ^C, 150 MHz, Bruker) spectrometer. High-resolution mass spectra (HRMS) (ESI) data were measured on TripleTOF^®^ 4600 (AB SCIEX, USA) and Accurate-Mass Q-TOF 6530 (Agilent, USA). The reactions were detected using SGF254 silica gel thin layer chromatography (TLC) (Yantai Huayang New Material Technology Co. Ltd., China). Preparative TLC were performed on layer plates of SGF254 silica gel, 1 mm (Yantai Huayang New Material Technology Co. Ltd.).

#### 2.1.1. (22r,25R)-3β-hydroxy-5-en-furostan-26-ol (7)

A mixture of intermediate **6** (200 mg, 0.436 mmol) and KOH (48.8 mg, 0.872 mmol) in MeOH (8 ml) was stirred at room temperature for 6 h. After confirming the progress of the reaction using TLC, the mixture was poured into 40 ml of H_2_O and filtered. The obtained precipitate was washed with water and dried to yield intermediate **7** (172.4 mg, 95.0%) as a white powder. ^1^H NMR (600 MHz, Chloroform-*d*, TMS) *δ*_H_: 5.33 (d, *J* = 5.0 Hz, 1H, H-6), 4.30 (m, 1H, H-16), 3.53–3.47 (*m*, 2H, H-26, H-3), 3.43 (dd, *J* = 10.6, 6.0 Hz, 1H, H-26), 3.32 (td, *J* = 8.3, 3.8 Hz, 1H, H-22), 1.02 (*s*, 3H, 19-CH_3_), 0.99 (d, *J* = 6.7 Hz, 3H, 21-CH_3_), 0.91 (d, *J* = 6.7 Hz, 3H, 27-CH_3_), 0.80 (*s*, 3H, 18-CH_3_). ^13 ^C NMR (150 MHz, Chloroform-*d*, TMS) *δ*_C_: 140.94 (C-5), 121.56 (C-6), 90.50 (C-22), 83.36 (C-16), 71.83 (C-3), 68.17 (C-26), 65.23 (C-17), 57.11 (C-14), 50.23 (C-9), 42.41 (C-4), 40.84 (C-12), 39.59 (C-13), 38.04 (C-20), 37.39 (C-1), 36.76 (C-10), 35.86 (C-25), 32.37 (C-7), 32.13 (C-15), 31.73 (C-2), 31.73 (C-8), 30.55 (C-24), 30.25 (C-23), 20.83 (C-11), 19.55 (C-19), 19.06 (C-21), 16.77 (C-27), 16.58 (C-18). HRMS (ESI): *m*/*z* calculated for C_27_H_45_O_3_ [M + H]^+^: 417.3363, found: 417.3354.

#### 2.1.2. Synthesis of compounds 8 and 9

Compound **7** (60 mg, 0.145 mmol) was dissolved in dry CH_2_Cl_2_ (6 ml), following which benzoic acid mustard (90.8 mg, 0.348 mmol), EDCI (110.8 mg, 0.58 mmol), and catalytic amount of DMAP were added to it. After 24 h of stirring at room temperature, the obtained solvent was concentrated under vacuum to obtain the crude product, which was purified using preparative TLC (PE:EtOAc = 2:1, *v/v*), to obtain compounds **8** and **9**.

##### 2.1.2.1. (22 R,25R)-3β-hydroxy-5-en-furostan-26–4-(bis(2-chloroethyl)amino)benzoate (8)

White powder, yield 35.6%. HPLC purity 97.44%. ^1^H NMR (600 MHz, Chloroform-*d*, TMS) *δ*_H_: 7.94 (d, *J* = 8.9 Hz, 2H, Ph-H), 6.66 (d, *J* = 8.9 Hz, 2H, Ph-H), 5.34 (d, *J* = 4.8 Hz, 1H, H-6), 4.30 (m, 1H, H-16), 4.17 (dd, *J* = 10.6, 5.6 Hz, 1H, H-26), 4.08 (dd, *J* = 10.6, 6.6 Hz, 1H, H-26), 3.80 (*t*, *J* = 7.0 Hz, 4H, NC*H*_2_CH_2_Cl, ×2), 3.65 (*t*, *J* = 7.0 Hz, 4H, NCH_2_C*H*_2_Cl, ×2), 3.52 (*m*, 1H, H-3), 3.32 (*m*, 1H, H-22), 1.02 (*s*, 3H, 19-CH_3_), 1.01 (d, *J* = 6.6 Hz, 3H, 21-CH_3_), 0.99 (d, *J* = 6.7 Hz, 3H, 27-CH_3_), 0.80 (*s*, 3H, 18-CH_3_). ^13 ^C NMR (150 MHz, Chloroform-*d*, TMS) *δ*_C_: 166.69 (–COO), 149.67 (Ph-C), 140.95 (C-5), 131.91 (Ph-C, ×2), 121.59 (C-6), 119.48 (Ph-C), 110.96 (Ph-C, ×2), 90.36 (C-22), 83.35 (C-16), 71.87 (C-3), 69.31 (C-26), 65.28 (C-17), 57.11 (C-14), 53.46 (NCH_2_*C*H_2_Cl, ×2), 50.24 (C-9), 42.42 (C-4), 40.84 (C-12), 40.23 (N*C*H_2_CH_2_Cl, ×2), 39.59 (C-13), 38.07 (C-20), 37.40 (C-1), 36.77 (C-10), 33.23 (C-24), 32.39 (C-7), 32.16 (C-15), 31.76 (C-8), 31.04 (C-23), 30.73 (C-25), 29.41 (C-2), 20.84 (C-11), 19.57 (C-19), 19.11 (C-21), 17.15 (C-27), 16.58 (C-18). HRMS (ESI): *m*/*z* calculated for C_38_H_56_Cl_2_NO_4_ [M + H]^+^: 660.3581, found: 660.3578.

##### 2.1.2.2. (22 R,25R)-3β-((4-(bis(2-chloroethyl)amino)benzoyl)oxy)-5-en-furostan-26–4-(bis(2-chloroethyl)amino) benzoate (9)

White powder, yield 27.2%. HPLC purity 99.56%. ^1^H NMR (600 MHz, Chloroform-*d*, TMS) *δ*_H_: 7.94 (d, *J* = 8.9 Hz, 2H, Ph-H), 7.93 (d, *J* = 8.8 Hz, 2H, Ph-H), 6.67 (d, *J* = 8.8 Hz, 2H, Ph-H), 6.66 (d, *J* = 8.9 Hz, 2H, Ph-H), 5.40 (d, *J* = 4.8 Hz, 1H, H-6), 4.80 (*m*, 1H, H-3), 4.31 (*m*, 1H, H-16), 4.17 (dd, *J* = 10.9, 5.7 Hz, 1H, H-26), 4.08 (dd, *J* = 10.9, 6.8 Hz, 1H, H-26), 3.80 (*t*, *J* = 7.0 Hz, 8H, NC*H*_2_CH_2_Cl, ×4), 3.65 (*t*, *J* = 7.0 Hz, 8H, NCH_2_C*H*_2_Cl, ×4), 3.33 (*m*, 1H, H-22）, 1.08 (*s*, 3H, 19-CH_3_), 1.02 (d, *J* = 6.8 Hz, 3H, 21-CH_3_), 1.00 (d, *J* = 6.7 Hz, 3H, 27-CH_3_), 0.81 (*s*, 3H, 18-CH_3_). ^13 ^C NMR (150 MHz, Chloroform-*d*, TMS) *δ*_C_: 166.68 (-COO), 166.02 (-COO), 149.67 (Ph-C), 149.62 (Ph-C), 140.04 (C-5), 131.90 (Ph-C, ×4), 122.48 (C-6), 119.75 (Ph-C), 119.48 (Ph-C), 110.96 (Ph-C, ×2), 110.93 (Ph-C, ×2), 90.36 (C-22), 83.35 (C-16), 73.98 (C-3), 69.31 (C-26), 65.27 (C-17), 57.06 (C-14), 53.46 (NCH_2_*C*H_2_Cl, ×4), 50.16 (C-9), 40.84 (C-12), 40.25 (N*C*H_2_CH_2_Cl, ×4), 39.56 (C-13), 38.45 (C-4), 38.09 (C-20), 37.22 (C-1), 36.93 (C-10), 33.23 (C-24), 32.39 (C-7), 32.18 (C-15), 31.75 (C-8), 31.05 (C-23), 30.74 (C-25), 27.35 (C-2), 20.81 (C-11), 19.57 (C-19), 19.10 (C-21), 17.15 (C-27), 16.59 (C-18). HRMS (ESI): *m*/*z* calculated for C_49_H_67_Cl_4_N_2_O_5_Na [M + Na]^+^: 925.3618, found: 925.3587.

#### 2.1.3. Synthesis of (22 R,25R)-3β-acetoxy-5-en-furostan-26–4-(bis(2-chloroethyl)amino)benzoate (10)

Compound **6** (40 mg, 0.087 mmol) was used to synthesise compound **10**, according to the method described to prepare compounds **8** and **9** from **7**.

White powder, yield 50.8%. HPLC purity 97.32%. ^1^H NMR (600 MHz, Chloroform-*d*, TMS) *δ*_H_: 7.94 (d, *J* = 9.0 Hz, 2H, Ph-H), 6.66 (d, *J* = 9.0 Hz, 2H, Ph-H), 5.37 (d, *J* = 4.9 Hz, 1H, H-6), 4.59 (*m*, 1H, H-3), 4.30 (*m*, 1H, H-16), 4.17 (dd, *J* = 10.8, 5.7 Hz, 1H, H-26), 4.08 (dd, *J* = 10.8, 6.7 Hz, 1H, H-26), 3.80 (*t*, *J* = 7.1 Hz, 4H, NC*H*_2_CH_2_Cl, ×2), 3.65 (*t*, *J* = 7.1 Hz, 4H, NCH_2_C*H*_2_Cl, ×2), 3.32 (*m*, 1H, H-22), 2.03 (*s*, 3H, Ac-CH_3_), 1.03 (*s*, 3H, 19-CH_3_), 1.01 (d, *J* = 6.7 Hz, 3H, 21-CH_3_), 0.99 (d, *J* = 6.7 Hz, 3H, 27-CH_3_), 0.80 (*s*, 3H, 18-CH_3_). ^13 ^C NMR (150 MHz, Chloroform-*d*, TMS) *δ*_C_: 170.69 (Ac-COO), 166.69 (-COO), 149.67 (Ph-C), 139.84 (C-5), 131.92 (Ph-C, ×2), 122.53 (C-6), 119.48 (Ph-C), 110.96 (Ph-C, ×2), 90.36 (C-22), 83.35 (C-16), 74.03 (C-3), 69.31 (C-26), 65.27 (C-17), 57.04 (C-14), 53.46 (NCH_2_*C*H_2_Cl, ×2), 50.14 (C-9), 40.83 (C-12), 40.24 (N*C*H_2_CH_2_Cl, ×2), 39.54 (C-13), 38.24 (C-4), 38.09 (C-20), 37.14 (C-1), 36.86 (C-10), 33.24 (C-24), 32.38 (C-7), 32.14 (C-15), 31.72 (C-8), 31.05 (C-23), 30.74 (C-25), 27.89 (C-2), 21.58 (Ac-CH_3_), 20.79 (C-11), 19.49 (C-19), 19.10 (C-21), 17.15 (C-27), 16.58 (C-18). HRMS (ESI): *m*/*z* calculated for C_40_H_58_Cl_2_NO_5_ [M + H]^+^: 724.3506, found: 724.3474.

#### 2.1.4. General procedure for synthesising 12a–12f

To a stirred solution of intermediate **11** (80 mg, 0.169 mmol) and *N*-Boc-protected amines (0.338 mmol) in CH_2_Cl_2_ (5 ml), 2-(1H-benzotriazole-1-yl)-1,1,3,3-tetramethylaminium tetrafluoroborate (TBTU; 108.8 mg, 0.338 mmol) and *N*, *N*-diisopropylethylamine (DIPEA; 43.7 mg, 0.338 mmol) were added, following which the solution was stirred for 8 h at room temperature. After the reaction was completed, the solvent was evaporated at reduced pressure to get a residue, which was purified using preparative TLC (PE:EtOAc = 1:1, *v/v*), to obtain compounds **12a**–**12f**.

##### 2.1.4.1. (22 R,25R)-(boc-(2-aminoethyl))-3β-acetoxy-5-en-furostan 26-amide (12a)

White oil, yield 84.6%. ^1^H NMR (600 MHz, Chloroform-*d*, TMS) *δ*_H_: 6.46 (br s, 1H, NH), 5.36 (d, *J* = 4.6 Hz, 1H, H-6), 4.59 (*m*, 1H, H-3), 4.31 (*m*, 1H, H-16), 3.34 (*m*, 2H, NHCH_2_), 3.30 (*m*, 1H, H-22), 3.25 (*m*, 2H, NHCH_2_), 2.34 (*m*, 1H, H-25), 2.03 (*s*, 3H, Ac-CH_3_), 1.43 (*s*, 9H, Boc-CH_3_, ×3), 1.12 (d, *J* = 6.6 Hz, 3H, 21-CH_3_), 1.03 (*s*, 3H, 19-CH_3_), 0.98 (d, *J* = 6.7 Hz, 3H, 27-CH_3_), 0.79 (*s*, 3H, 18-CH_3_). ^13 ^C NMR (150 MHz, Chloroform-*d*, TMS) *δ*_C_: 177.49 (-CONH), 170.69 (Ac-COO), 156.86 (Boc-COO), 139.87 (C-5), 122.44 (C-6), 90.82 (C-22), 83.54 (C-16), 79.70 (Boc-*quart*.-C), 74.02 (C-3), 64.85 (C-17), 57.03 (C-14), 50.12 (C-9), 40.91 (NHCH_2_), 40.82 (C-12), 40.34 (NHCH_2_), 39.52 (C-13), 38.36 (C-4), 38.23 (C-20), 37.13 (C-1), 36.86 (C-10), 32.59 (C-24), 32.47 (C-7), 32.12 (C-15), 31.70 (C-8), 31.00 (C-23), 28.54 (Boc-CH_3_, ×3), 27.88 (C-2), 21.57 (Ac-CH_3_), 20.77 (C-11), 19.48 (C-19), 18.86 (C-21), 18.06 (C-27), 16.62 (C-18). HRMS (ESI): *m*/*z* calculated for C_36_H_58_N_2_O_6_Na [M + Na]^+^: 637.4193, found: 637.4162.

##### 2.1.4.2. (22 R,25R)-(boc-(2-aminopropyl))-3β-acetoxy-5-en-furostan 26-amide (12 b)

Yellowish oil, yield 80.9%. ^1^H NMR (600 MHz, Chloroform-*d*, TMS) *δ*_H_: 5.36 (d, *J* = 4.7 Hz, 1H, H-6), 4.59 (*m*, 1H, H-3), 4.30 (*m*, 1H, H-16), 3.30 (*m*, 4H, NHCH_2_, ×2), 3.13 (*m*, 1H, H-22), 2.36 (*m*, 1H, H-25), 2.02 (*s*, 3H, Ac-CH_3_), 1.43 (*s*, 9H, Boc-CH_3_, ×3), 1.13 (d, *J* = 6.6 Hz, 3H, 21-CH_3_), 1.03 (*s*, 3H, 19-CH_3_), 0.97 (d, *J* = 6.8 Hz, 3H, 27-CH_3_), 0.78 (*s*, 3H, 18-CH_3_). ^13 ^C NMR (150 MHz, Chloroform-*d*, TMS) *δ*_C_: 177.31 (-CONH), 170.67 (Ac-COO), 156.59 (Boc-COO), 139.87 (C-5), 122.43 (C-6), 90.85 (C-22), 83.52 (C-16), 79.33 (Boc-*quart*.-C), 74.00 (C-3), 64.81 (C-17), 57.01 (C-14), 50.11 (C-9), 40.99 (NHCH_2_), 40.90 (NHCH_2_), 40.80 (C-12), 39.50 (C-13), 38.37 (C-4), 38.22 (C-20), 37.12 (C-1), 36.84 (C-10), 35.97 (C-25), 32.63 (C-24), 32.47 (C-7), 32.11 (C-15), 31.71 (C-8), 31.12 (C-23), 28.56 (Boc-CH_3_, ×3), 28.52 (NHCH_2_CH_2_), 27.87 (C-2), 21.56 (Ac-CH_3_), 20.76 (C-11), 19.48 (C-19), 18.87 (C-21), 18.23 (C-27), 16.55 (C-18). HRMS (ESI): *m*/*z* calculated for C_37_H_60_N_2_O_6_Na [M + Na]^+^: 651.4349, found: 651.4307.

##### 2.1.4.3. (22 R,25R)-(3-(boc-amino)azetidinyl))-3β-acetoxy-5-en-furostan 26-amide (12c)

White oil, yield 83.5%. ^1^H NMR (600 MHz, Chloroform-*d*, TMS) *δ*_H_: 5.37 (d, *J* = 4.4 Hz, 1H, H-6), 4.60 (*m*, 1H, H-3), 4.46 (*m*, 1H, NHCH), 4.37 (*m*, 1H, NC*H*H), 4.28 (*m*, 2H, H-16, NC*H*H), 3.94 (*m*, 1H, NCH*H*), 3.78 (*m*, 1H, NCH*H*), 3.28 (*m*, 1H, H-22), 2.34 (*m*, 1H, H-25), 2.03 (*s*, 3H, Ac-CH_3_), 1.44 (*s*, 9H, Boc-CH_3_, ×3), 1.07 (d, *J* = 6.6 Hz, 3H, 21-CH_3_), 1.03 (*s*, 3H, 19-CH_3_), 0.98 (d, *J* = 6.5 Hz, 3H, 27-CH_3_), 0.79 (*s*, 3H, 18-CH_3_). ^13 ^C NMR (150 MHz, Chloroform-*d*, TMS) *δ*_C_: 176.61 (-CON), 170.68 (Ac-COO), 155.00 (Boc-COO), 139.85 (C-5), 122.49 (C-6), 90.27 (C-22), 83.37 (C-16), 80.42 (Boc-*quart*.-C), 74.03 (C-3), 65.21 (C-17), 57.02 (C-14), 55.07 (NCH_2_, ×2), 50.13 (C-9), 40.81 (C-12), 40.39 (NHCH), 39.52 (C-13), 38.92 (C-4), 38.25 (C-20), 37.13 (C-1), 36.85 (C-10), 35.63 (C-25), 32.42 (C-7), 32.13 (C-15), 31.72 (C-8), 31.48 (C-24), 31.34 (C-23), 28.46 (Boc-CH_3_, ×3), 27.88 (C-2), 21.57 (Ac-CH_3_), 20.78 (C-11), 19.48 (C-19), 19.07 (C-21), 17.60 (C-27), 16.62 (C-18). HRMS (ESI): *m*/*z* calculated for C_37_H_59_N_2_O_6_ [M + H]^+^: 627.4368, found: 627.4350.

##### 2.1.4.4. (22 R,25R)-((S)-3-(boc-amino)pyrrolidyl)-3β-acetoxy-5-en-furostan 26-amide (12d)

White oil, yield 79.8%. ^1^H NMR (600 MHz, Chloroform-*d*, TMS) *δ*_H_: 5.37 (d, *J* = 4.5 Hz, 1H, H-6), 4.60 (*m*, 1H, H-3), 4.58 (*m*, 1H, NHCH), 4.29 (*m*, 1H, H-16), 3.75 (*m*, 1H, NC*H*H), 3.60 (*m*, 1H, NCH*H*), 3.50 (*m*, 1H, NCH*H*), 3.33 (*m*, 1H, NC*H*H), 3.29 (*m*, 1H, H-22), 2.58 (*m*, 1H, H-25), 2.03 (*s*, 3H, Ac-CH_3_), 1.44 (*s*, 9H, Boc-CH_3_, ×3), 1.09 (d, *J* = 6.5 Hz, 3H, 21-CH_3_), 1.03 (*s*, 3H, 19-CH_3_), 0.97 (d, *J* = 6.3 Hz, 3H, 27-CH_3_), 0.78 (*s*, 3H, 18-CH_3_). ^13 ^C NMR (150 MHz, Chloroform-*d*, TMS) *δ*_C_: 175.63 (-CON), 170.68 (Ac-COO), 155.34 (Boc-COO), 139.82 (C-5), 122.53 (C-6), 90.38 (C-22), 83.34 (C-16), 80.18 (Boc-*quart*.-C), 74.03 (C-3), 65.22 (C-17), 57.02 (C-14), 52.54 (NCH_2_), 50.96 (NHCH), 50.13 (C-9), 43.74 (NCH_2_), 40.80 (C-12), 39.52 (C-13), 38.26 (C-4), 38.23 (C-20), 38.19 (NCH_2_CH_2_), 37.74 (C-25), 37.13 (C-1), 36.85 (C-10), 32.39 (C-7), 32.13 (C-15), 31.71 (C-8), 31.52 (C-24), 31.48 (C-23), 28.49 (Boc-CH_3_, ×3), 27.88 (C-2), 21.57 (Ac-CH_3_), 20.78 (C-11), 19.48 (C-19), 19.08 (C-21), 17.79 (C-27), 16.57 (C-18). HRMS (ESI): *m*/*z* calculated for C_38_H_60_N_2_O_6_Na [M + Na]^+^: 663.4349, found: 663.4314.

##### 2.1.4.5. (22 R,25R)-(boc-1-piperazinyl)-3β-acetoxy-5-en-furostan 26-amide (12e)

White powder, yield 86.0%. ^1^H NMR (600 MHz, Chloroform-*d*, TMS) *δ*_H_: 5.36 (d, *J* = 4.0 Hz, 1H, H-6), 4.59 (*m*, 1H, H-3), 4.27 (*m*, 1H, H-16), 3.46–3.63 (*m*, 4H, NCH_2_, ×2), 3.57–3.43 (*m*, 4H, NCH_2_, ×2), 3.29 (*m*, 1H, H-22), 2.75 (*m*, 1H, H-25), 2.02 (*s*, 3H, Ac-CH_3_), 1.46 (*s*, 9H, Boc-CH_3_, ×3), 1.09 (d, *J* = 6.8 Hz, 3H, 21-CH_3_), 1.02 (*s*, 3H, 19-CH_3_), 0.97 (d, *J* = 6.6 Hz, 3H, 27-CH_3_), 0.77 (*s*, 3H, 18-CH_3_). ^13 ^C NMR (150 MHz, Chloroform-*d*, TMS) *δ*_C_: 175.23 (-CON), 170.65 (Ac-COO), 154.75 (Boc-COO), 139.83 (C-5), 122.46 (C-6), 90.15 (C-22), 83.34 (C-16), 80.34 (Boc-*quart*.-C), 74.00 (C-3), 65.17 (C-17), 56.99 (C-14), 50.11 (C-9), 45.44 (NCH_2_, ×2), 41.66 (NCH_2_, ×2), 40.78 (C-12), 39.49 (C-13), 38.23 (C-4), 38.21 (C-20), 37.11 (C-1), 36.83 (C-10), 35.39 (C-25), 32.38 (C-7), 32.11 (C-15), 31.70 (C-8), 31.53 (C-24), 31.18 (C-23), 28.51 (Boc-CH_3_, ×3), 27.87 (C-2), 21.56 (Ac-CH_3_), 20.76 (C-11), 19.46 (C-19), 19.01 (C-21), 17.96 (C-27), 16.57 (C-18). HRMS (ESI): *m*/*z* calculated for C_38_H_60_N_2_O_6_Na [M + Na]^+^: 663.4349, found: 663.4312.

##### 2.1.4.6. (22 R,25R)-(boc-1-homopiperazinyl)-3β-acetoxy-5-en-furostan 26-amide (12f)

White oil, yield 84.9%. ^1^H NMR (600 MHz, Chloroform-*d*, TMS) *δ*_H_: 5.36 (d, *J* = 4.5 Hz, 1H, H-6), 4.59 (*m*, 1H, H-3), 4.28 (*m*, 1H, H-16), 3.61 (*m*, 2H, NCH_2_), 3.53 (*m*, 2H, NCH_2_), 3.46 (*m*, 2H, NHCH_2_), 3.37 (*m*, 2H, NHCH_2_), 3.29 (*t*, *J* = 7.5 Hz, 1H, H-22), 2.70 (*m*, 1H, H-25), 2.02 (*s*, 3H, Ac-CH_3_), 1.45 (*s*, 9H, Boc-CH_3_, ×3), 1.10 (d, *J* = 6.8 Hz, 3H, 21-CH_3_), 1.02 (*s*, 3H, 19-CH_3_), 0.97 (d, *J* = 6.6 Hz, 3H, 27-CH_3_), 0.77 (*s*, 3H, 18-CH_3_). ^13 ^C NMR (150 MHz, Chloroform-*d*, TMS) *δ*_C_: 176.49 (-CON), 170.65 (Ac-COO), 155.21 (Boc-COO), 139.83 (C-5), 122.48 (C-6), 90.31 (C-22), 83.34 (C-16), 79.90 (Boc-quart.-C), 74.01 (C-3), 65.20 (C-17), 56.99 (C-14), 53.95 (NCH_2_), 50.12 (C-9), 49.16 (NCH_2_), 48.86 (NCH_2_), 48.68 (NCH_2_), 40.77 (C-12), 39.50 (C-13), 38.24 (C-4), 38.22 (C-20), 37.12 (C-1), 36.84 (C-10), 35.83 (C-25), 32.35 (C-7), 32.12 (C-15), 31.98 (C-8), 31.70 (C-24), 31.43 (C-23), 29.41 (NCH_2_*C*H_2_), 28.55 (Boc-CH_3_, ×3), 27.87 (C-2), 21.56 (Ac-CH_3_), 20.76 (C-11), 19.47 (C-19), 19.04 (C-21), 18.29 (C-27), 16.55 (C-18). HRMS (ESI): *m*/*z* calculated for C_39_H_63_N_2_O_6_ [M + H]^+^: 655.4681, found: 655.4672.

#### 2.1.5. General procedure for synthesising 13a–13f

To a solution of compounds **12a**–**12f** (0.12 mmol) in dry CH_2_Cl_2_ (6 ml), CF_3_COOH (0.5 ml) was added. The resulting mixture was stirred for 4 h at room temperature. The reaction solution was adjusted to a pH in the range of 7–9 using an aqueous saturated solution of NaHCO_3_ (40 ml), extracted with CH_2_Cl_2_ (5 ml × 3), following which the organic layer was separated, combined, and washed thrice with water (25 ml) and an aqueous saturated solution of NaCl (25 ml), dried over anhydrous Na_2_SO_4_, filtered, and evaporated under reduced pressure, to yield compounds **13a**–**13f**.

##### 2.1.5.1. (22 R,25R)-(2-aminoethyl)-3β-acetoxy-5-en-furostan 26-amide (13a)

White powder, yield 72.4%. ^1^H NMR (600 MHz, Chloroform-*d*, TMS) *δ*_H_: 7.59 (s, 1H, NH), 5.36 (br s, 1H, H-6), 4.59 (*m*, 1H, H-3), 4.29 (*m*, 1H, H-16), 3.60 (br s, 1H, NHC*H*H), 3.42 (br s, 1H, NHCH*H*), 3.30 (*m*, 1H, H-22), 3.12 (br s, 2H, NH_2_C*H*_2_), 2.35 (*m*, 1H, H-25), 2.03 (*s*, 3H, Ac-CH_3_), 1.10 (d, *J* = 5.8 Hz, 3H, 21-CH_3_), 1.03 (*s*, 3H, 19-CH_3_), 0.97 (d, *J* = 6.0 Hz, 3H, 27-CH_3_), 0.78 (*s*, 3H, 18-CH_3_). ^13 ^C NMR (150 MHz, Chloroform-*d*, TMS) *δ*_C_: 178.56 (-CONH), 170.66 (Ac-COO), 139.84 (C-5), 122.45 (C-6), 90.22 (C-22), 83.37 (C-16), 73.99 (C-3), 65.10 (C-17), 57.04 (C-14), 50.13 (C-9), 40.82 (C-12), 40.07 (NHCH_2_), 39.53 (C-13), 38.25 (C-4), 38.04 (C-20), 37.45 (NH_2_CH_2_), 37.14 (C-1), 36.84 (C-10), 36.10 (C-25), 32.40 (C-7), 32.13 (C-15), 31.70 (C-8), 31.56 (C-23), 31.19 (C-24), 27.89 (C-2), 21.57 (Ac-CH_3_), 20.80 (C-11), 19.51 (C-19), 19.04 (C-21), 17.93 (C-27), 16.67 (C-18). HRMS (ESI): *m*/*z* calculated for C_31_H_51_N_2_O_4_ [M + H]^+^: 515.3843, found: 515.3809.

##### 2.1.5.2. (22 R,25R)-(2-aminopropyl)-3β-acetoxy-5-en-furostan 26-amide (13 b)

White powder, yield 73.0%. ^1^H NMR (600 MHz, Chloroform-*d*, TMS) *δ*_H_: 5.36 (br s, 1H, H-6), 4.59 (*m*, 1H, H-3), 4.30 (*m*, 1H, H-16), 3.40 (*m*, 2H, NHCH_2_), 3.31 (*m*, 1H, H-22), 2.38 (*m*, 1H, H-25), 2.02 (*s*, 3H, Ac-CH_3_), 1.10 (d, *J* = 5.8 Hz, 3H, 21-CH_3_), 1.03 (*s*, 3H, 19-CH_3_), 0.97 (d, *J* = 5.8 Hz, 3H, 27-CH_3_), 0.77 (*s*, 3H, 18-CH_3_). ^13 ^C NMR (150 MHz, Chloroform-*d*, TMS) *δ*_C_: 178.13 (-CONH), 170.67 (Ac-COO), 139.88 (C-5), 122.37 (C-6), 90.82 (C-22), 83.54 (C-16), 73.98 (C-3), 64.77 (C-17), 57.01 (C-14), 50.10 (C-9), 40.82 (C-12), 40.61 (NHCH_2_), 39.50 (NH_2_CH_2_), 39.47 (C-13), 38.32 (C-4), 38.22 (C-20), 37.11 (C-1), 36.83 (C-10), 36.24 (C-25), 32.51 (C-7), 32.44 (NHCH_2_*C*H_2_), 32.11 (C-15), 31.70 (C-8), 31.05 (C-24), 30.98 (C-23), 27.87 (C-2), 21.56 (Ac-CH_3_), 20.75 (C-11), 19.49 (C-19), 18.87 (C-21), 18.18 (C-27), 16.61 (C-18). HRMS (ESI): *m*/*z* calculated for C_32_H_53_N_2_O_4_ [M + H]^+^: 529.4000, found: 529.3973.

##### 2.1.5.3. (22 R,25R)-(3-aminoazetidinyl)-3β-acetoxy-5-en-furostan 26-amide (13c)

White powder, yield 70.2%. ^1^H NMR (600 MHz, Chloroform-*d*, TMS) *δ*_H_: 5.37 (d, *J* = 4.5 Hz, 1H, H-6), 4.59 (*m*, 1H, H-3), 4.37 (*m*, 1H, NC*H*H), 4.29 (*m*, 1H, H-16), 4.24 (*m*, 1H, NC*H*H), 3.90 (*m*, 2H, NCH*H*, NH_2_CH), 3.74 (*m*, 1H, NCH*H*), 3.28 (*m*, 1H, H-22), 2.35 (*m*, 1H, H-25), 2.03 (*s*, 3H, Ac-CH_3_), 1.07 (d, *J* = 6.0 Hz, 3H, 21-CH_3_), 1.03 (*s*, 3H, 19-CH_3_), 0.98 (d, *J* = 6.2 Hz, 3H, 27-CH_3_), 0.79 (*s*, 3H, 18-CH_3_). ^13 ^C NMR (150 MHz, Chloroform-*d*, TMS) *δ*_C_: 176.94 (-CON), 170.68 (Ac-COO), 139.86 (C-5), 122.49 (C-6), 90.27 (C-22), 83.35 (C-16), 74.02 (C-3), 65.24 (C-17), 57.36 (NCH_2_, ×2), 57.01 (C-14), 50.13 (C-9), 42.20 (NH_2_CH_2_), 40.81 (C-12), 39.52 (C-13), 38.24 (C-4), 38.18 (C-20), 37.14 (C-1), 36.86 (C-10), 35.67 (C-25), 32.41 (C-7), 32.13 (C-15), 31.73 (C-8), 31.42 (C-24), 31.17 (C-23), 27.89 (C-2), 21.58 (Ac-CH_3_), 20.78 (C-11), 19.49 (C-19), 19.09 (C-21), 17.59 (C-27), 16.60 (C-18). HRMS (ESI): *m*/*z* calculated for C_32_H_51_N_2_O_4_ [M + H]^+^: 527.3843, found: 527.3842.

##### 2.1.5.4. (22 R,25R)-((S)-3-aminopyrrolidyl)-3β-acetoxy-5-en-furostan 26-amide (13d)

White powder, yield 73.7%. ^1^H NMR (600 MHz, Chloroform-*d*, TMS) *δ*_H_: 5.37 (d, *J* = 4.7 Hz, 1H, H-6), 4.59 (*m*, 1H, H-3), 4.27 (*m*, 1H, H-16), 3.76 (*m*, 1H, NC*H*H), 3.68 (*m*, 2H, NH_2_CH, NCH*H*), 3.50 (*m*, 1H, NCH*H*), 3.32 (*m*, 1H, NC*H*H), 3.29 (*m*, 1H, H-22), 2.57 (*m*, 1H, H-25), 2.02 (*s*, 3H, Ac-CH_3_), 1.12 (d, *J* = 7.1 Hz, 3H, 21-CH_3_), 1.03 (*s*, 3H, 19-CH_3_), 0.98 (d, *J* = 6.8 Hz, 3H, 27-CH_3_), 0.78 (*s*, 3H, 18-CH_3_). ^13 ^C NMR (150 MHz, Chloroform-*d*, TMS) *δ*_C_: 175.73 (-CON), 170.68 (Ac-COO), 139.86 (C-5), 122.49 (C-6), 90.36 (C-22), 83.33 (C-16), 74.02 (C-3), 65.21 (C-17), 57.01 (C-14), 51.75 (NCH_2_), 50.12 (C-9), 49.79 (NH_2_CH), 44.80 (NCH_2_), 40.80 (C-12), 39.52 (C-13), 38.23 (C-4), 38.23 (C-20), 38.16 (NCH_2_*C*H_2_), 37.78 (C-25), 37.13 (C-1), 36.85 (C-10), 32.40 (C-7), 32.13 (C-15), 31.71 (C-8), 31.46 (C-24), 31.40 (C-23), 27.88 (C-2), 21.57 (Ac-CH_3_), 20.78 (C-11), 19.48 (C-19), 19.09 (C-21), 17.73 (C-27), 16.57 (C-18). HRMS (ESI): *m*/*z* calculated for C_33_H_53_N_2_O_4_ [M + H]^+^: 541.4000, found: 541.3998.

##### 2.1.5.5. (22 R,25R)-(piperazinyl)-3β-acetoxy-5-en-furostan 26-amide (13e)

White powder, yield 76.1%. ^1^H NMR (600 MHz, Chloroform-*d*, TMS) *δ*_H_: 5.35 (d, *J* = 4.7 Hz, 1H, H-6), 4.58 (*m*, 1H, H-3), 4.27 (*m*, 1H, H-16), 3.50–3.64 (*m*, 4H, NCH_2_, ×2), 3.29 (*m*, 1H, H-22), 2.87–2.84 (*m*, 4H, NHCH_2_, ×2), 2.73 (*m*, 1H, H-25), 2.01 (*s*, 3H, Ac-CH_3_), 1.08 (d, *J* = 6.9 Hz, 3H, 21-CH_3_), 1.02 (*s*, 3H, 19-CH_3_), 0.97 (d, *J* = 6.7 Hz, 3H, 27-CH_3_), 0.77 (*s*, 3H, 18-CH_3_). ^13 ^C NMR (150 MHz, Chloroform-*d*, TMS) *δ*_C_: 175.06 (-CON), 170.69 (Ac-COO), 139.82 (C-5), 122.46 (C-6), 90.19 (C-22), 83.31 (C-16), 74.02 (C-3), 65.15 (C-17), 56.98 (C-14), 50.10 (C-9), 45.96 (NCH_2_, ×2), 42.56 (NHCH_2_, ×2), 40.77 (C-12), 39.48 (C-13), 38.20 (C-4), 38.16 (C-20), 37.09 (C-1), 36.82 (C-10), 35.23 (C-25), 32.36 (C-7), 32.09 (C-15), 31.68 (C-8), 31.47 (C-24), 31.22 (C-23), 27.85 (C-2), 21.55 (Ac-CH_3_), 20.74 (C-11), 19.45 (C-19), 19.00 (C-21), 17.95 (C-27), 16.55 (C-18). HRMS (ESI): *m*/*z* calculated for C_33_H_53_N_2_O_4_ [M + H]^+^: 541.4000, found: 541.3962.

##### 2.1.5.6. (22 R,25R)-(1-homopiperazinyl)-3β-acetoxy-5-en-furostan 26-amide (13f)

White powder, yield 74.4%. ^1^H NMR (600 MHz, Chloroform-*d*, TMS) *δ*_H_: 5.36 (d, *J* = 4.0 Hz, 1H, H-6), 4.59 (*m*, 1H, H-3), 4.28 (*m*, 1H, H-16), 3.65 (*m*, 2H, NCH_2_), 3.58 (*m*, 2H, NCH_2_), 3.29 (*t*, *J* = 8.0 Hz, 1H, H-22), 2.97 (*m*, 2H, NHCH_2_), 2.93 (*m*, 1H, NHC*H*H), 2.87 (*m*, 1H, NHCH*H*), 2.73 (*m*, 1H, H-25), 2.02 (*s*, 3H, Ac-CH_3_), 1.11 (d, *J* = 6.8 Hz, 3H, 21-CH_3_), 1.02 (*s*, 3H, 19-CH_3_), 0.97 (d, *J* = 6.6 Hz, 3H, 27-CH_3_), 0.77 (*s*, 3H, 18-CH_3_). ^13 ^C NMR (150 MHz, Chloroform-*d*, TMS) *δ*_C_: 176.37 (-CON), 170.65 (Ac-COO), 139.84 (C-5), 122.47 (C-6), 90.34 (C-22), 83.33 (C-16), 74.01 (C-3), 65.20 (C-17), 57.00 (C-14), 50.60 (NCH_2_), 50.12 (C-9), 49.14 (NHCH_2_), 48.13 (NCH_2_), 46.81 (NHCH_2_), 40.77 (C-12), 39.50 (C-13), 38.22 (C-4), 38.2 (C-20), 37.11 (C-1), 36.84 (C-10), 35.81 (C-25), 32.35 (C-7), 32.11 (C-15), 31.85 (C-8), 31.70 (C-24), 31.46 (C-23), 29.29 (NCH_2_*C*H_2_), 27.87 (C-2), 21.55 (Ac-CH_3_), 20.76 (C-11), 19.46 (C-19), 19.05 (C-21), 18.41 (C-27), 16.55 (C-18). HRMS (ESI): *m*/*z* calculated for C_34_H_55_N_2_O_4_ [M + H]^+^: 555.4156, found: 555.4181.

#### 2.1.6. General procedure for synthesising 14a–14f

To a solution of **13a**–**13f** (0.07 mmol) in dry CH_2_Cl_2_ (5 ml), benzoic acid mustard (21 mg, 0.08 mmol), 1-ethyl-3–(3-dimethylaminopropyl) carbodiimide hydrochloride (EDCI; 20 mg, 0.105 mmol), and 4-dimethylaminopyridine (DMAP; catalytic amount) were added. After being stirred for 24 h at room temperature, the mixture was concentrated *in vacuo*. The product was purified using preparative TLC, with a petroleum ether/ethyl acetate (1:2, *v/v*) system, to achieve the compounds **14a**–**14f**.

##### 2.1.6.1. (22 R,25R)-(2-aminoethyl-4-(bis(2-chloroethyl)amino)benzamido)-3β-acetoxy-5-en-furostan-26-amide (14a)

White powder, yield 88.0%. HPLC purity 95.16%. ^1^H NMR (600 MHz, Chloroform-*d*, TMS) *δ*_H_: 7.75 (d, *J* = 8.8 Hz, 2H, Ph-H), 7.52 (*s*, 1H, NH), 6.95 (*s*, 1H, NH), 6.65 (d, *J* = 8.8 Hz, 2H, Ph-H), 5.35 (d, *J* = 3.8 Hz, 1H, H-6), 4.58 (*m*, 1H, H-3), 4.27 (*m*, 1H, H-16), 3.76 (*t*, *J* = 6.8 Hz, 4H, NC*H*_2_CH_2_Cl, ×2), 3.63 (*t*, *J* = 6.8 Hz, 4H, NCH_2_C*H*_2_Cl, ×2), 3.52 (*m*, 2H, NHCH_2_), 3.49 (*m*, 2H, NHCH_2_), 3.24 (*t*, *J* = 7.9 Hz, 1H, H-22), 2.40 (*m*, 1H, H-25), 2.02 (*s*, 3H, Ac-CH_3_), 1.10 (d, *J* = 6.6 Hz, 3H, 21-CH_3_), 1.02 (*s*, 3H, 19-CH_3_), 0.88 (d, *J* = 6.6 Hz, 3H, 27-CH_3_), 0.74 (*s*, 3H, 18-CH_3_). ^13 ^C NMR (150 MHz, Chloroform-*d*, TMS) *δ*_C_: 178.96 (-CONH), 170.65 (Ac-COO), 167.60 (-CONH), 148.62 (Ph-C), 139.84 (C-5), 129.26 (Ph-C, ×2), 122.69 (C-6), 122.35 (Ph-C), 111.17 (Ph-C, ×2), 90.89 (C-22), 83.52 (C-16), 73.97 (C-3), 64.71 (C-17), 56.97 (C-14), 53.45 (NCH_2_*C*H_2_Cl, ×2), 50.06 (C-9), 40.76 (C-12), 40.64 (NHCH_2_), 40.30 (N*C*H_2_CH_2_Cl, ×2), 39.42 (C-13), 39.34 (NHCH_2_), 38.31 (C-4), 38.19 (C-20), 37.08 (C-1), 36.80 (C-10), 36.17 (C-25), 32.48 (C-7), 32.06 (C-15), 31.69 (C-8), 31.66 (C-23), 30.87 (C-24), 27.84 (C-2), 21.53 (Ac-CH_3_), 20.71 (C-11), 19.45 (C-19), 18.73 (C-21), 18.04 (C-27), 16.54 (C-18). HRMS (ESI): *m*/*z* calculated for C_42_H_62_Cl_2_N_3_O_5_ [M + H]^+^: 758.4061, found: 758.4044.

##### 2.1.6.2. (22 R,25R)-(3-aminopropyl-4-(bis(2-chloroethyl)amino)benzamido)-3β-acetoxy-5-en- furostan-26-amide (14 b)

White oil, yield 85.6%. HPLC purity 98.17%. ^1^H NMR (600 MHz, Chloroform-*d*, TMS) *δ*_H_: 7.84 (d, *J* = 8.7 Hz, 2H, Ph-H), 6.69 (d, *J* = 8.7 Hz, 2H, Ph-H), 5.36 (d, *J* = 4.8 Hz, 1H, H-6), 4.59 (*m*, 1H, H-3), 4.32 (*m*, 1H, H-16), 3.78 (*t*, *J* = 7.0 Hz, 4H, NC*H*_2_CH_2_Cl, ×2), 3.64 (*t*, *J* = 7.0 Hz, 4H, NCH_2_C*H*_2_Cl, ×2), 3.45 (*m*, 2H, NHCH_2_), 3.34 (*m*, 2H, NHCH_2_), 3.32 (*m*, 1H, H-22), 2.45 (*m*, 1H, H-25), 2.03 (*s*, 3H, Ac-CH_3_), 1.15 (d, *J* = 6.9 Hz, 3H, 21-CH_3_), 1.03 (*s*, 3H, 19-CH_3_), 0.97 (d, *J* = 6.8 Hz, 3H, 27-CH_3_), 0.77 (*s*, 3H, 18-CH_3_). ^13 ^C NMR (150 MHz, Chloroform-*d*, TMS) *δ*_C_: 178.04 (-CONH), 170.68 (Ac-COO), 167.21 (-CONH), 148.61 (Ph-C), 139.90 (C-5), 129.35 (Ph-C, ×2), 123.06 (Ph-C), 122.37 (C-6), 111.29 (Ph-C, ×2), 91.00 (C-22), 83.61 (C-16), 73.99 (C-3), 64.71 (C-17), 57.01 (C-14), 53.51 (NCH_2_*C*H_2_Cl, ×2), 50.10 (C-9), 40.87 (NHCH_2_), 40.82 (C-12), 40.31 (N*C*H_2_CH_2_Cl, ×2), 39.47 (C-13), 38.45 (C-4), 38.22 (C-20), 37.12 (C-1), 36.84 (C-10), 35.80 (NHCH_2_), 35.79 (C-25), 32.84 (NHCH_2_*C*H_2_), 32.52 (C-7), 32.10 (C-15), 31.71 (C-8), 31.02 (C-24), 30.15 (C-23), 27.86 (C-2), 21.56 (Ac-CH_3_), 20.75 (C-11), 19.49 (C-19), 18.83 (C-21), 18.28 (C-27), 16.57 (C-18). HRMS (ESI): *m*/*z* calculated for C_43_H_64_Cl_2_N_3_O_5_ [M + H]^+^: 772.4218, found: 772.4202.

##### 2.1.6.3. (22 R,25R)-(3-aminoazetidinyl-4-(bis(2-chloroethyl)amino)benzamido)-3β-acetoxy-5- en-furostan-26-amide (14c)

White oil, yield 81.4%. HPLC purity 95.12%. ^1^H NMR (600 MHz, Chloroform-*d*, TMS) *δ*_H_: 7.77 (d, *J* = 8.5 Hz, 2H, Ph-H), 6.67 (d, *J* = 8.5 Hz, 2H, Ph-H), 5.35 (d, *J* = 4.0 Hz, 1H, H-6), 4.83 (*m*, 1H, NHCH), 4.58 (*m*, 1H, H-3), 4.54 (*m*, 1H, NC*H*H), 4.37 (*m*, 1H, NC*H*H), 4.28 (*m*, 1H, H-16), 4.05 (*m*, 1H, NCH*H*), 3.95 (*m*, 1H, NCH*H*), 3.79 (*t*, *J* = 6.9 Hz, 4H, NC*H*_2_CH_2_Cl, ×2), 3.65 (*t*, *J* = 6.9 Hz, 4H, NCH_2_C*H*_2_Cl, ×2), 3.28 (*m*, 1H, H-22), 2.37 (*m*, 1H, H-25), 2.02 (*s*, 3H, Ac-CH_3_), 1.09 (d, *J* = 6.9 Hz, 3H, 21-CH_3_), 1.04 (*s*, 3H, 19-CH_3_), 0.98 (d, *J* = 6.9 Hz, 3H, 27-CH_3_), 0.76 (*s*, 3H, 18-CH_3_). ^13 ^C NMR (150 MHz, Chloroform-*d*, TMS) *δ*_C_: 176.91 (-CON), 170.68 (Ac-COO), 167.02 (-CONH), 149.06 (Ph-C), 139.82 (C-5), 129.42 (Ph-C, ×2), 122.50 (C-6), 122.13 (Ph-C), 111.23 (Ph-C, ×2), 90.24 (C-22), 83.41 (C-16), 74.04 (C-3), 65.17 (C-17), 57.02 (C-14), 54.40 (NCH_2_, ×2), 53.42 (NCH_2_*C*H_2_Cl, ×2), 50.13 (C-9), 40.80 (NHCH), 40.76 (C-12), 40.26 (N*C*H_2_CH_2_Cl, ×2), 39.52 (C-13), 38.23 (C-4), 38.18 (C-20), 37.12 (C-1), 36.85 (C-10), 35.55 (C-25), 32.40 (C-7), 32.12 (C-15), 31.72 (C-8), 31.46 (C-24), 31.11 (C-23), 27.88 (C-2), 21.56 (Ac-CH_3_), 20.78 (C-11), 19.47 (C-19), 19.05 (C-21), 17.53 (C-27), 16.61 (C-18). HRMS (ESI): *m*/*z* calculated for C_43_H_62_Cl_2_N_3_O_5_ [M + H]^+^: 770.4061, found: 770.4100.

##### 2.1.6.4. (22 R,25R)-((S)-3-aminopyrrolidyl-4-(bis(2-chloroethyl)amino)benzamido)-3β-acetoxy-5-en-furostan-26-amide (14d)

White powder, yield 82.4%. HPLC purity 95.21%. ^1^H NMR (600 MHz, Chloroform-*d*, TMS) *δ*_H_: 7.69 (d, *J* = 7.8 Hz, 2H, Ph-H), 6.65 (d, *J* = 7.8 Hz, 2H, Ph-H), 6.30 (*s*, 1H, NH), 5.34 (d, *J* = 4.3 Hz, 1H, H-6), 4.68 (*m*, 1H, NH_2_CH), 4.58 (*m*, 1H, H-3), 4.28 (*m*, 1H, H-16), 3.94 (*m*, 1H, NC*H*H), 3.77 (*t*, *J* = 6.9 Hz, 4H, NC*H*_2_CH_2_Cl, ×2), 3.70 (*m*, 1H, NC*H*H), 3.63 (*t*, *J* = 6.9 Hz, 4H, NCH_2_C*H*_2_Cl, ×2), 3.57 (*m*, 1H, NCH*H*), 3.47 (*m*, 1H, NCH*H*), 3.28 (*m*, 1H, H-22), 2.58 (*m*, 1H, H-25), 2.02 (*s*, 3H, Ac-CH_3_), 1.10 (d, *J* = 7.0 Hz, 3H, 21-CH_3_), 1.02 (*s*, 3H, 19-CH_3_), 0.97 (d, *J* = 6.8 Hz, 3H, 27-CH_3_), 0.78 (*s*, 3H, 18-CH_3_). ^13 ^C NMR (150 MHz, Chloroform-*d*, TMS) *δ*_C_: 175.83 (-CON), 170.66 (Ac-COO), 167.07 (-CONH), 148.93 (Ph-C), 139.79 (C-5), 129.22 (Ph-C, ×2), 124.32 (Ph-C), 122.47 (C-6), 111.23 (Ph-C, ×2), 90.34 (C-22), 83.34 (C-16), 74.00 (C-3), 65.14 (C-17), 56.97 (C-14), 53.40 (NCH_2_*C*H_2_Cl, ×2), 52.63 (NCH_2_), 50.22 (NH_2_CH), 50.09 (C-9), 44.10 (NCH_2_), 40.76 (C-12), 40.26 (N*C*H_2_CH_2_Cl, ×2), 39.49 (C-13), 38.24 (C-4), 38.20 (C-20), 38.10 (NCH_2_*C*H_2_), 37.62 (C-25), 37.09 (C-1), 36.81 (C-10), 32.34 (C-7), 32.09 (C-15), 31.66 (C-8), 31.57 (C-24), 31.40 (C-23), 27.85 (C-2), 21.55 (Ac-CH_3_), 20.74 (C-11), 19.45 (C-19), 19.03 (C-21), 17.75 (C-27), 16.58 (C-18). HRMS (ESI): *m*/*z* calculated for C_44_H_64_Cl_2_N_3_O_5_ [M + H]^+^: 784.4218, found: 784.4247.

##### 2.1.6.5. (22 R,25R)-(1-piperazinyl-4-(bis(2-chloroethyl)amino)benzamido)-3β-acetoxy-5-en-furostan-26-amide (14e)

White oil, yield 90.6%. HPLC purity 99.55%. ^1^H NMR (600 MHz, Chloroform-*d*, TMS) *δ*_H_: 7.37 (d, *J* = 8.3 Hz, 2H, Ph-H), 6.68 (d, *J* = 8.3 Hz, 2H, Ph-H), 5.36 (d, *J* = 4.3 Hz, 1H, H-6), 4.59 (*m*, 1H, H-3), 4.27 (*m*, 1H, H-16), 3.77 (*t*, *J* = 7.0 Hz, 4H, NC*H*_2_CH_2_Cl, ×2), 3.66 (*m*, 4H, NCH_2_, ×2), 3.64 (*t*, *J* = 7.0 Hz, 4H, NCH_2_C*H*_2_Cl, ×2), 3.57 (*m*, 4H, NCH_2_, ×2), 3.30 (*m*, 1H, H-22), 2.78 (*m*, 1H, H-25), 2.02 (*s*, 3H, Ac-CH_3_), 1.11 (d, *J* = 6.7 Hz, 3H, 21-CH_3_), 1.03 (*s*, 3H, 19-CH_3_), 0.98 (d, *J* = 6.9 Hz, 3H, 27-CH_3_), 0.78 (*s*, 3H, 18-CH_3_). ^13 ^C NMR (150 MHz, Chloroform-*d*, TMS) *δ*_C_: 175.32 (-CON), 170.87 (-CON), 170.65 (Ac-COO), 147.74 (Ph-C), 139.86 (C-5), 129.96 (Ph-C, ×2), 123.65 (Ph-C), 122.45 (C-6), 111.38 (Ph-C, ×2), 90.14 (C-22), 83.36 (C-16), 73.99 (C-3), 65.14 (C-17), 56.99 (C-14), 53.47 (NCH_2_*C*H_2_Cl, ×2), 50.11 (C-9), 45.61 (NCH_2_, ×2), 42.01 (NCH_2_, ×2), 40.79 (C-12), 40.28 (N*C*H_2_CH_2_Cl, ×2), 39.48 (C-13), 38.25 (C-4), 38.22 (C-20), 37.11 (C-1), 36.83 (C-10), 35.37 (C-25), 32.4 (C-7), 32.12 (C-15), 31.71 (C-8), 31.54 (C-24), 31.14 (C-23), 27.86 (C-2), 21.56 (Ac-CH_3_), 20.76 (C-11), 19.48 (C-19), 19.00 (C-21), 17.93 (C-27), 16.59 (C-18). HRMS (ESI): *m*/*z* calculated for C_44_H_64_Cl_2_N_3_O_5_ [M + H]^+^: 784.4218, found: 784.4257.

##### 2.1.6.6. (22 R,25R)-(4–(1-homopiperazinyl)-4-(bis(2-chloroethyl)amino)benzamido)- 3β-acetoxy-5-en-furostan-26-amide (14f)

White oil, yield 89.1%. HPLC purity 95.91%. ^1^H NMR (600 MHz, Chloroform-*d*, TMS) *δ*_H_: 7.33 (br s, 2H, Ph-H), 6.69 (br s, 2H, Ph-H), 5.37 (d, *J* = 3.8 Hz, 1H, H-6), 4.59 (*m*, 1H, H-3), 4.28 (*m*, 1H, H-16), 3.76 (*t*, *J* = 6.8 Hz, 4H, NC*H*_2_CH_2_Cl, ×2), 3.65 (*t*, *J* = 6.8 Hz, 4H, NCH_2_C*H*_2_Cl, ×2), 3.63–3.52 (*m*, 8H, NCH_2_, ×4), 3.29 (*t*, *J* = 8.0 Hz, 1H, H-22), 2.74 (*m*, 1H, H-25), 2.03 (*s*, 3H, Ac-CH_3_), 1.11 (d, *J* = 6.8 Hz, 3H, 21-CH_3_), 1.03 (*s*, 3H, 19-CH_3_), 0.97 (d, *J* = 6.6 Hz, 3H, 27-CH_3_), 0.78 (*s*, 3H, 18-CH_3_). ^13 ^C NMR (150 MHz, Chloroform-*d*, TMS) *δ*_C_: 176.48 (-CON), 172.03 (-CON), 170.68 (Ac-COO), 147.18 (Ph-C), 139.87 (C-5), 129.66 (Ph-C, ×2), 122.48 (C-6), 121.17 (Ph-C), 111.74 (Ph-C, ×2), 90.29 (C-22), 83.37 (C-16), 74.02 (C-3), 65.18 (C-17), 57.00 (C-14), 53.73 (NCH_2_*C*H_2_Cl, ×2), 53.68 (NCH_2_), 50.12 (C-9), 49.32 (NCH_2_), 48.47 (NCH_2_), 48.15 (NCH_2_), 40.79 (C-12), 40.31 (N*C*H_2_CH_2_Cl, ×2), 39.51 (C-13), 38.26 (C-4), 38.23 (C-20), 37.13 (C-1), 36.85 (C-10), 35.89 (C-25), 32.37 (C-7), 32.14 (C-15), 31.90 (C-8), 31.72 (C-24), 31.45 (C-23), 29.20 (NCH_2_*C*H_2_), 27.88 (C-2), 21.57 (Ac-CH_3_), 20.77 (C-11), 19.49 (C-19), 19.07 (C-21), 18.76 (C-27), 16.59 (C-18). HRMS (ESI): *m*/*z* calculated for C_45_H_66_Cl_2_N_3_O_5_ [M + H]^+^: 798.4374, found: 798.4410.

#### 2.1.7. General procedure for synthesising 15a–15f

A solution of compounds **14a**–**14f** in aqueous NaOH (4 N) in methanol:tetrahydrofuran (1:1.5, *v/v*) was stirred for 6 h at room temperature. After concentration *in vacuo*, 30 ml of water was added to it and it was extracted with CH_2_Cl_2_ (6 ml × 3). The CH_2_Cl_2_ extract was washed with aqueous saturated NaCl (25 ml) and dried over anhydrous Na_2_SO_4_, following which the solids were removed by means of filtration. The reaction mixture was evaporated under reduced pressure and purified using preparative TLC with a CH_2_Cl_2_/MeOH (10:1, *v/v*) system, to obtain the compounds **15a**–**15f**.

##### 2.1.7.1. (22 R,25R)-(2-aminoethyl-4-(bis(2-chloroethyl)amino)benzamido)-3β-hydroxy-5-en-furostan-26-amide (15a)

White powder, yield 92.3%. HPLC purity 95.05%. ^1^H NMR (600 MHz, Chloroform-*d*, TMS) *δ*_H_: 7.74 (d, *J* = 9.0 Hz, 2H, Ph-H), 7.45 (*s,* 1H, NH), 6.86 (*s*, 1H, NH), 6.66 (d, *J* = 9.0 Hz, 2H, Ph-H), 5.33 (d, *J* = 4.7 Hz, 1H, H-6), 4.28 (*m*, 1H, H-16), 3.77 (*t*, *J* = 7.1 Hz, 4H, NCH_2_C*H*_2_Cl, ×2), 3.64 (*t*, *J* = 7.1 Hz, 4H, NCH_2_C*H*_2_Cl, ×2), 3.53 (*m*, 2H, NHCH_2_), 3.52 (*m*, 1H, H-3), 3.49 (*m*, 2H, NHCH_2_), 3.25 (*t*, *J* = 7.9 Hz, 1H, H-22), 2.40 (*m*, 1H, H-25), 1.10 (d, *J* = 7.0 Hz, 3H, 21-CH_3_), 1.01 (*s*, 3H, 19-CH_3_), 0.89 (d, *J* = 6.6 Hz, 3H, 27-CH_3_), 0.75 (*s*, 3H, 18-CH_3_). ^13 ^C NMR (150 MHz, Chloroform-*d*, TMS) *δ*_C_: 178.99 (-CONH), 167.64 (-CONH), 148.63 (Ph-C), 141.00 (C-5), 129.26 (Ph-C, ×2), 122.71 (Ph-C), 121.41 (C-6), 111.19 (Ph-C, ×2), 90.92 (C-22), 83.57 (C-16), 71.82 (C-3), 64.71 (C-17), 57.07 (C-14), 53.46 (NCH_2_*C*H_2_Cl, ×2), 50.18 (C-9), 42.38 (C-4), 40.79 (C-12), 40.64 (NHCH_2_), 40.32 (N*C*H_2_CH_2_Cl, ×2), 39.49 (C-13), 39.31 (NHCH_2_), 38.32 (C-20), 37.37 (C-1), 36.71 (C-10), 36.05 (C-25), 32.52 (C-7), 32.10 (C-15), 31.72 (C-8), 31.06 (C-23), 30.83 (C-24), 27.33 (C-2), 20.79 (C-11), 19.57 (C-19), 18.75 (C-21), 18.03 (C-27), 16.57 (C-18). HRMS (ESI): *m*/*z* calculated for C_40_H_60_Cl_2_N_3_O_4_ [M + H]^+^: 716.3955, found: 716.3978.

##### 2.1.7.2. (22 R,25R)-(3-aminopropyl-4-(bis(2-chloroethyl)amino)benzamido)-3β-hydroxy-5-en- furostan-26-amide (15b)

White powder, yield 89.9%. HPLC purity 97.56%. ^1^H NMR (600 MHz, Chloroform-*d*, TMS) *δ*_H_: 7.84 (d, *J* = 6.7 Hz, 2H, Ph-H), 6.70 (d, *J* = 6.7 Hz, 2H, Ph-H), 5.34 (br s, 1H, H-6), 4.32 (br s, 1H, H-16), 3.78 (*t*, *J* = 6.7 Hz, 4H, NC*H*_2_CH_2_Cl, ×2), 3.64 (*t*, *J* = 6.7 Hz, 4H, NCH_2_C*H*_2_Cl, ×2), 3.51 (*m*, 1H, H-3), 3.45 (*m*, 2H, NHCH_2_), 3.35–3.33 (*m*, 3H, NHCH_2_, H-22), 2.45 (*m*, 1H, H-25), 1.16 (d, *J* = 6.0 Hz, 3H, 21-CH_3_), 1.02 (*s*, 3H, 19-CH_3_), 0.97 (d, *J* = 5.5 Hz, 3H, 27-CH_3_), 0.78 (*s*, 3H, 18-CH_3_). ^13 ^C NMR (150 MHz, Chloroform-*d*, TMS) *δ*_C_: 178.10 (-CONH), 167.29 (-CONH), 148.64 (Ph-C), 141.01 (C-5), 129.38 (Ph-C, ×2), 122.98 (Ph-C), 121.44 (C-6), 111.31 (Ph-C, ×2), 91.00 (C-22), 83.64 (C-16), 71.86 (C-3), 64.72 (C-17), 57.10 (C-14), 53.51 (NCH_2_*C*H_2_Cl, ×2), 50.20 (C-9), 42.40 (C-4), 40.87 (NHCH_2_), 40.84 (C-12), 40.32 (N*C*H_2_CH_2_Cl, ×2), 39.54 (C-13), 38.44 (C-20), 37.39 (C-1), 36.76 (C-10), 35.89 (NHCH_2_), 35.86 (C-25), 32.81 (NHCH_2_*C*H_2_), 32.54 (C-7), 32.13 (C-15), 31.75 (C-8), 31.01 (C-24), 30.16 (C-23), 27.35 (C-2), 20.81 (C-11), 19.59 (C-19), 18.84 (C-21), 18.26 (C-27), 16.59 (C-18). HRMS (ESI): *m*/*z* calculated for C_41_H_62_Cl_2_N_3_O_4_ [M + H]^+^: 730.4112, found: 730.4132.

##### 2.1.7.3. (22 R,25R)-(3-aminoazetidinyl-4-(bis(2-chloroethyl)amino)benzamido)-3β-hydroxy-5-en-furostan-26-amide (15c)

White powder, yield 91.0%. HPLC purity 95.57%. ^1^H NMR (600 MHz, Chloroform-*d*, TMS) *δ*_H_: 7.81 (br s, 2H, Ph-H), 6.68 (br s, 2H, Ph-H), 5.33 (d, *J* = 4.5 Hz, 1H, H-6), 4.88 (*m*, 1H, NHCH), 4.56 (*m*, 1H, NC*H*H), 4.46 (*m*, 1H, NC*H*H), 4.28 (*m*, 1H, H-16), 4.14 (*m*, 1H, NCH*H*), 4.08 (*m*, 1H, NCH*H*), 3.79 (*t*, *J* = 6.7 Hz, 4H, NC*H*_2_CH_2_Cl, ×2), 3.64 (*t*, *J* = 6.7 Hz, 4H, NCH_2_C*H*_2_Cl, ×2), 3.51 (*m*, 1H, H-3), 3.28 (*m*, 1H, H-22), 2.38 (*m*, 1H, H-25), 1.10 (d, *J* = 5.2 Hz, 3H, 21-CH_3_), 1.01 (*s*, 3H, 19-CH_3_), 0.98 (d, *J* = 5.0 Hz, 3H, 27-CH_3_), 0.78 (*s*, 3H, 18-CH_3_). ^13 ^C NMR (150 MHz, Chloroform-*d*, TMS) *δ*_C_: 177.01 (-CON), 167.07 (-CONH), 149.08 (Ph-C), 140.91 (C-5), 129.51 (Ph-C, ×2), 122.06 (Ph-C), 121.56 (C-6), 111.31 (Ph-C, ×2), 90.19 (C-22), 83.41 (C-16), 71.86 (C-3), 65.15 (C-17), 57.09 (C-14), 53.92 (NCH_2_, ×2), 53.43 (NCH_2_*C*H_2_Cl, ×2), 50.22 (C-9), 42.40 (C-4), 40.80 (C-12), 40.31 (N*C*H_2_CH_2_Cl, ×2), 39.82 (NHCH), 39.57 (C-13), 38.22 (C-20), 37.38 (C-1), 36.74 (C-10), 35.56 (C-25), 32.39 (C-7), 32.12 (C-15), 31.75 (C-8), 31.73 (C-24), 31.38 (C-23), 27.35 (C-2), 20.84 (C-11), 19.57 (C-19), 19.05 (C-21), 17.57 (C-27), 16.65 (C-18). HRMS (ESI): *m*/*z* calculated for C_41_H_60_Cl_2_N_3_O_4_ [M + H]^+^: 728.3955, found: 728.3965.

##### 2.1.7.4. (22 R,25R)-((S)-3-aminopyrrolidyl-4-(bis(2-chloroethyl)amino)benzamido)-3β-hydroxy-5-en-furostan-26-amide (15d)

White powder, yield 90.7%. HPLC purity 97.31%. ^1^H NMR (600 MHz, Chloroform-*d*, TMS) *δ*_H_: 7.69 (d, *J* = 8.8 Hz, 2H, Ph-H), 6.66 (d, *J* = 8.8 Hz, 2H, Ph-H), 6.19 (*s*, 1H, NH), 5.33 (d, *J* = 4.7 Hz, 1H, H-6), 4.66 (*m*, 1H, NHCH), 4.28 (*m*, 1H, H-16), 3.83 (*m*, 1H, NC*H*H), 3.78 (*t*, *J* = 6.7 Hz, 4H, NC*H*_2_CH_2_Cl, ×2), 3.69 (*m*, 1H, NCH*H*), 3.64 (*t*, *J* = 6.7 Hz, 4H, NCH_2_C*H*_2_Cl, ×2), 3.57 (*m*, 1H, NCH*H*), 3.51 (*m*, 1H, H-3), 3.46 (*m*, 1H, NC*H*H), 3.29 (*m*, 1H, H-22), 2.60 (*m*, 1H, H-25), 1.07 (d, *J* = 6.9 Hz, 3H, 21-CH_3_), 1.01 (*s*, 3H, 19-CH_3_), 0.97 (d, *J* = 6.7 Hz, 3H, 27-CH_3_), 0.78 (*s*, 3H, 18-CH_3_). ^13 ^C NMR (150 MHz, Chloroform-*d*, TMS) *δ*_C_: 175.85 (-CON), 167.06 (-CONH), 148.96 (Ph-C), 140.95 (C-5), 129.19 (Ph-C, ×2), 122.45 (Ph-C), 121.54 (C-6), 111.26 (Ph-C, ×2), 90.35 (C-22), 83.37 (C-16), 71.84 (C-3), 65.19 (C-17), 57.08 (C-14), 53.42 (NCH_2_*C*H_2_Cl, ×2), 52.62 (NCH_2_), 51.03 (NHCH), 50.21 (C-9), 44.01 (NCH_2_), 42.40 (C-4), 40.79 (C-12), 40.27 (N*C*H_2_CH_2_Cl, ×2), 39.57 (C-13), 38.24 (C-20), 38.15 (NCH_2_*C*H_2_), 37.39 (C-1), 36.75 (C-10), 35.88 (C-25), 32.38 (C-7), 32.13 (C-15), 31.73 (C-8), 31.57 (C-24), 31.43 (C-23), 27.34 (C-2), 20.82 (C-11), 19.57 (C-19), 19.07 (C-21), 17.79 (C-27), 16.61 (C-18). HRMS (ESI): *m*/*z* calculated for C_42_H_62_Cl_2_N_3_O_4_ [M + H]^+^: 742.4112, found: 742.4132.

##### 2.1.7.5. (22 R,25R)-(1-piperazinyl-4-(bis(2-chloroethyl)amino)benzamido)-3β-hydroxy-5-en-furostan-26-amide (15e)

White powder, yield 93.8%. HPLC purity 98.33%. ^1^H NMR (600 MHz, Chloroform-*d*, TMS) *δ*_H_: 7.36 (d, *J* = 8.8 Hz, 2H, Ph-H), 6.67 (d, *J* = 8.8 Hz, 2H, Ph-H), 5.33 (d, *J* = 4.0 Hz, 1H, H-6), 4.28 (*m*, 1H, H-16), 3.77 (*t*, *J* = 7.0 Hz, 4H, NC*H*_2_CH_2_Cl, ×2), 3.71–3.51 (*m*, 12H, NCH_2_C*H*_2_Cl, ×2, NCH_2_, ×4), 3.50 (*m*, 1H, H-3), 3.29 (*m*, 1H, H-22), 2.77 (*m*, 1H, H-25), 1.11 (d, *J* = 6.7 Hz, 3H, 21-CH_3_), 1.01 (*s*, 3H, 19-CH_3_), 0.97 (d, *J* = 6.7 Hz, 3H, 27-CH_3_), 0.77 (*s*, 3H, 18-CH_3_). ^13 ^C NMR (150 MHz, Chloroform-*d*, TMS) *δ*_C_: 175.32 (-CON), 170.88 (-CON), 147.75 (Ph-C), 141.00 (C-5), 129.94 (Ph-C, ×2), 123.59 (Ph-C), 121.47 (C-6), 111.33 (Ph-C, ×2), 90.13 (C-22), 83.36 (C-16), 71.79 (C-3), 65.14 (C-17), 57.06 (C-14), 53.43 (NCH_2_*C*H_2_Cl, ×2), 50.19 (C-9), 45.59 (NCH_2_, ×2), 42.38 (C-4), 42.01 (NCH_2_, ×2), 40.79 (C-12), 40.28 (N*C*H_2_CH_2_Cl, ×2), 39.53 (C-13), 38.23 (C-20), 37.37 (C-1), 36.74 (C-10), 35.36 (C-25), 32.41 (C-7), 32.13 (C-15), 31.74 (C-8), 31.52 (C-24), 31.13 (C-23), 27.33 (C-2), 20.80 (C-11), 19.56 (C-19), 19.00 (C-21), 17.92 (C-27), 16.59 (C-18). HRMS (ESI): *m*/*z* calculated for C_42_H_62_Cl_2_N_3_O_4_ [M + H]^+^: 742.4112, found: 742.4124.

##### 2.1.7.6. (22 R,25R)-(4–(1-homopiperazinyl)-4-(bis(2-chloroethyl)amino)benzamido)-3β-hydroxy-5-en-furostan-26-amide (15f)

White powder, yield 94.5%. HPLC purity 98.57%. ^1^H NMR (600 MHz, Chloroform-*d*, TMS) *δ*_H_: 7.32 (d, *J* = 8.7 Hz, 2H, Ph-H), 6.64 (d, *J* = 8.7 Hz, 2H, Ph-H), 5.33 (d, *J* = 4.7 Hz, 1H, H-6), 4.28 (*m*, 1H, H-16), 3.75 (*t*, *J* = 7.0 Hz, 4H, NC*H*_2_CH_2_Cl, ×2), 3.69–3.50 (*m*, 12H, NCH_2_C*H*_2_Cl, ×2, NCH_2_, ×4), 3.49 (*m*, 1H, H-3), 3.29 (*m*, 1H, H-22), 2.74 (*m*, 1H, H-25), 1.11 (d, *J* = 6.8 Hz, 3H, 21-CH_3_), 1.01 (*s*, 3H, 19-CH_3_), 0.97 (d, *J* = 6.5 Hz, 3H, 27-CH_3_), 0.77 (*s*, 3H, 18-CH_3_). ^13 ^C NMR (150 MHz, Chloroform-*d*, TMS) *δ*_C_: 176.46 (-CON), 171.97 (-CON), 147.31 (Ph-C), 140.99 (C-5), 130.00 (Ph-C, ×2), 124.85 (Ph-C), 121.49 (C-6), 111.30 (Ph-C, ×2), 90.27 (C-22), 83.35 (C-16), 71.81 (C-3), 65.19 (C-17), 57.06 (C-14), 53.56 (NCH_2_), 53.46 (NCH_2_*C*H_2_Cl, ×2), 50.20 (C-9), 50.03 (NCH_2_), 48.43 (NCH_2_), 47.98 (NCH_2_), 42.39 (C-4), 40.78 (C-12), 40.32 (N*C*H_2_CH_2_Cl, ×2), 39.54 (C-13), 38.22 (C-20), 37.38 (C-1), 36.75 (C-10), 35.86 (C-25), 32.36 (C-7), 32.13 (C-15), 31.87 (C-8), 31.74 (C-24), 31.42 (C-23), 29.44 (NCH_2_*C*H_2_), 27.33 (C-2), 20.81 (C-11), 19.56 (C-19), 19.05 (C-21), 18.72 (C-27), 16.57 (C-18). HRMS (ESI): *m*/*z* calculated for C_43_H_64_Cl_2_N_3_O_4_ [M + H]^+^: 756.4268, found: 756.4281.

### 2.2. Cells and cell culture conditions

Human hepatoma carcinoma HepG2, breast carcinoma MCF-7, cervical cancer HeLa, and gastric epithelial GES-1 cells were acquired from America Type Culture Collection (USA). HepG2 and GES-1 cells were cultured in Dulbecco’s Modified Eagle’s Medium supplemented with 10% foetal bovine serum (FBS). MCF-7 cells were incubated in Minimum Essential Medium supplemented with 10% FBS. HeLa cells were cultured in Roswell Park Memorial Institute medium supplemented with 10% FBS. All cells were maintained in a humidified incubator with 5% CO_2_, at 37 °C.

### 2.3. MTT assay

The inhibitory activities of the target hybrids (**8**–**10**, **14a**–**14f**, and **15a**–**15f**) were tested using the MTT method, as described previously^23^. HepG2, MCF-7, HeLa, and GES-1 cells were seeded into 96-well plates, at a density of 5 × 10^3^/well. After 24 h, the cells were treated with serially diluted concentrations of the test compounds. After treatment for 48 h, 10 µL of MTT (5 mg/mL) was added into each well and incubation was continued for another 4 h. The medium was then removed and 100 µL of dimethyl sulfoxide (DMSO) was added into the wells, to dissolve the formazan crystals. Finally, the absorbance was read on a microplate reader (iMark™, Bio-Rad, USA), at the wavelength of 492 nm.

### 2.4. Flow cytometric analysis of cell cycle distribution

HepG2 cells were seeded into 6-well plates and allowed to grow for a period of 12 h. After incubation with different concentrations of **14f** (0, 5, 10, and 20 µM) for 24 h, the cells were trypsinized, washed with phosphate-buffered saline (PBS), and fixed in 1.5 ml of 75% ethanol for the night, at 4 °C, following which RNase and propidium iodide (PI) (Multi Sciences Biotech Co. Ltd.) were added to the cells. The cell cycle distribution and data processing were analysed using a flow cytometer (FACSCalibur™, BD Biosciences, USA).

### 2.5. Assessment of changes in cell morphology

First, HepG2 cells were plated into 6-well plates, at a density of 1 × 10^6^ cells/well. Thereafter, **14f** (0, 5, 10, and 20 µM) was added to the cells and they were incubated with it for 48 h; 0.1% DMSO was used as a vehicle control. The cellular morphology was then observed and photographed using a light microscope (Olympus, 1 × 51, Japan). Next, HepG2 cells were incubated with **14f** (0, 5, 10, and 20 µM) for 48 h, after which the cells were collected and stained with a mixture of Hoechst 33342 and PI in buffer solution, for 20 min at room temperature. Finally, the cell samples were studied and photographed under a fluorescence microscope (Obeserve.A1, Zeiss, Germany).

### 2.6. Assessment of apoptosis using flow cytometry

Briefly, HepG2 cells were incubated in 6-well plates for 24 h, and then treated with **14f** (0, 5, 10, and 20 µM) for 48 h. Following that, the cells were washed once with pre-cold PBS, re-suspended in 300 µL of buffer solution, and stained with 10 µL of Annexin V-FITC and 5 µL of PI staining solution, under the conditions of 37 °C and absence of any light source, for 10 min. Finally, the samples were immediately measured using a flow cytometer. The percentages of apoptotic cells were determined on the CellQuest™ software (BD Biosciences).

### 2.7. Evaluation of MMP (δψm)

HepG2 cells were plated into 6-well plates for 12 h. Following treatment with varying concentrations of **14f** for 48 h, the cells were incubated with JC-1 dyeing working solution for 0.5 h at room temperature, then washed with staining buffer 3 times and re-suspended in 300 µL of staining buffer. The cells were finally assessed and analysed using a flow cytometer (FACSCalibur™).

### 2.8. Quantitative real-time polymerase chain reaction (qRT-PCR) assay

Total RNA was extracted from HepG2 cells using a Trizol™ reagent (Invitrogen, USA) and qRT-PCR was conducted on it, as documented before^24^. The purity and concentration of total RNA were checked using a spectrophotometer, in terms of A_260_ nm/A_280_ nm. A PrimeScript™ RT reagent Kit (Takara Bio, Japan) was applied to synthesise cDNA, according to the manufacturer’s instructions. A Detection System (Q5, ABI, USA) was used to carry out the qRT-PCR, using a TB Green™ Kit (Takara Bio) and the cycling conditions recommended for all genes in the kit instructions. Assessment of gene expression was carried out using the 2^−ΔΔCt^ method. The primer sequences used are presented in Table 1.

**Table 1.** Primer sequences for qRT-PCR.

Gene	Sequence	Length (bp)
CDK2	Forward: 5′-TGCCTGATTACAAGCCAAGTTTCCC-3′	98
	Reverse: 5′-TTGCGATAACAAGCTCCGTCCATC-3′	
CDK4	Forward: 5′-TTGCCAGCCGAAACGATCAAGG-3′	123
	Reverse: 5′-TCCACCACTTGTCACCAGAATGTTC-3′	
CDK6	Forward: 5′-GTGACCAGCAGCGGACAAATAAAAC-3′	86
	Reverse: 5′-ACGACCACTGAGGTTAGAGCCATC-3′	
Cyclin D1	Forward: 5′-GCCCTCGGTGTCCTACTTCAAATG-3′	111
	Reverse: 5′-TCCTCCTCGCACTTCTGTTCCTC-3′	
Cyclin E1	Forward: 5′-ACACCAGCCACCTCCAGACAC-3′	174
	Reverse: 5′-CGCAACCACCTGCTCCACTTG-3′	
Bax	Forward: 5′-AGCTTCTTGGTGGACGCAT-3′	101
	Reverse: 5′-CAGAGGCGGGGTTTCATC-3′	
Bcl-2	Forward: 5′-GAGAAATCAAACAGAGGCCG-3′	106
	Reverse: 5′-CTGAGTACCTGAACCGGCA-3′	
Caspase 9	Forward: 5′-CATGCTCAGGATGTAAGCCA-3′	93
	Reverse: 5′-AGGTTCTCAGACCGGAAACA-3′	
Caspase 3	Forward: 5′-TCGCTTCCATGTATGATCTTTG-3′	110
	Reverse: 5′- CTGCCTCTTCCCCCATTCT-3′	

### 2.9. Determination of caspase activities

The activities of caspase 9 and caspase 3 in the cell lysates were evaluated as described previously^25^. The harvested HepG2 cells treated with varying concentrations of **14f** for 48 h were lysed using chilled 100 µL lysis buffer, to extract the total protein. The protein concentration was assessed using a Bradford Kit. The protein samples were incubated with assay buffer and colorimetric substrate (Ac-DEVD-pNA or Ac-LEHD-pNA) at room temperature for 2 h, according to the manufacturer’s protocol. The released pNAs were measured at the wavelength of 405 nm using a Multiskan**^®^** Spectrum system. The experiments were performed in triplicate.

### 2.10. Western blot

To analyse the effect of compound **14f** on protein levels, western blot assay was employed, as elaborated previously^26^. The proteins were acquired by collecting HepG2 cells treated with varying concentrations of **14f** for 48 h and adding them to lysis buffer, in an ice bath for 30 min. After quantitation of protein concentration using BCA assay, equal amounts of total protein were separated using 10% sodium dodecyl sulphate-polyacrylamide gel electrophoresis and then transferred onto polyvinylidene fluoride membranes. The membranes were incubated with 5% bovine serum albumin solution for 1.5 h at 37 °C, followed by incubation with primary antibody overnight, and finally secondary antibody for 2 h at 37 °C. The blots were detected using enhanced chemiluminescence reagent with a fully automatic gel imaging system (ChemiDoc™ MP, Bio-Rad). The relative expression was calculated by normalising the expression of the control group to 1 for comparison.

### 2.11. Molecular docking study

Molecular docking studies were performed using GLIDE (2016, Schrödinger Suite)^27^. The crystal structures of Bcl-2 (PDB: 2O2F)^28^ were retrieved from the RCSB Protein Data Bank, and further prepared using the Protein Preparation Wizard tool implemented in the Schrödinger Suite, by adding all hydrogen atoms as well as missing side chains of residues and deleting all bound water. The ligands were built within Maestro BUILD (2016, Schrödinger Suite) and prepared using the LIGPREP module (2016, Schrödinger Suite)^27^. The Glide Grid was built using an inner box of dimensions 15 × 15 × 15 Å^3^ around the centroid of the ligand, assuming that the ligands to be docked were of a size similar to that of the co-crystallized ligand. This docking methodology has been validated by extracting the crystallographic bound ligand and re-docking it with the Glide module using extra precision. Different docking poses of ligands were generated and analysed for interpretation of the final results.

## 3. Results and discussion

### 3.1. Chemistry

**Scheme 1.**
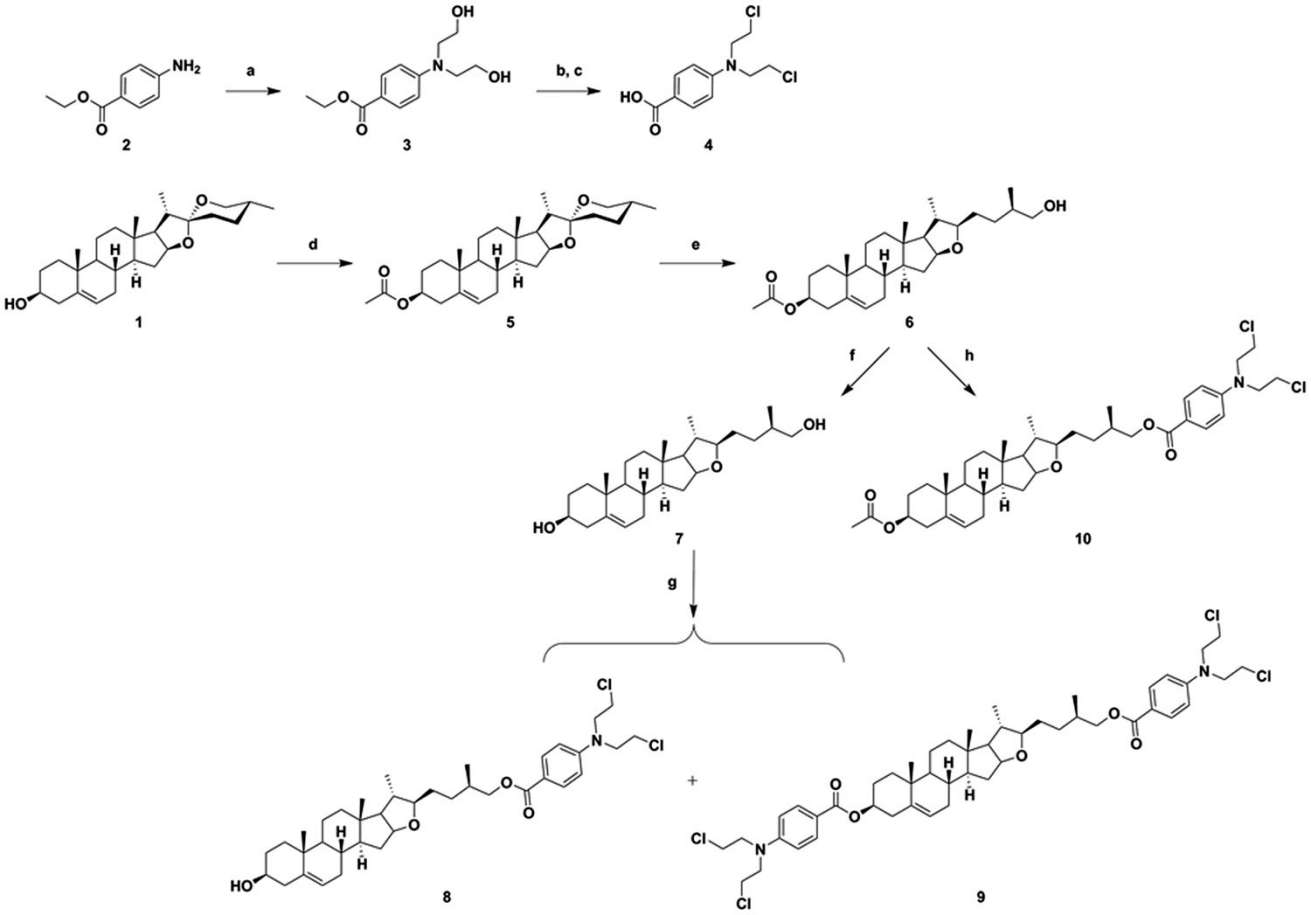
Synthesis of diosgenin–benzoic acid mustard trihybrids **8**−**10**. Reagents and conditions: (a) ethylene oxide, H_2_O, CH_3_COOH, rt, 24 h; (b) POCl_3_, 50 °C, 0.5 h; (c) 10% HCl, 12 h; (d) Ac_2_O, dry pyridine, dry CH_2_Cl_2_, rt, 6 h; (e) NaBH_3_CN, AcOH, CH_2_Cl_2_, rt, 8 h; (f) KOH, CH_3_OH, rt, 6 h; (g) (h) Benzoic acid mustard, EDCI, DMAP, CH_2_Cl_2_, rt, 24 h.

**Scheme 2.**
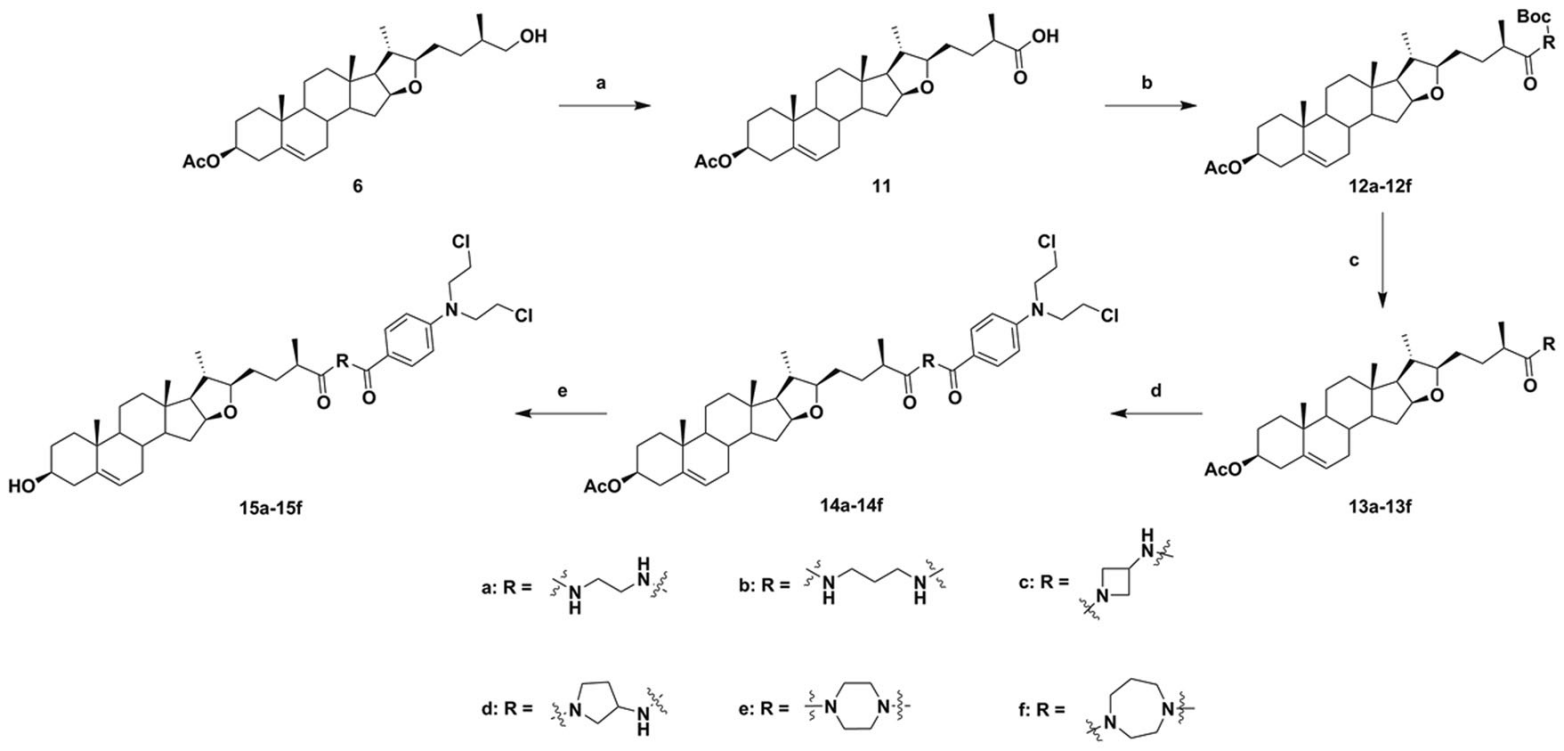
Synthesis of diosgenin–benzoic acid mustard hybrids **14a**−**14f** and **15a**−**15f**. Reagents and conditions: (a) Jones reagent, THF/acetone (1/1), rt, 3 h; (b) *N*-Boc-protected amines, TBTU, DIPEA, CH_2_Cl_2_, rt, 8 h; (c) CF_3_COOH, CH_2_Cl_2_, rt, 4 h; (d) Benzoic acid mustard, DMAP, EDCI, CH_2_Cl_2_, rt, 24 h; (e) NaOH, CH_3_OH/THF (1/1.5), rt, 6 h.

The structures and reaction conditions for the preparation of the new hybrids of DSG–benzoic acid mustard are depicted in Schemes 1 and 2. The benzoic acid mustard **4** was prepared according to a previously described method^20^. DSG derivative **6**, which was obtained using a procedure reported previously by our research group^22^, was saponified to obtain intermediate **7**. Intermediate **6** or **7** was coupled with benzoic acid mustard in CH_2_Cl_2_ using EDCI and DMAP as catalysts, to obtain the target DSG–benzoic acid mustard hybrids **8**, **9**, and **10** (Scheme 1).

Next, the C-26 hydroxyl of intermediate **6** was oxidised using Jones reagent, to obtain intermediate **11**. Further, the C-26 carboxyl of intermediate **11** was reacted with various *N*-Boc-protected amines using TBTU and DIPEA as coupling catalysts, to provide conjugates **12a**–**12f** (Scheme 2), which were converted to **13a**–**13f** by using CF_3_COOH for deprotection. The DSG–benzoic acid mustard hybrids **14a**–**14f** were prepared using the same methods as those for **10**. Finally, saponification of hybrids **14a**–**14f** resulted in the corresponding target DSG–benzoic acid mustard hybrids **15a**–**15f**. The structures of all new hybrids were fully corroborated using various spectroscopic methods, including HRMS and NMR spectroscopy (^1^H NMR and ^13 ^C NMR).

### 3.2. Biological testing

#### 3.2.1. *In vitro* anti-proliferative activity

The anti-proliferative activities of the synthesised target hybrids towards three human cancer cells HepG2 (hepatoma), MCF-7 (breast cancer), HeLa (cervical cancer) and normal GES-1 (stomach) cells were evaluated using MTT assay. Mitomycin C was selected as the positive control drug, and the results of this experiment are listed in Table 2. Benzoic acid mustard (**4**) was firstly introduced on the scaffold of DSG derivatives **6** and **7** via an ester bond. Unfortunately, the resulting hybrids **8**–**10** showed decreased inhibitory activities against the three cancer cells.

**Table 2.** Cytotoxic activities of target hybrids **8– 10**, **14a–14f** and **15a–15f** in different cell lines.

Compound	IC_50_^a^ (μM)				SI^b^
	HepG2	MCF-7	HeLa	GES-1	
**8**	>50	>50	>50	>100	N.D.
**9**	–	>50	>50	>100	N.D.
**10**	>50	28.06 ± 1.52	>50	>100	N.D.
**14a**	10.19 ± 1.01	48.33 ± 2.21	17.24 ± 2.81	>100	9.81
**14b**	25.29 ± 1.40	30.30 ± 2.08	>100	46.97 ± 2.34	1.86
**14c**	12.22 ± 1.09	24.59 ± 1.11	>100	80.79 ± 3.11	6.61
**14d**	17.47 ± 0.98	>100	>100	46.99 ± 3.24	2.69
**14e**	19.27 ± 1.29	42.69 ± 2.25	>50	58.76 ± 1.34	3.05
**14f**	2.26 ± 0.87	11.07 ± 1.01	41.63 ± 3.12	>100	>44.25
**15a**	13.25 ± 1.01	–	>50	83.17 ± 3.12	6.28
**15b**	14.00 ± 1.15	37.20 ± 2.23	49.81 ± 2.13	>100	>7.14
**15c**	14.57 ± 1.16	>100	>50	>100	>6.86
**15d**	>50	>100	>50	>100	N.D.
**15e**	13.99 ± 1.12	>50	28.46 ± 2.13	–	N.D.
**15f**	18.93 ± 0.93	>100	>50	>100	>5.28
**4**	–	25.58 ± 1.31	>100	>100	N.D.
Diosgenin	33.87 ± 1.37	23.91 ± 1.34	55.05 ± 2.11	>100	>2.95
Mitomycin C	32.63 ± 1.22	16.71 ± 1.02	22.03 ± 1.12	23.43 ± 1.11	0.72

^a^ IC_50_: concentrations inhibit 50% of cell growth measured by the MTT assay. The values are expressed as average ± SD of three independent experiments.

“–”: not active.

N.D.: Not determined.

^b^ SI = IC_50_ for GES-1 cell line/IC_50_ for HepG2 cell line.

Compared to DSG and **4**, all the target hybrids **14a**–**14f** and **15a**–**15f** (except for **15d**) displayed moderate to potent inhibitory activities in HepG2 cells, with IC_50_ values in the range of 2.26–25.29 µM. The results showed that introducing amide-amide bonds into the two pharmacophores of DSG and **4**, to afford hybrids **14a**–**14f** and **15a**–**15f**, could improve their anti-proliferative potency. However, changing the linkers does not seem to regularly affect activities. Among them, compound **14f** (IC_50_ = 2.26 µM), which comprised of DSG derivative **11** substituted by the moiety of **4** through a homopiperazinyl linker, showed obvious selective anti-proliferative activity against HepG2 cells. Its potency was approximately 15.0-fold higher than that of DSG (IC_50_ = 33.87 µM), and 14.4-fold higher than that of Mitomycin C (IC_50_ = 32.63 µM). The cytotoxic activity of **14f** was greatly improved in comparison with those of the counterparts of DSG–amino acid–benzoic acid mustard trihybrids **12a**–**12g** (IC_50_ > 10.43 µM)^25^.

In MCF-7 cell line, hybrids **14a**–**14f** (IC_50_ > 11.07 µM) generally exhibited higher inhibitory activity than hybrids **15a**–**15f** (IC_50_ > 37.20 µM). These data showed that the group of acetyl substituted at the C-3 OH was beneficial for compounds with inhibitory potency against MCF-7 cell line. Among them, only hybrid **14f** possessing a homopiperazinyl linker exhibited moderate anti-proliferative activity, with an IC_50_ value of 11.07 µM, and was about 1.7-fold more active than DSG (IC_50_ = 23.91 µM). The results also confirmed that the incorporation of homopiperazinyl between DSG derivative **11** and **4** results in favourable enhancement of anti-proliferative activity against MCF-7 cells, as compared to those of nitrogen-containing groups.

In HeLa cell line, conjugates **14a** (IC_50_ = 17.24 µM) and **15e** (IC_50_ = 28.46 µM) showed relatively stronger cytotoxicity than other conjugates and DSG (IC_50_ = 55.05 µM).

The selectivity index (SI), one of the important pharmaceutical parameters, was calculated to determine the toxicities of these hybrids against human normal gastric epithelial GES-1 cell line, as compared to those against hepatoma HepG2 cell line (Table 2). Among them, the most potent anti-proliferative hybrid **14f** showed considerable safety (SI > 44.25).

Taken together, among these DSG–benzoic acid mustard hybrids, hybrid **14f**, with the linkage of homopiperazinyl, showed higher anti-proliferative activity against HepG2 and MCF-7 cancer cells, which suggested that the introduction of homopiperazinyl between DSG derivative **11** and benzoic acid mustard is beneficial for anti-proliferative activity. Our findings are in good agreement with a report by Wolfram et al., which also showed that the linker of homopiperazinyl is essential for mitocanic triterpenoidic rhodamine B adducts that show high cytotoxicity against a panel of human tumour cell lines^29^. Hybrid **14f** showed the lowest IC_50_ value of 2.26 µM and low anti-proliferative activity against normal GES-1 cells, with an IC_50_ value > 100 µM. Hence, **14f** was selected for further investigation of the possible cellular mechanisms in HepG2 cell line.

#### 3.2.2. Effect of 14f on the cell cycle

**Figure 3.**
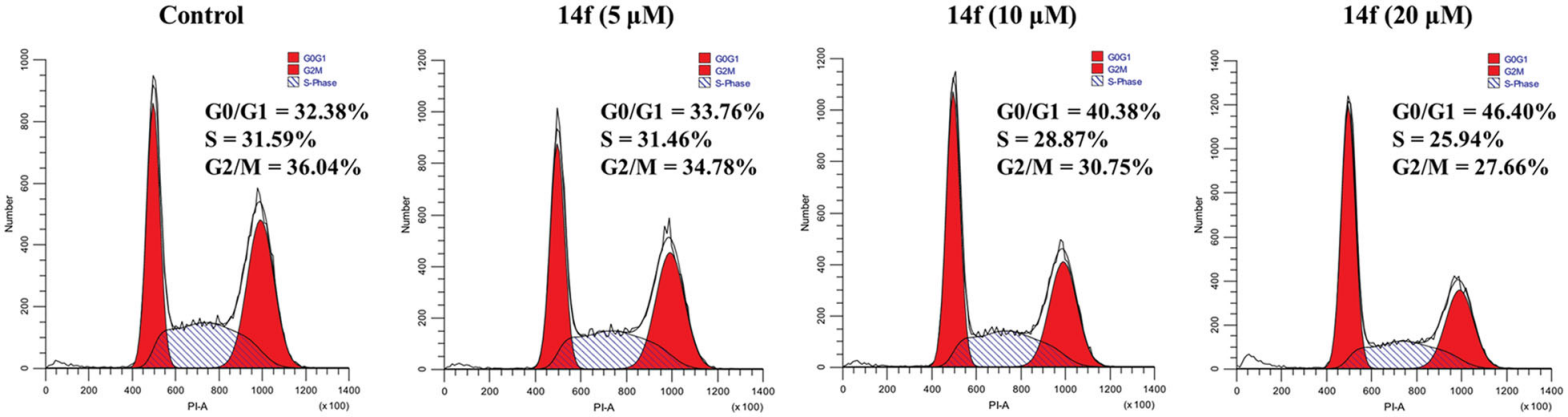
Analysis of the effects of **14f** on the cell cycle in HepG2 cells. HepG2 cells were treated with indicated concentrations (5, 10, and 20 μM) of **14f** for 24 h, harvested, stained with PI, and assessed using a flow cytometer.

Uninterrupted cell cycle progression is vital to the biochemical processes surrounding cell division and replication. Thus, blockade of the cell cycle is considered as an effective strategy in cancer therapy^30^. Previous studies have demonstrated that DSG arrests cell cycle and induces apoptosis in different cancer cells^5^^,^^7^^,^^31^^,^^32^. To determine whether the anti-proliferative effects of hybrid **14f** are caused by cell cycle arrest at a certain phase, the effects of different concentrations of **14f** on cell cycle progression were examined in HepG2 cells. Upon treatment with different concentrations (0, 5, 10, and 20 µM) of **14f**, 32.38, 33.76, 40.38, and 46.40% of the cells, respectively, were found to be in the G0/G1 phase (Figure 3). The results demonstrated that **14f** arrested the cell cycle at the G0/G1 phase in HepG2 cells.

**Figure 4.**
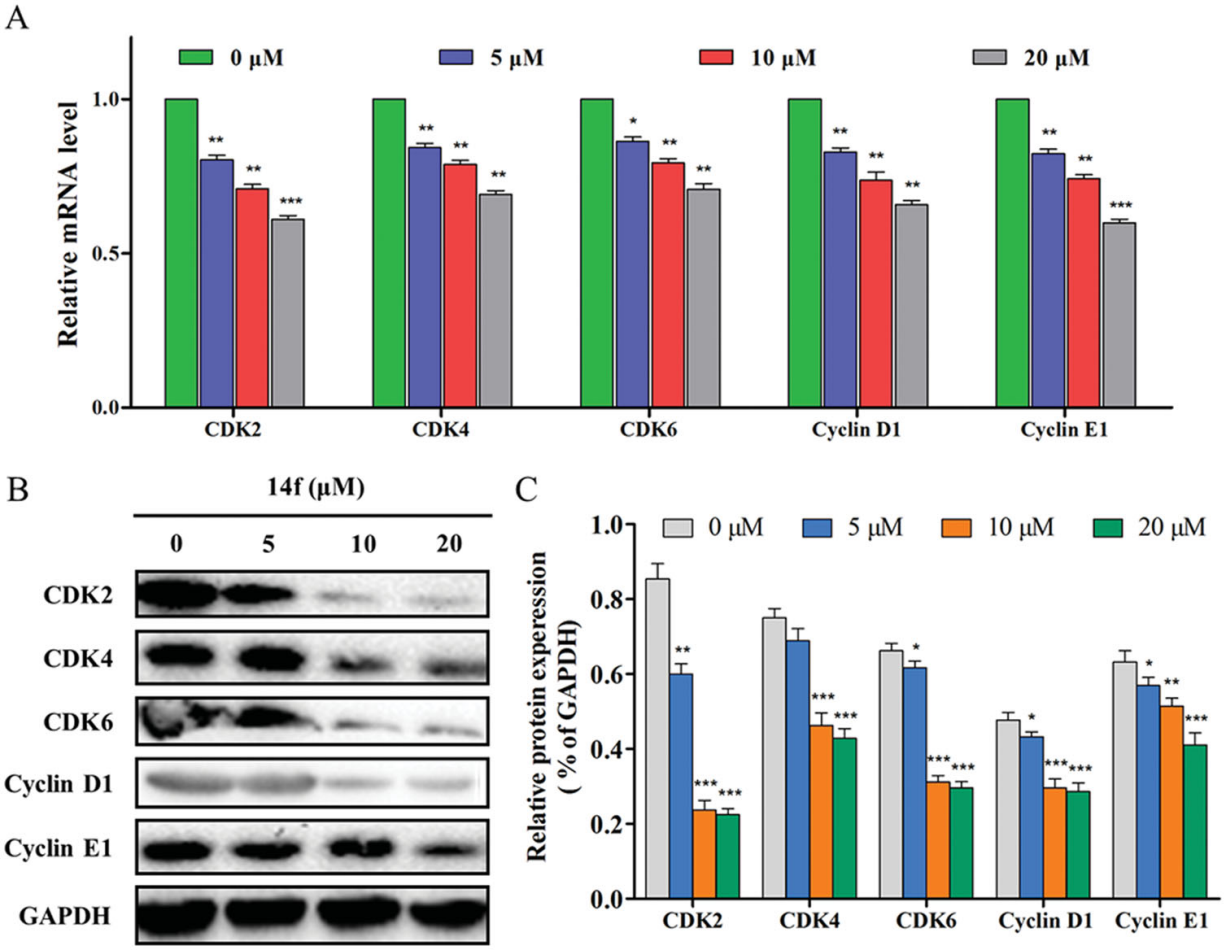
Effect of **14f** on the levels of cell cycle-related genes and proteins in HepG2 cells. GAPDH served as the loading control. (A) Relative mRNA level. (B) The expression of proteins was detected using Western blot. Values have been represented as mean ± SD (*n* = 3). *p<0.05; **p<0.01; ***p<0.001 versus control group.

Subsequently, the specific mechanism by which **14f** regulates the G0/G1 phase was further investigated at the transcription and translation levels. The effect of **14f** on the expression levels of several key cell cycle-related genes in HepG2 cells was determined using qRT-PCR. It was found that **14f** decreased the genes levels of CDK2, CDK4, CDK6, cyclin D1 and cyclin E1 (Figure 4(A)), all of which appear in the G0/G1 phase. In addition, upon performing western blot to detect the G0/G1 phase-related proteins, it was found that **14f** could concentration-dependently downregulate the expression levels of CDK2, CDK4, CDK6, cyclin D1, and cyclin E1 (Figure 4(B)). These findings suggested that the inhibition of HepG2 cells by **14f** was related to the induction of G0/G1 phase arrest.

#### 3.2.3. 14f promoted apoptosis in HepG2 cells

**Figure 5.**
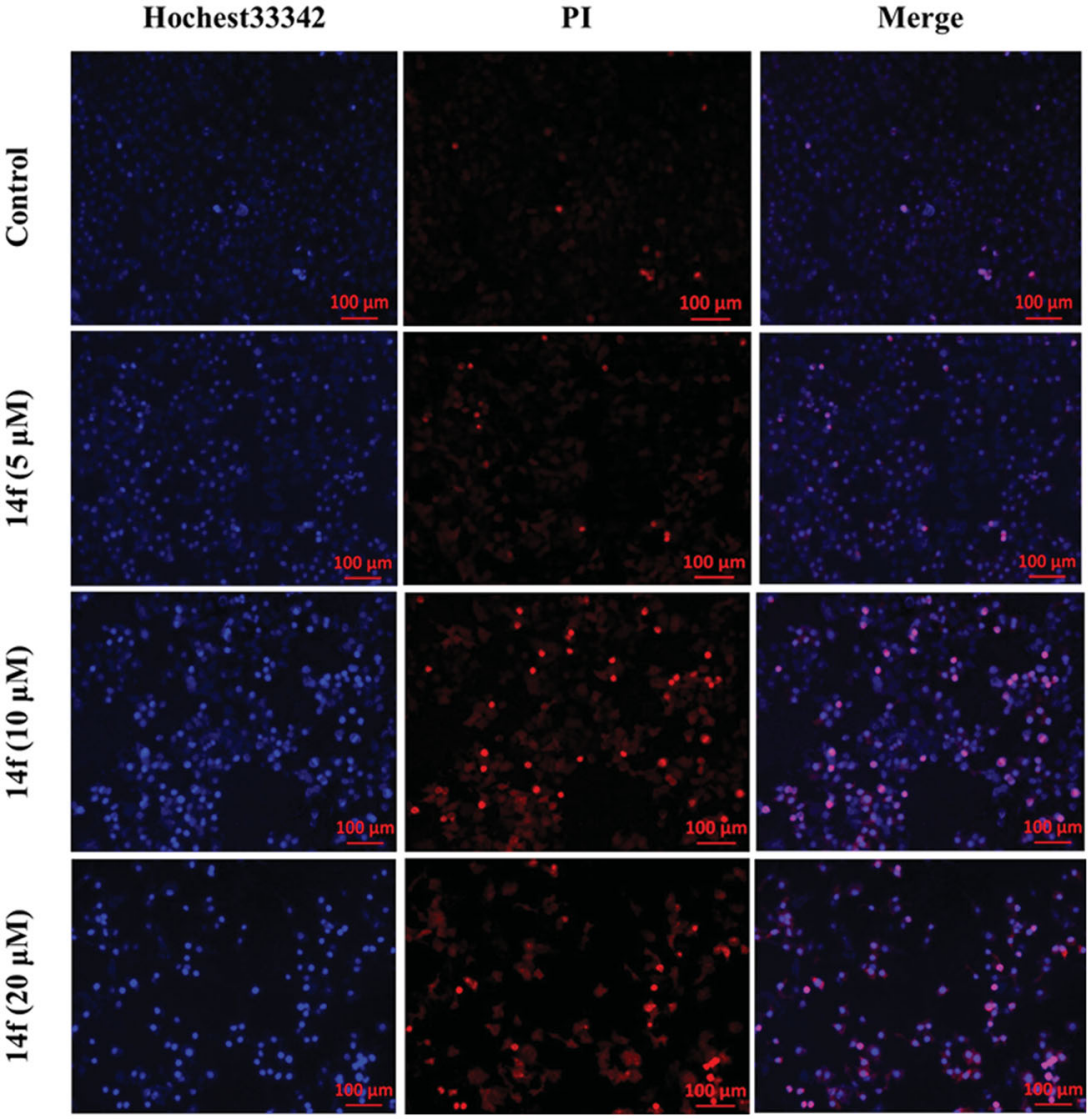
Fluorescence microscopy images of HepG2 cells stained using Hoechst 33342/PI. HepG2 cells were treated with different concentrations (5, 10, and 20 μM) of **14f** for 48 h.

Inducing apoptosis is an important strategy for cancer treatment. To assess whether the **14f** could promote the apoptosis of HepG2 cells, we detected the apoptosis of HepG2 cells upon treatment with various concentrations of **14f**. First, we observed the morphological changes in HepG2 cells under an inversion microscope. As seen in Figure S1, microscopic observation showed that HepG2 cells presented a significant round shape with shrinkage of the cell membrane upon treatment with **14f**, accompanied by a prominent decrease in the number of cells. These phenomena were further examined using the Hoechst 33342/PI staining assay, the results for which showed that upon treatment with **14f**, there was an increase in the apoptosis of HepG2 cells, indicated by deep blue and red fluorescence, accompanied by the observation of apoptosis bodies (Figure 5). Collectively, the above observation demonstrated that **14f** treatment induced apoptosis in HepG2 cells.

**Figure 6.**
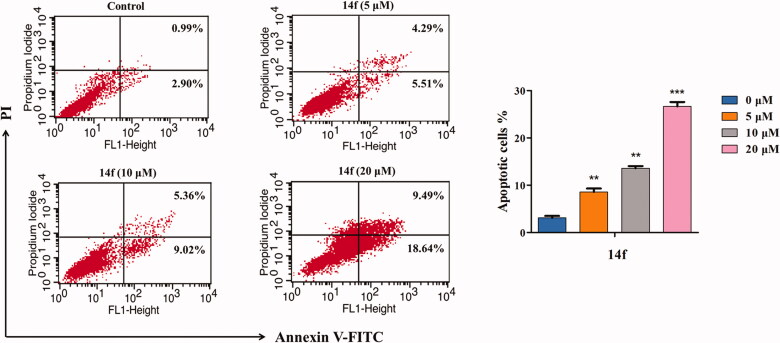
Flow cytometry analysis of **14f**-induced apoptosis in HepG2, as assessed using Annexin V-FITC/PI assay. The percent of apoptotic cells is indicated on the right. Values have been represented as mean ± SD (*n* = 3). **p<0.01; ***p<0.001 versus control group.

To further obtain an accurate evaluation of the **14f**-mediated apoptosis of HepG2 cells, cells treated with different concentrations of **14f** were analysed using flow cytometry, post Annexin V-FITC/PI double staining. As seen in Figure 6, with increasing concentrations of **14f**, the number of apoptotic cells increased from 3.89% to 9.80%, 14.38%, and 28.13%, while the number of surviving cells decreased correspondingly, in a concentration-dependent manner. These data demonstrated that **14f** promoted apoptosis in HepG2 cells as well.

#### 3.2.4. 14f decreased the MMP (δψm) of HepG2 cells

**Figure 7.**
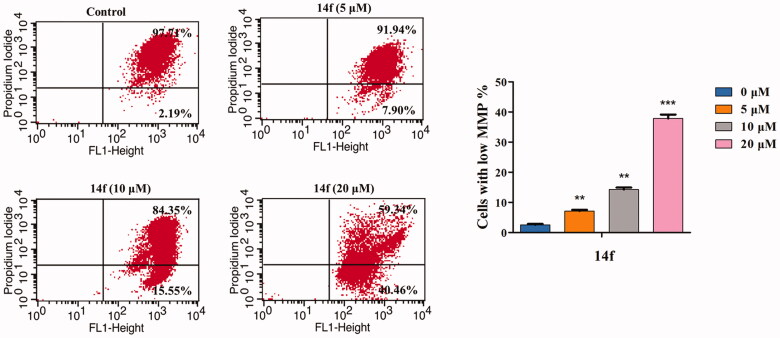
**14f** induced the collapse of mitochondrial membrane potential (ΔΨm) in HepG2 cells. After treatment with **14f** for 48 h, the cells stained with JC-1 were determined by means of flow cytometric analysis. Values have been represented as mean ± SD (*n* = 3). **p<0.01; ***p<0.001 versus control group.

Mitochondrion plays an important role in regulating cellular functions. Loss of MMP is a sign of the apoptotic process in cells. MMP collapse represents the initiation and activation of apoptosis by the intrinsic pathway^33^. In the present study, JC-1 staining was conducted to investigate whether **14f**-induced HepG2 cell apoptosis was associated with the loss of MMP. As seen in Figure 7, there was a **14f** dose-dependent increase in the percentage of mitochondrial depolarisation (7.90% at 5 µM, 15.55% at 10 µM, and 40.46% at 20 µM). The result indicated that **14f** induced apoptosis in HepG2 cells by destroying the mitochondrial integrity and decreasing MMP.

#### 3.2.5. 14f induced apoptosis in HepG2 cells through the mitochondrial pathway

**Figure 8.**
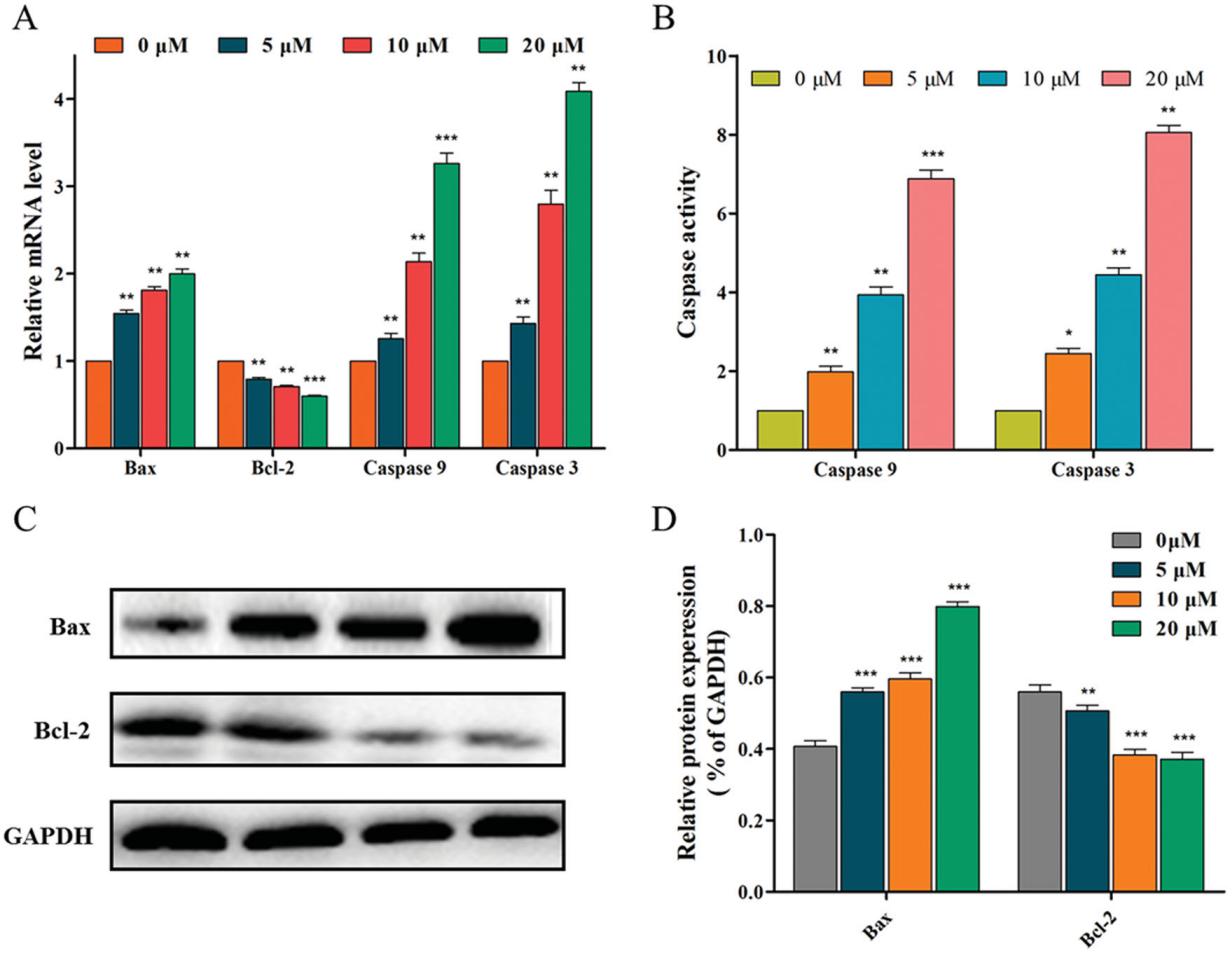
**14f** activated the mitochondrial pathway, to regulate the apoptosis of HepG2 cells. (A) Relative mRNA level. (B) The activities of caspase 9 and caspase 3 were detected using ELISA. (C) The relative protein expression levels were detected using western blot analyses. (D) Quantitative analysis of the protein expression data mentioned in (C). Values have been represented as mean ± SD (*n* = 3). **p<0.01; ***p<0.001 versus control group.

Based on the results, we hypothesised that **14f** might be a MMP disruptor, and further examined the effect of **14f** on mitochondrial pathway-related factors (such as anti-apoptotic protein Bcl-2 and pro-apoptotic protein Bax) and apoptotic effectors (such as caspase 9 and caspase 3), which play important roles in cell apoptosis^22^. Firstly, the expression of mitochondrial pathway-related genes was evaluated using qRT-PCR. As seen in Figure 8(A), **14f** treatment led to high gene expression of Bax, caspase 9, and caspase 3, while the gene expression of Bcl-2 was suppressed. Next, based on the qRT-PCR results, ELISA was further used to confirm whether the apoptosis-related enzymes, caspase 9 and caspase 3, are involved in the regulation of apoptosis. The results indicated that **14f** upregulated the enzyme activity of caspase 9 and caspase 3 (Figure 8(B)). Finally, western blot was conducted to investigate the protein expression levels of Bax and Bcl-2. As shown in Figure 8(C), **14f** upregulated the levels of Bax with associated downregulation of Bcl-2 levels (Figure 8(C)). Moreover, the quantitative analysis results were consistent with the trend of mRNA level expression (Figure 8(D)). These results suggested that **14f** triggered the mitochondrial pathway of apoptosis in HepG2 cells.

#### 3.2.6. Molecular docking studies

**Figure 9.**
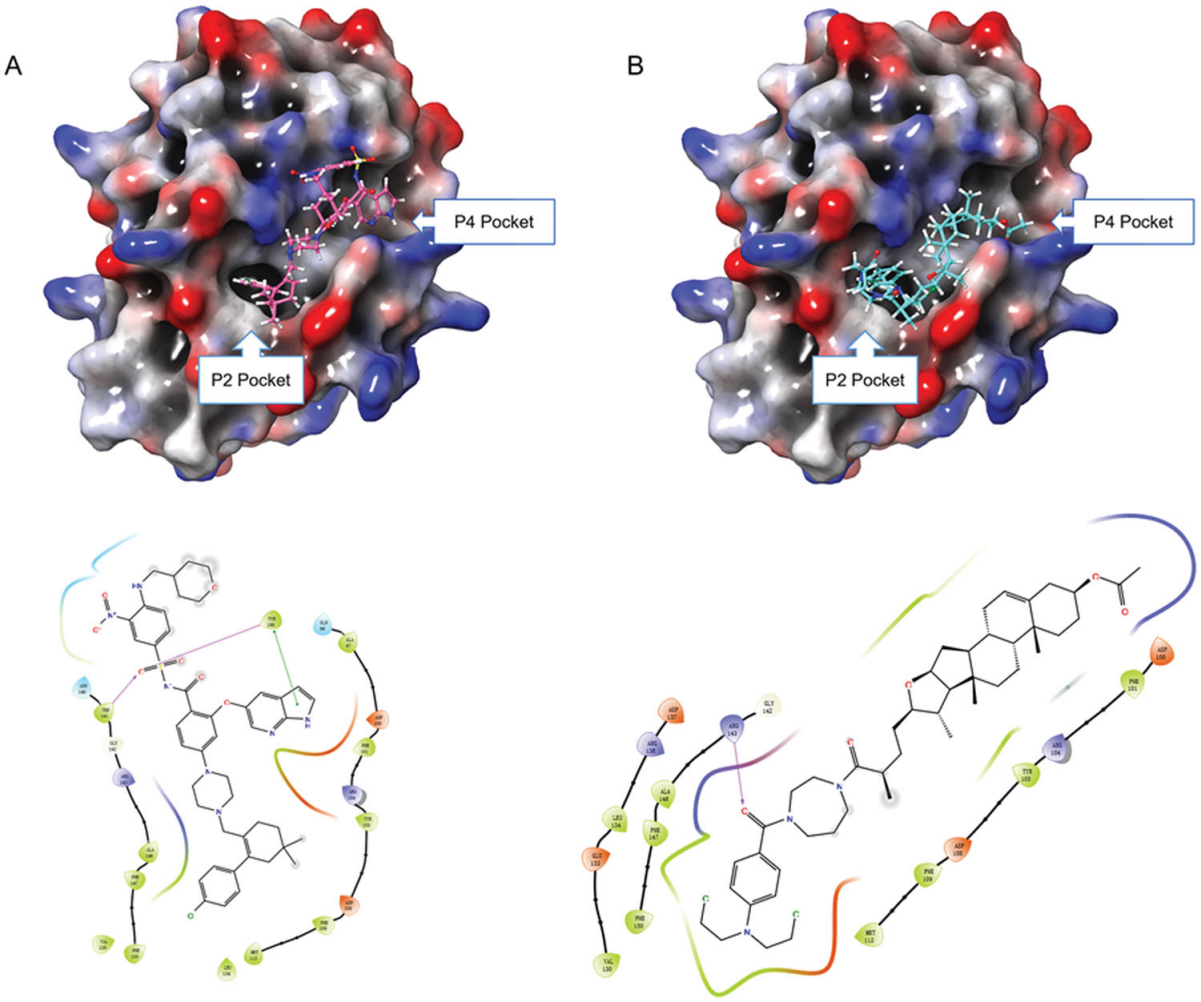
(A) Binding mode of ABT-199 to Bcl-2 protein; (B) Binding mode of **14f** to Bcl-2 protein.

Based on the downregulation of Bcl-2 by hybrid **14f**, we next explored the potential binding mode of **14f** with Bcl-2. High-affinity binding of pro-apoptotic proteins to Bcl-2 is largely mediated by protein-protein interactions in the P2 and P4 hydrophobic pockets. Thus, the Bcl-2-inhibiting inhibitory potency of small molecules is determined by the interactions of these inhibitors with P2 and P4 pockets^34^^,^^35^. Venetoclax, also known as ABT-199, was the first Bcl-2-selective inhibitor^35^. Our docking research showed that the tetrahydro-1, 1′-biphenyl fragment and 1H-pyrrolo[[2,3-b]] pyridine fragment of ABT-199 could occupy the P2 and P4 pockets, respectively (Figure 9). Coincidentally, similar behaviours could be observed in the bonding mode of **14f** to Bcl-2 protein. **14f** could lay on the deep hydrophobic binding pocket in the Bcl-2 groove, with the benzoic acid mustard moiety accommodating the P2 pocket and the acetyl moiety entering the P4 pocket. The experimental results showed that **14f** might have the ability to interact with Bcl-2, thereby inducing HepG2 cells apoptosis.

Altogether, the above results indicated that **14f** arrested the cell cycle at the G0/G1 phase and induced apoptosis in HepG2 cells through the mitochondrial apoptosis pathway. To comprehend the exact regulatory mechanism of **14f** against hepatoma, there is a need to carry out well-designed *in vivo* studies in the future.

## 4. Conclusion

In summary, fifteen novel DSG-benzoic acid mustard hybrids (**8**–**10**, **14a**–**14f**, and **15a**–**15f**) were designed and synthesised in the present study. This was followed by testing the cytotoxicity of these target hybrids against human cancer HepG2, MCF-7, and HeLa cell lines and human normal GES-1 cells. Most of them did not display significant cytotoxic activity. Several of these hybrids exhibited moderate to potent inhibitory activities against HepG2 cells. Among them, hybrid **14f** (IC_50_ = 2.26 µM) exhibited the most potent anti-proliferative activity in HepG2 cells, and displayed an efficacy that was 14.4-fold higher than that of DSG (IC_50_ = 32.63 µM). Moreover, **14f** exhibited good anti-proliferative selectivity between normal and tumour cells. SAR studies showed that the introduction of homopiperazinyl between DSG derivative **11** and benzoic acid mustard was beneficial for enhancing the anti-proliferative activity. Further investigations on the anti-tumour mechanism in HepG2 cells indicated that **14f** arrested the cell cycle at the G0/G1 phase, by regulating the cell cycle-related proteins (CDK4, CDK6, cyclin D1, CDK2, and cyclin E1), and induced apoptosis by mediating the apoptosis-related proteins (Bax, Bcl-2, caspase 9, and caspase 3). Furthermore, molecular docking revealed that hybrid **14f** could bind at the deep hydrophobic binding pocket in the Bcl-2 groove, indicating that the potential target of **14f** might be Bcl-2. Therefore, conjugating DSG with benzoic acid mustard might be an efficient strategy for the modification of this class of natural products.

## Supplementary Material

Supplemental Material
