# Supplementary material for: Design, synthesis and biological evaluation of novel diosgenin–benzoic acid mustard hybrids with potential anti-proliferative activities in human hepatoma HepG2 cells
Source: J Enzyme Inhib Med Chem. 2022 Jun 2;37(1):1299–314. doi: 10.1080/14756366.2022.2070161 (PMC9176691; doi:10.1080/14756366.2022.2070161)
Supplement: Supplemental Material [file IENZ_A_2070161_SM9289.pdf]

Design, synthesis and biological evaluation of novel diosgenin–benzoic acid mustard hybrids with potential anti-proliferative activities in human hepatoma HepG2 cells

Wenbao Wang[a,b,*], Jinling Zhang[a], Yanzhao Tian[b], Liwei Ma[a], Lin Zhou[b], Hao Sun[b], Yukun Ma[a], Huiling Hou[a], Xiaoli Wang[a], Jin Ye[a], Xiaobo Wang[b,*]

[a]*Qiqihar Medical University, Qiqihar 161006, Heilongjiang, PR China*

[b]*Chinese People's Liberation Army Logistics Support Force No. 967 Hospital, Dalian 116021, PR China*

[*]Corresponding author. E-mail address: wangwenbao0824@163.com (W. Wang); wxbbenson0653@sina.com (X. Wang)

Supplementary Data

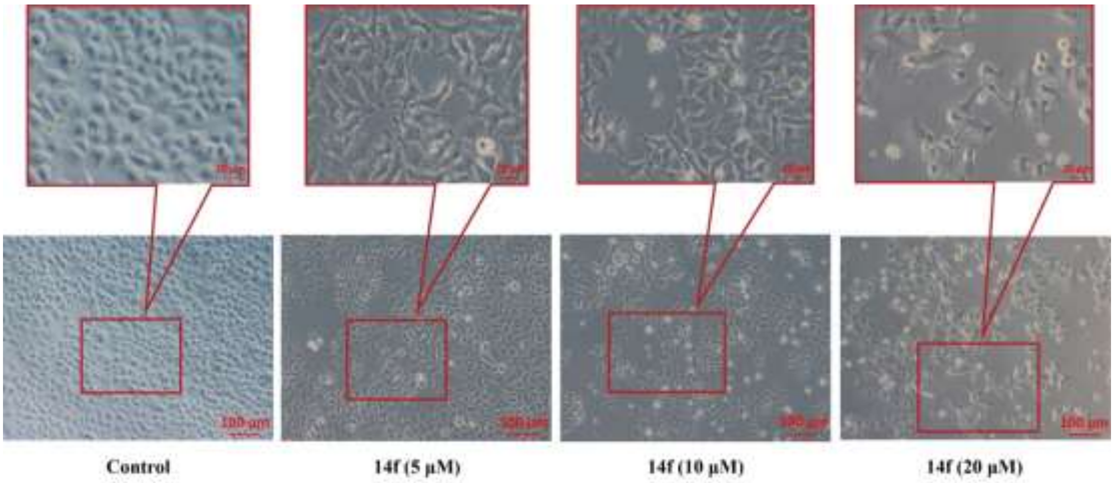

Figure S1. **14f** treatment induced apoptosis in HepG2 cells. HepG2 cells were treated with **14f** (5, 10 and 20 μM) for 48 h and photographed under a light microscope.

HR-MS, ^{1}H NMR and ^{13}C NMR spectrums

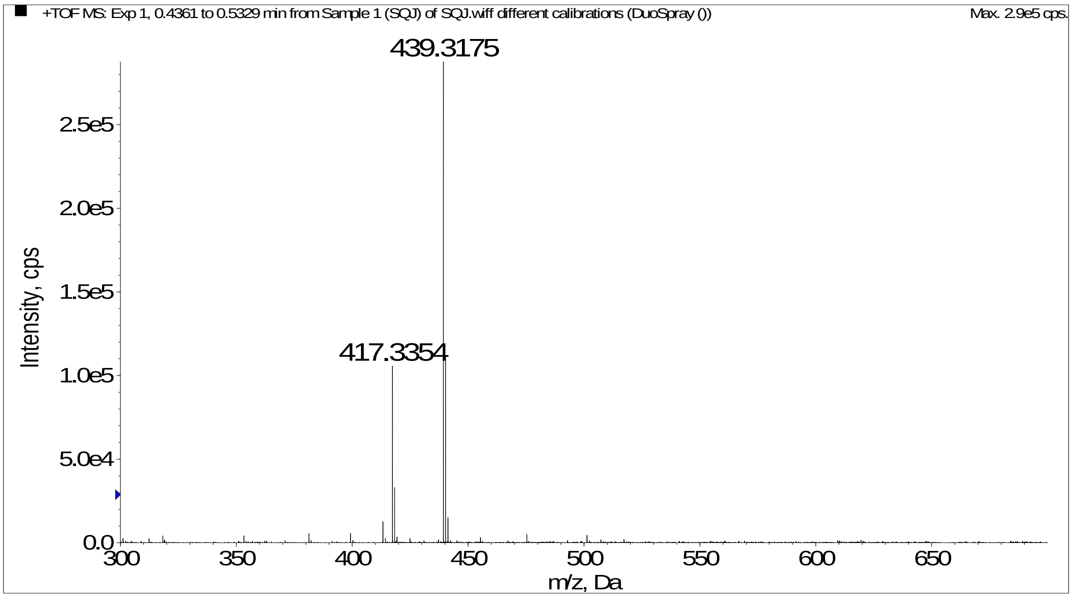

Figure S2. HR-MS of compound **7**

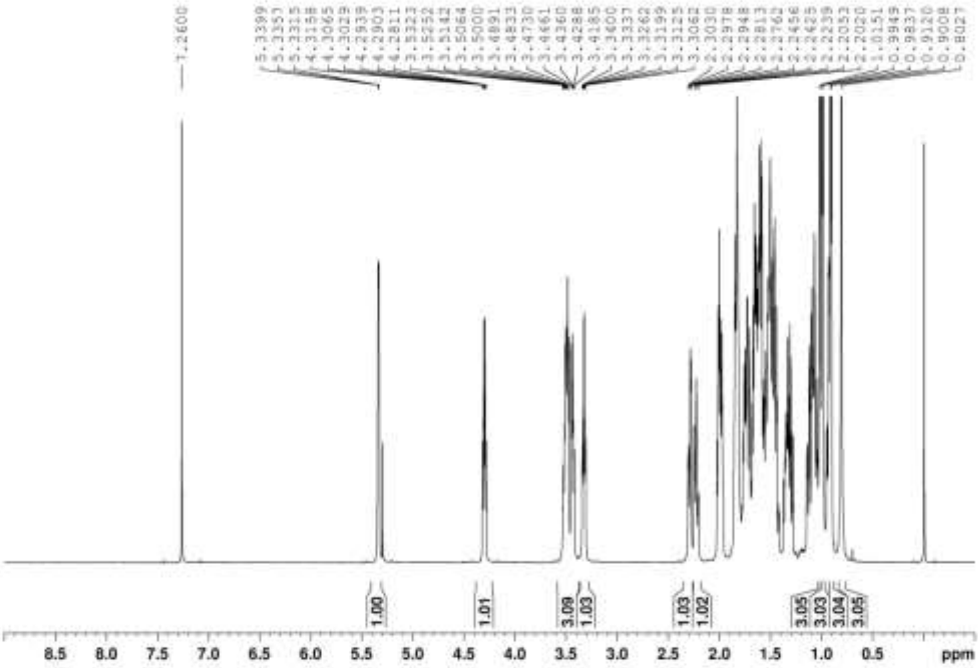

Figure S3. ^{1}H NMR of compound **7**

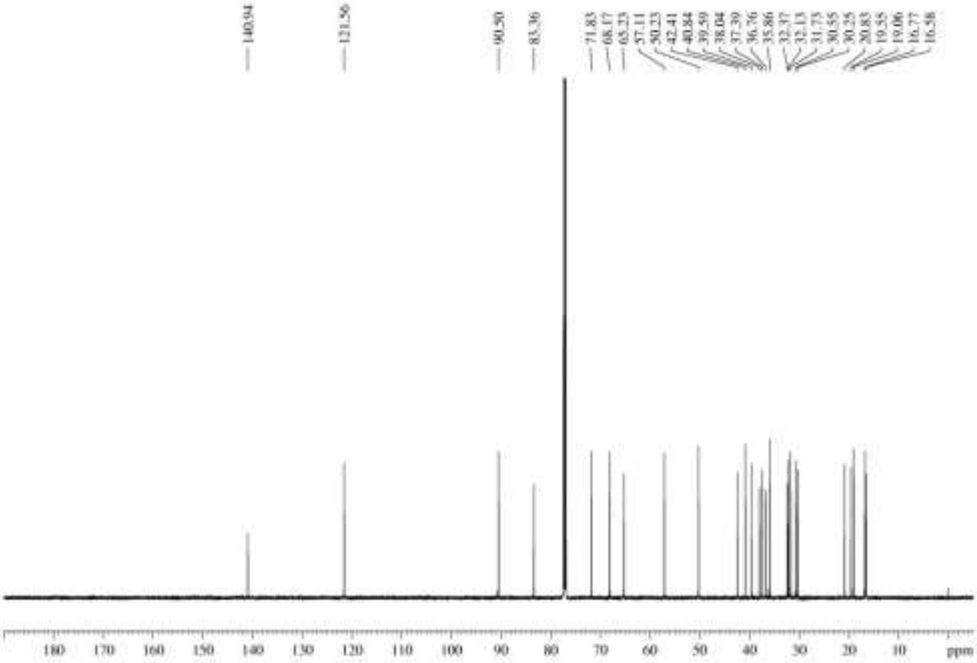

Figure S4. ^{13}C NMR of compound **7**

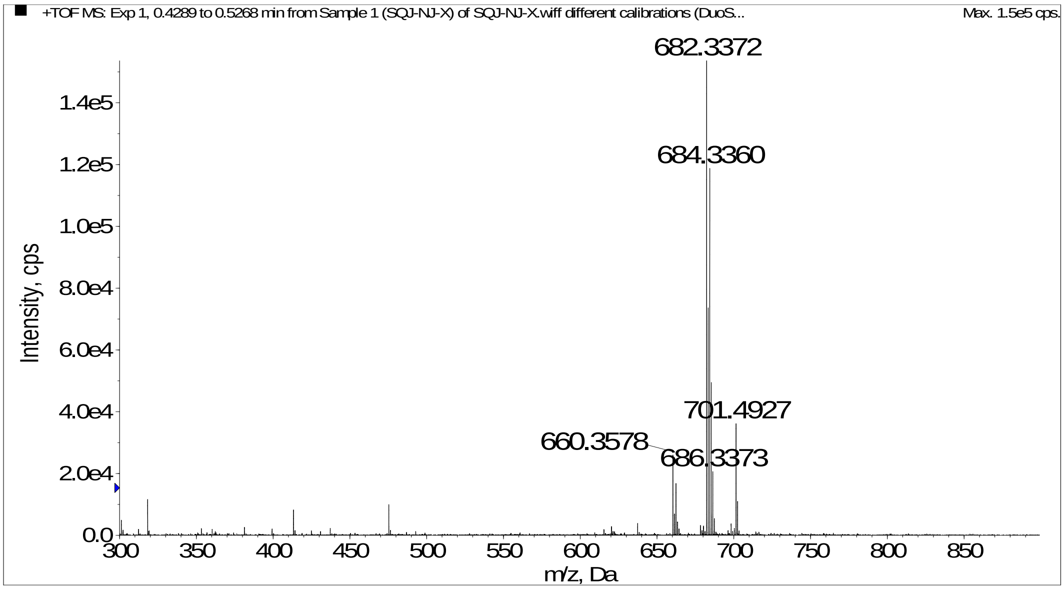

Figure S5. HR-MS of compound **8**

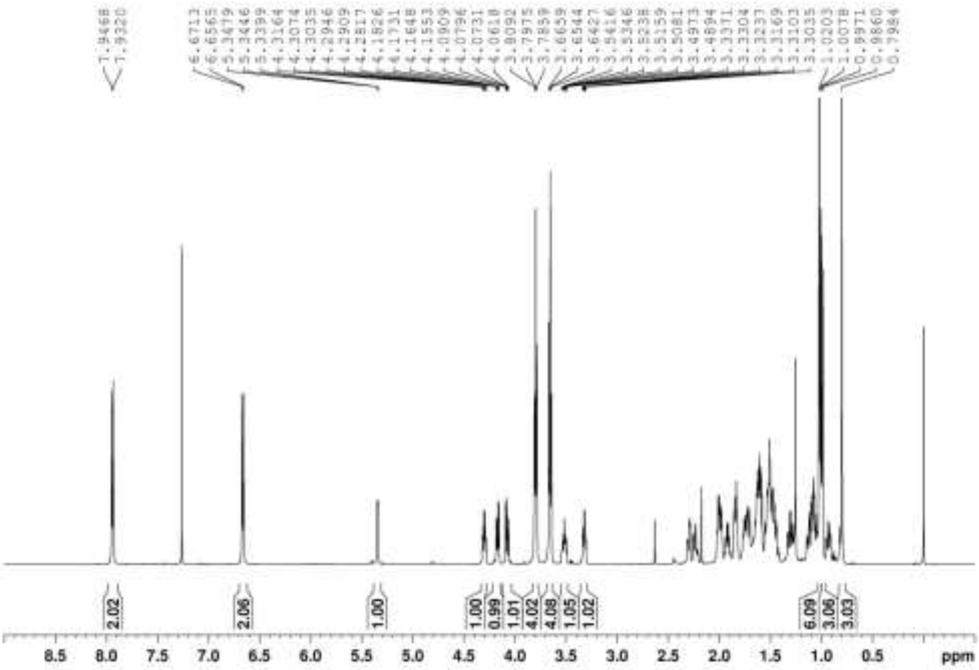

Figure S6. ^{1}H NMR of compound **8**

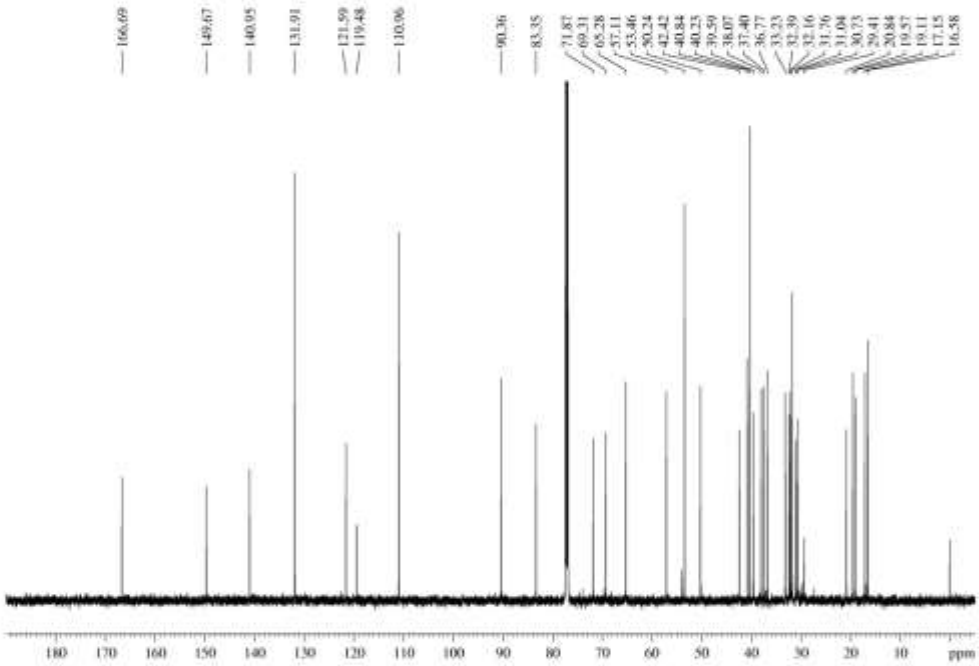

Figure S7. ^{13}C NMR of compound **8**

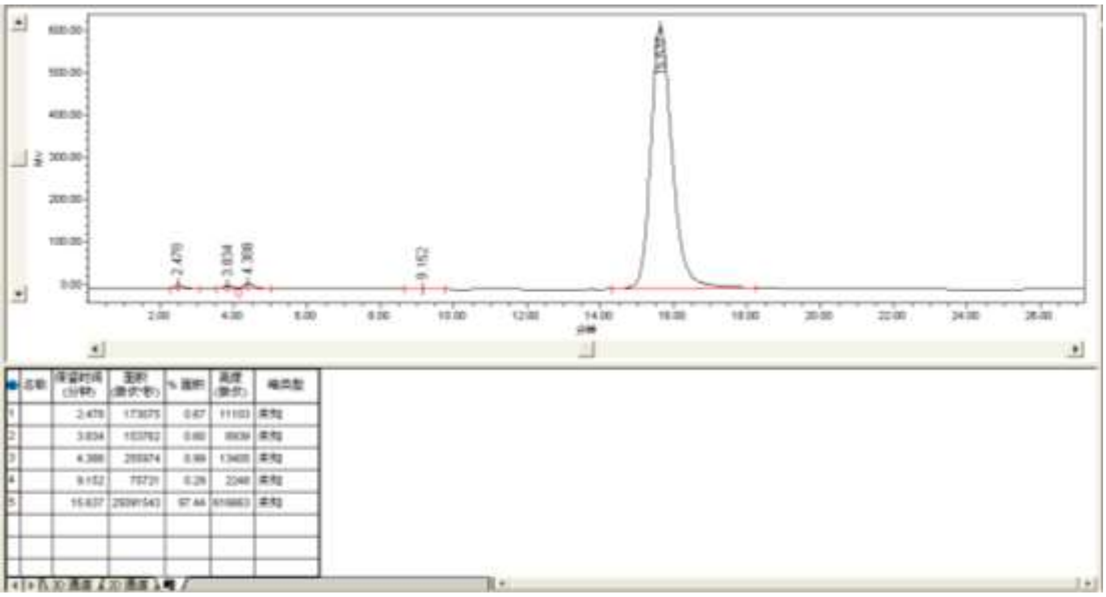

Figure S8. HPLC spectrum of compound **8**

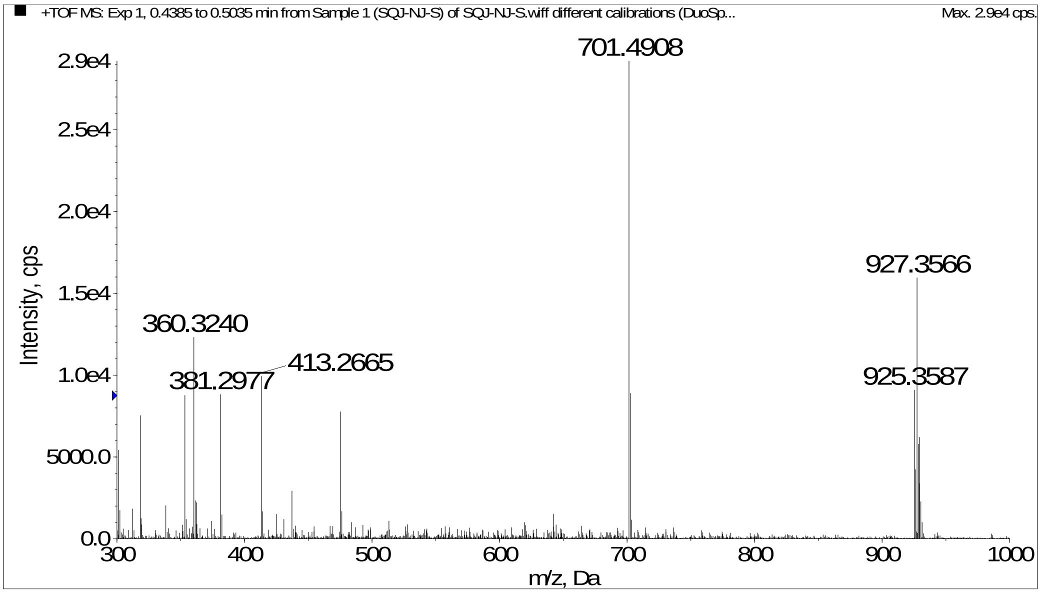

Figure S9. HR-MS of compound **9**

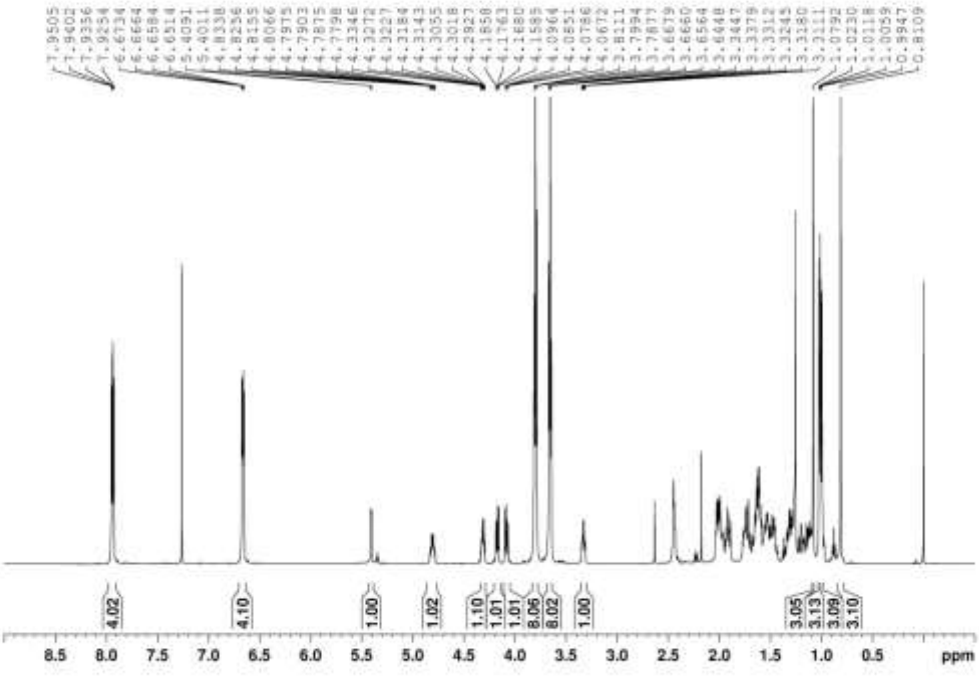

Figure S10. ^{1}H NMR of compound **9**

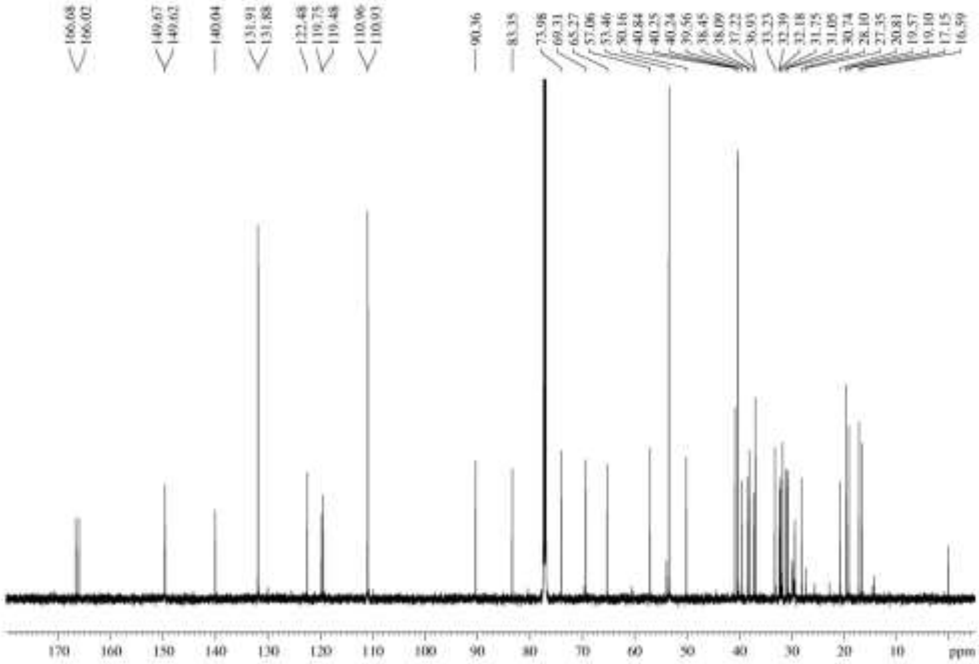

Figure S11. ^{13}C NMR of compound **9**

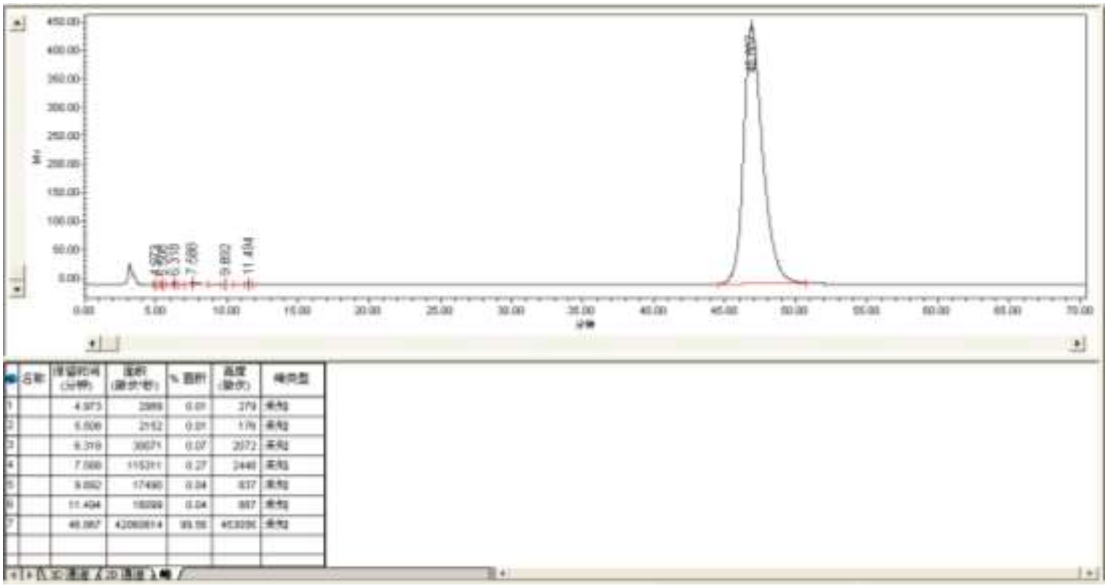

Figure S12. HPLC spectrum of compound **9**

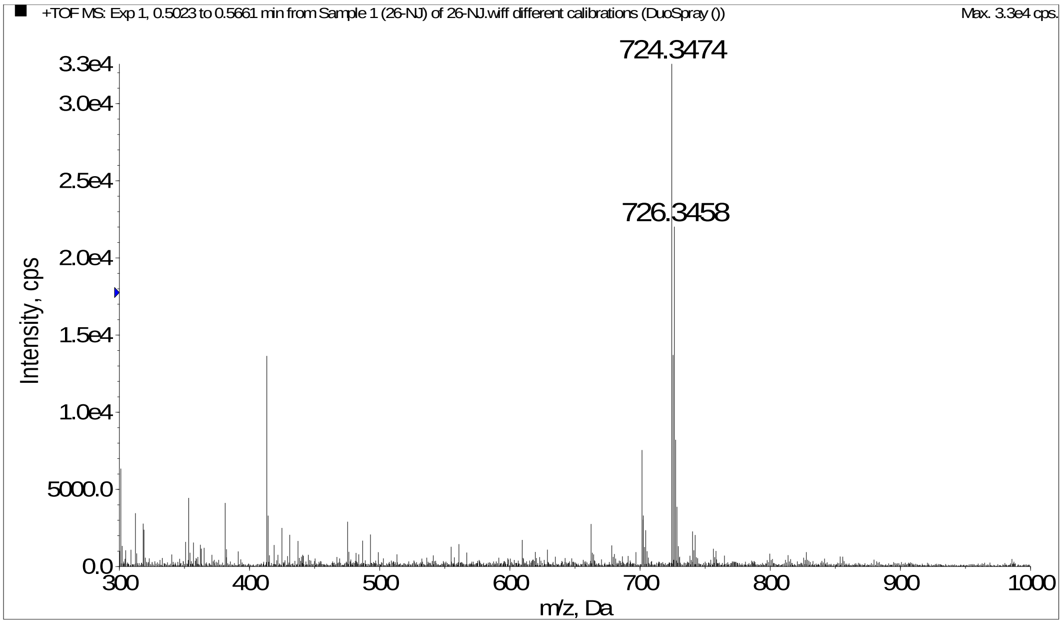

Figure S13. HR-MS of compound **10**

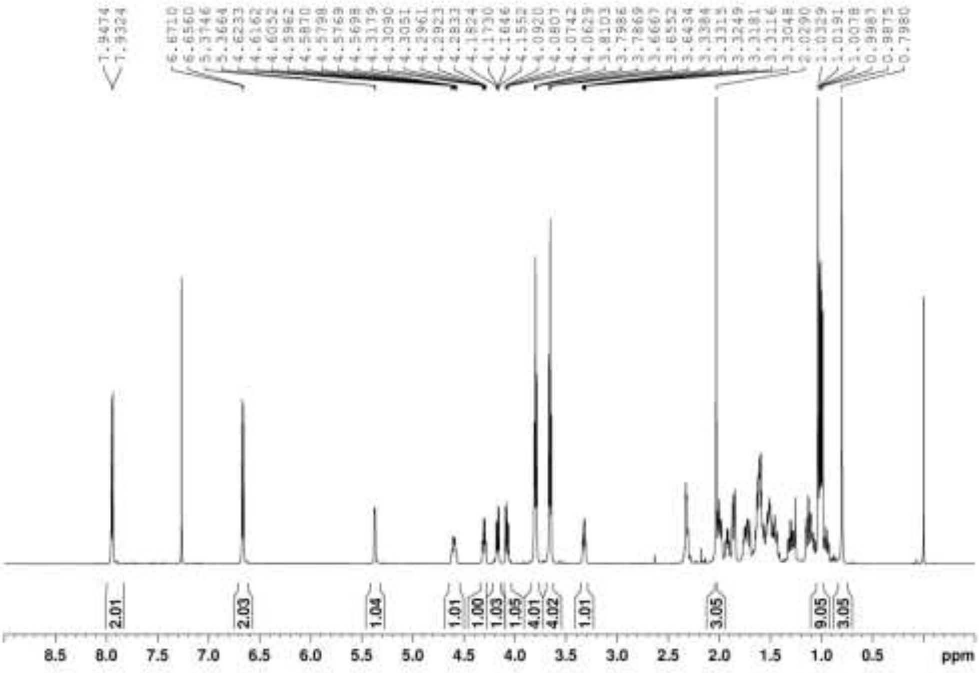

Figure S14. ^{1}H NMR of compound **10**

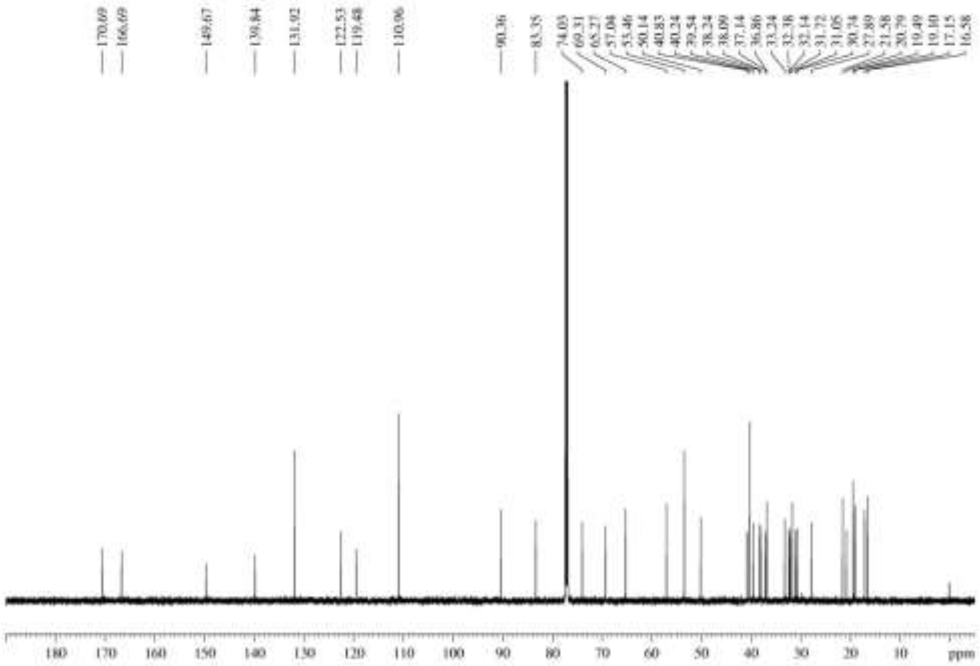

Figure S15. ^{13}C NMR of compound **10**

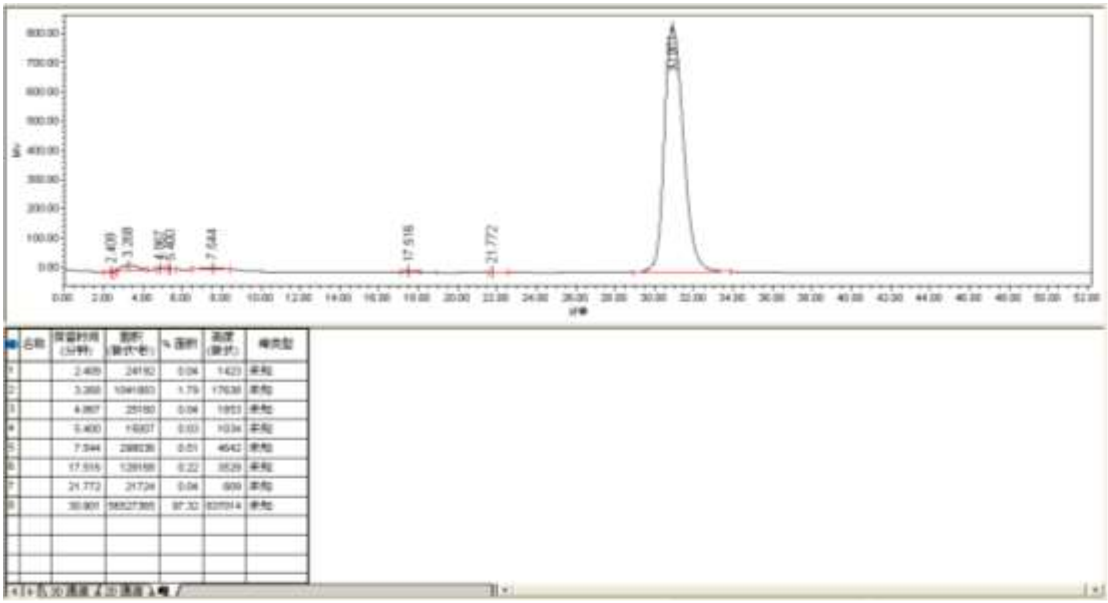

Figure S16. HPLC spectrum of compound **10**

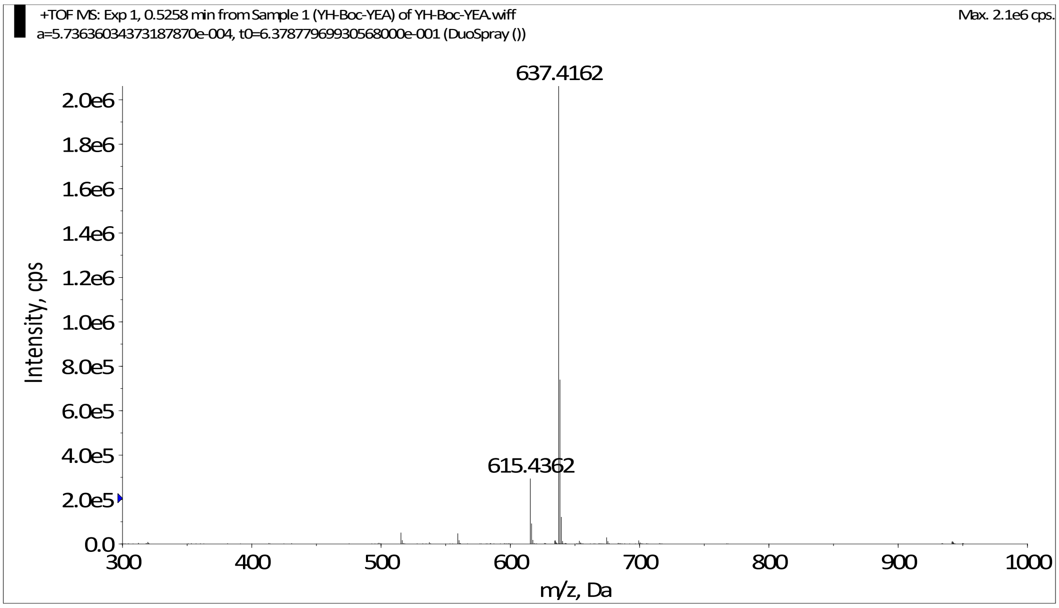

Figure S17. HR-MS of compound **12a**

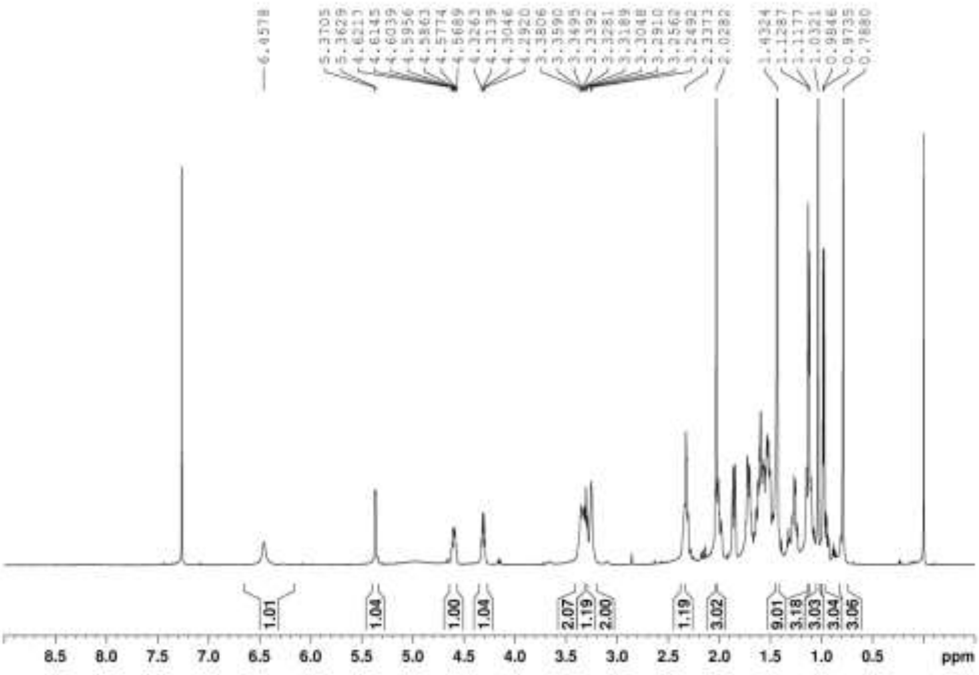

Figure S18. ^{1}H NMR of compound **12a**

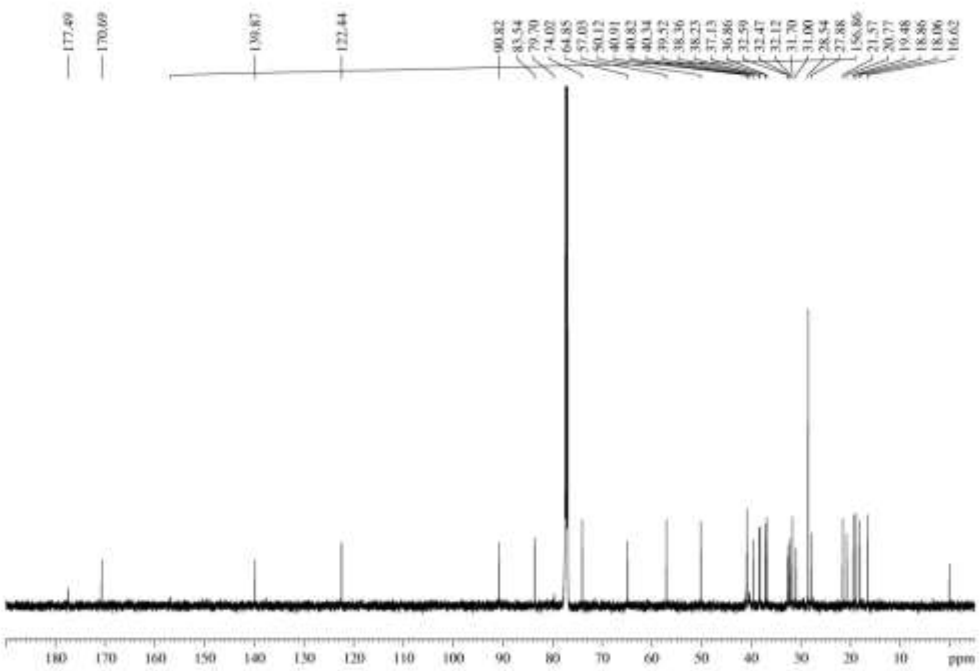

Figure S19. ^{13}C NMR of compound **12a**

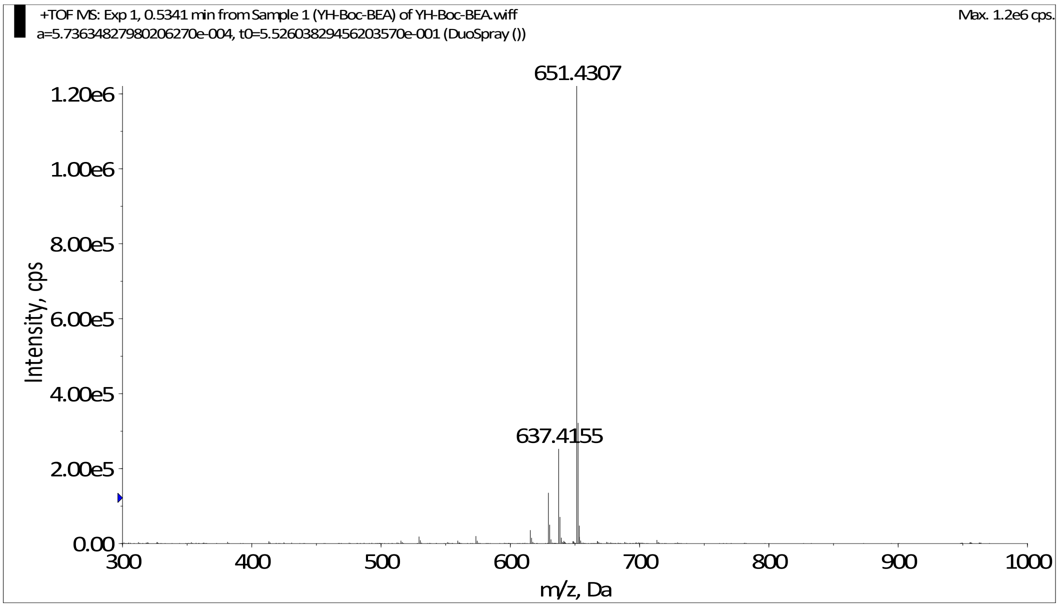

Figure S20. HR-MS of compound **12b**

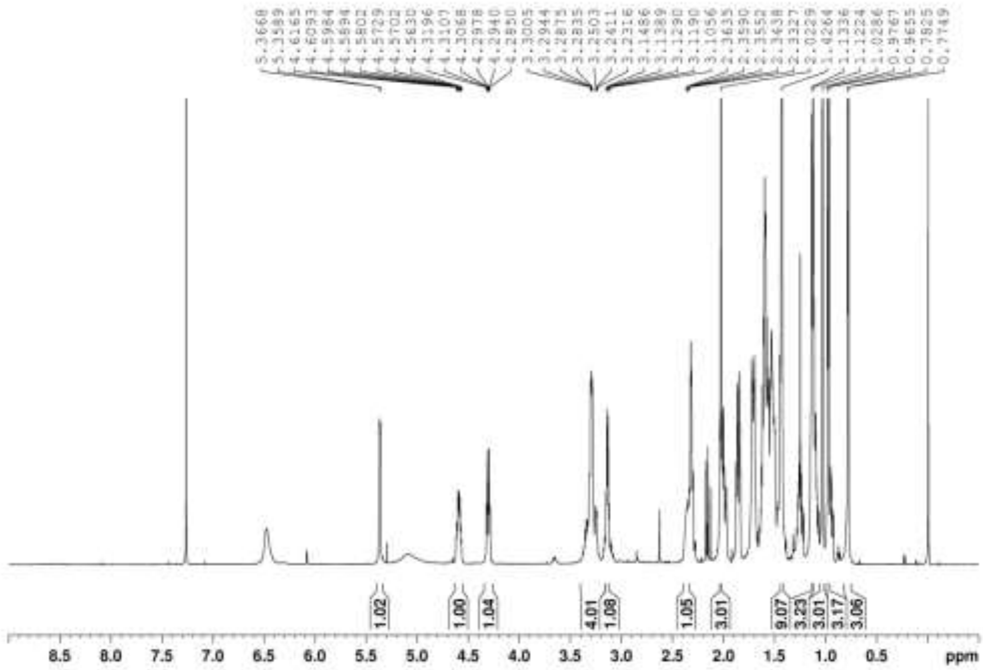

Figure S21. ^{1}H NMR of compound **12b**

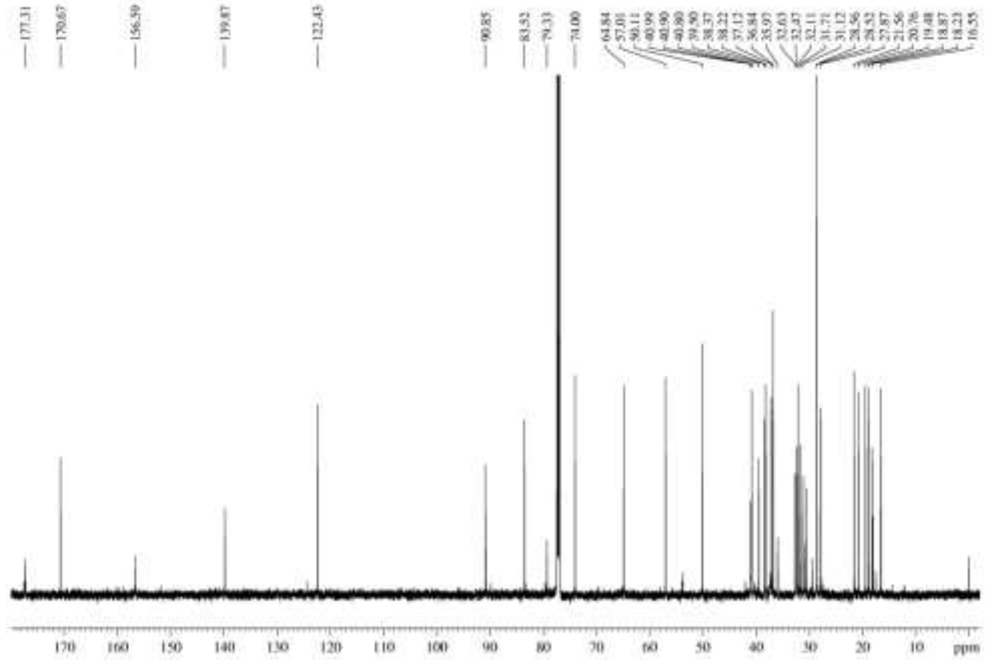

Figure S22. ^{13}C NMR of compound **12b**

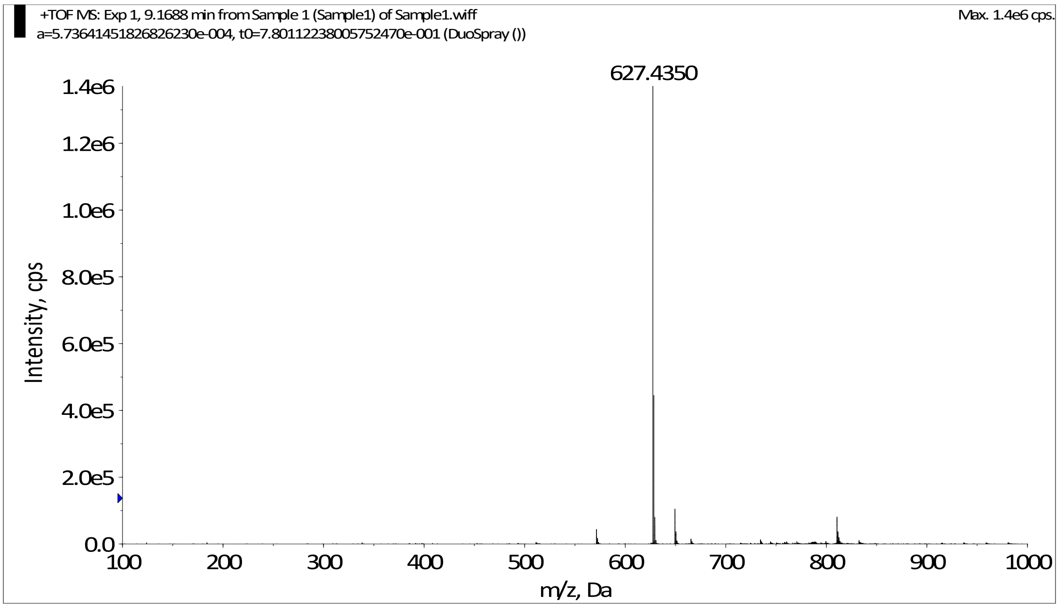

Figure S23. HR-MS of compound **12c**

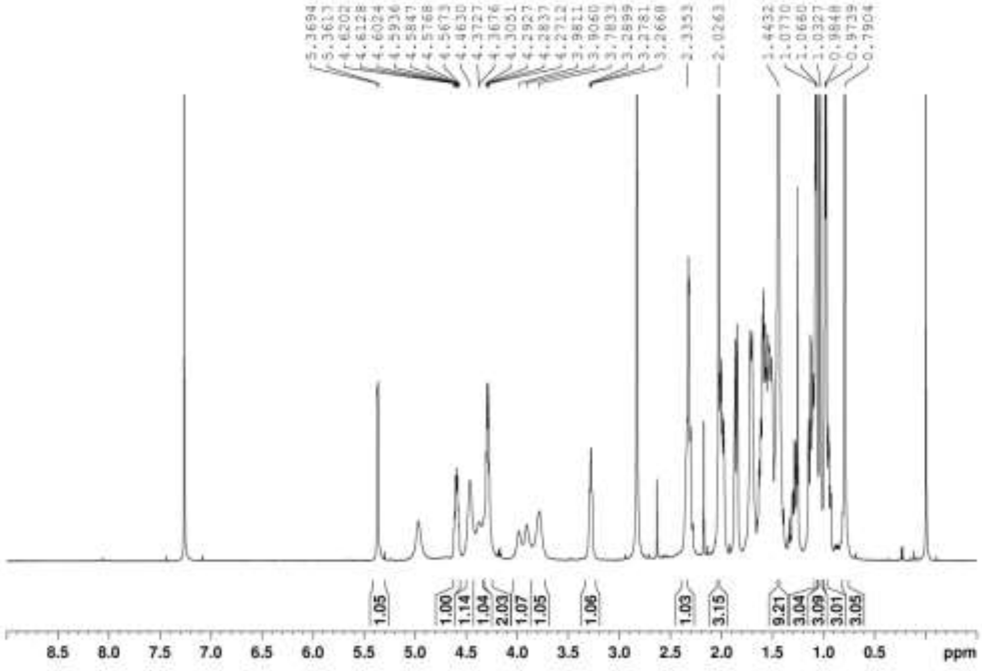

Figure S24. ^{1}H NMR of compound **12c**

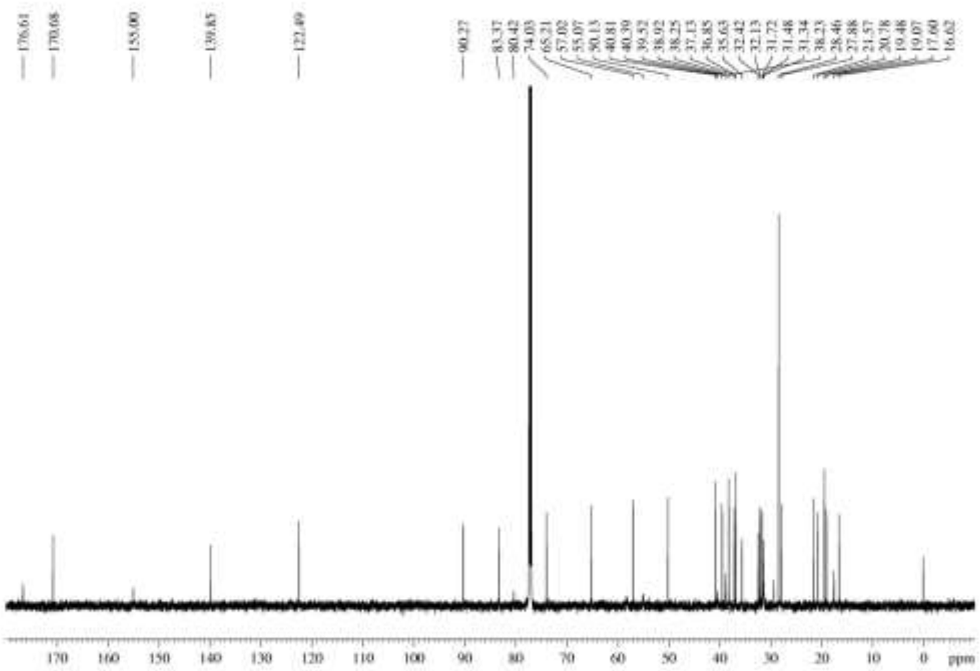

Figure S25. ^{13}C NMR of compound **12c**

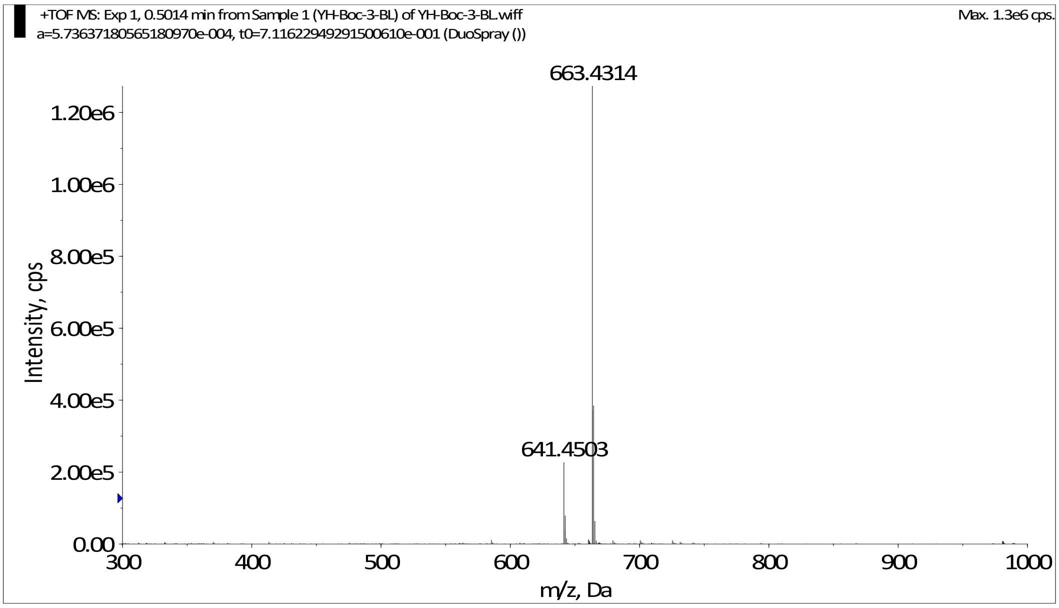

Figure S26. HR-MS of compound **12d**

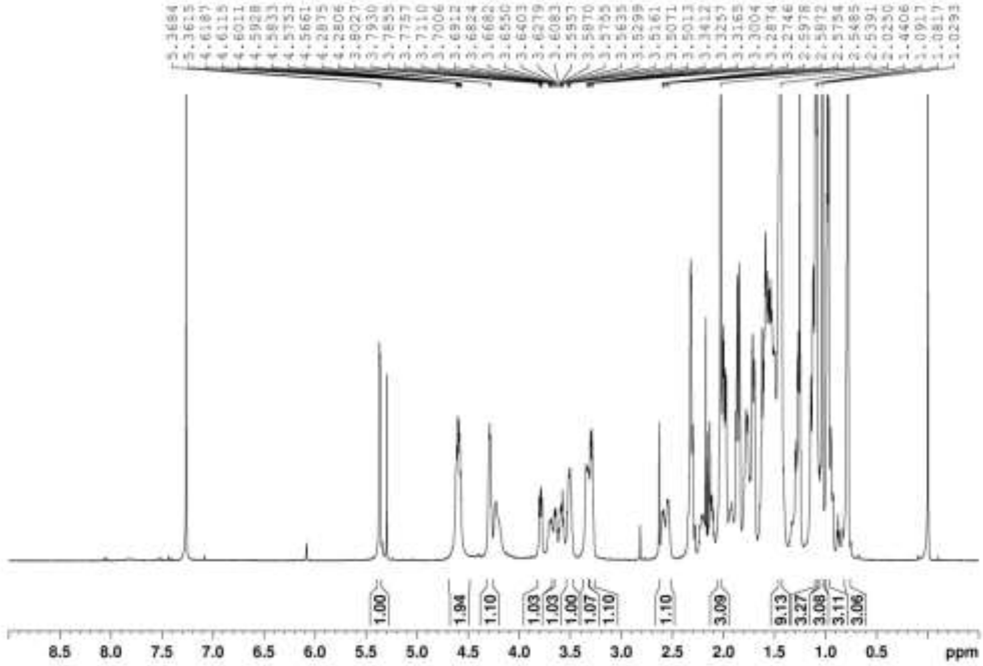

Figure S27. ^{1}H NMR of compound **12d**

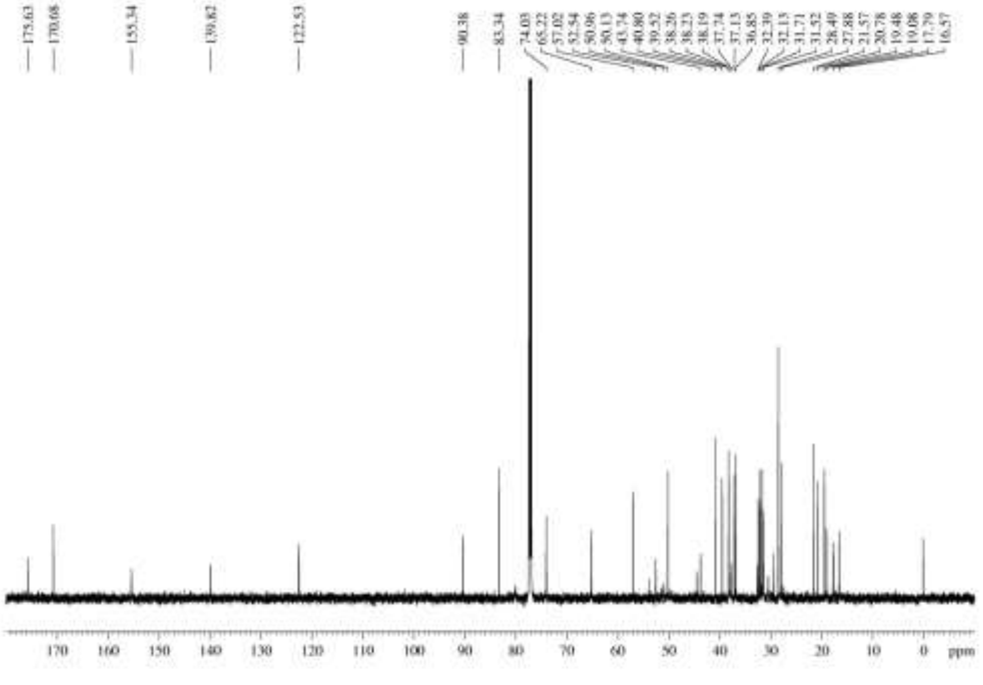

Figure S28. ^{13}C NMR of compound **12d**

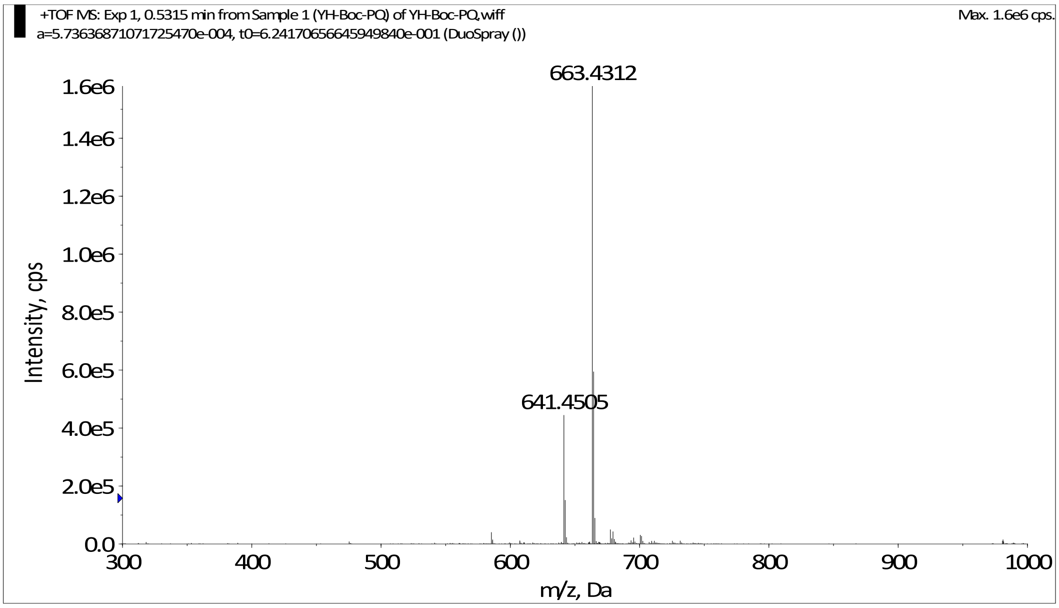

Figure S29. HR-MS of compound **12e**

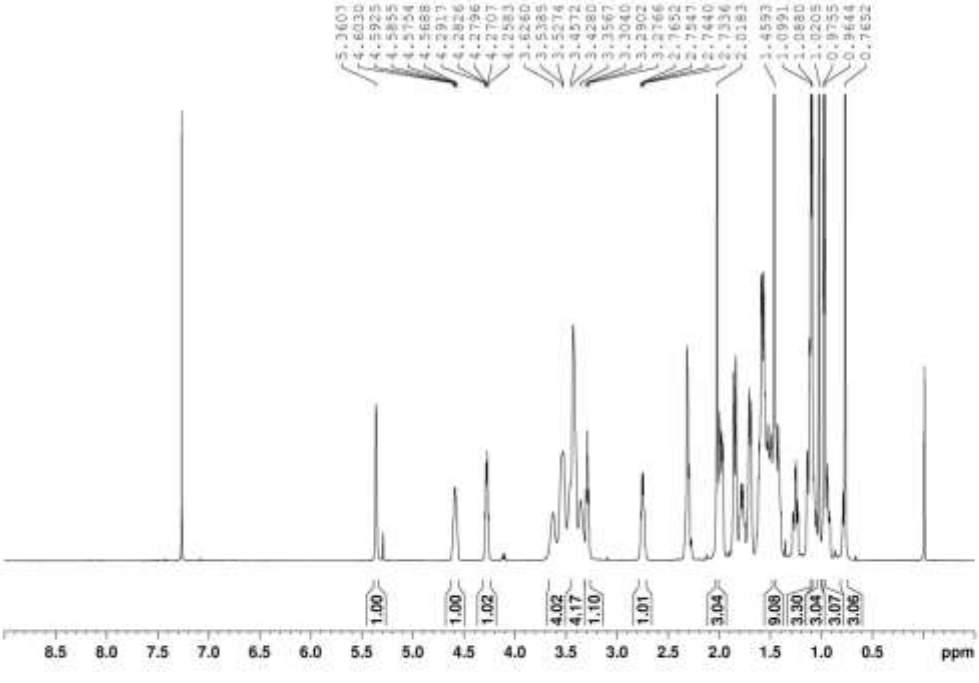

Figure S30. ^{1}H NMR of compound **12e**

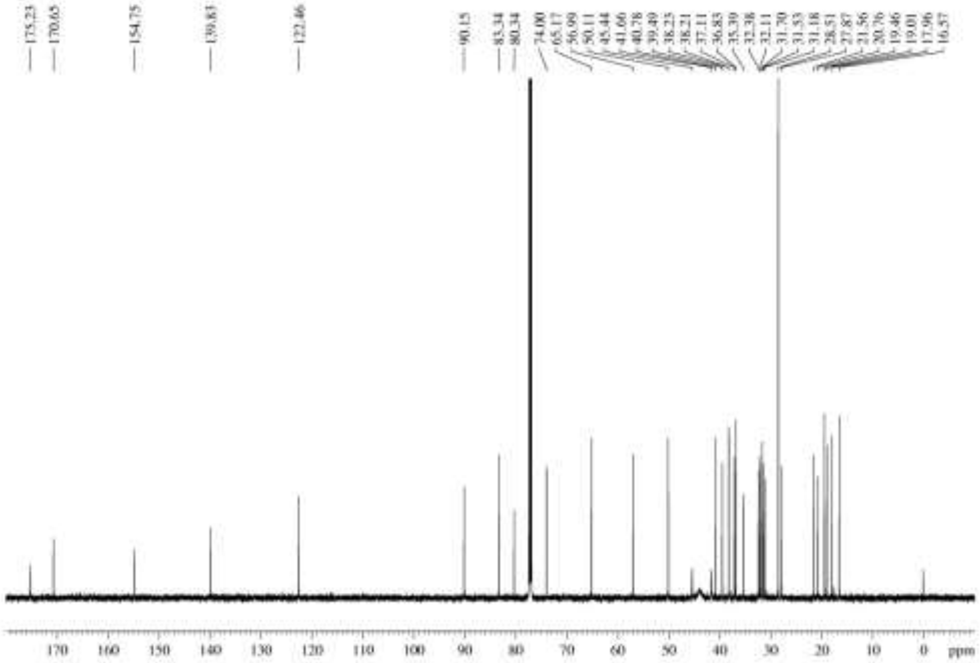

Figure S31. ^{13}C NMR of compound **12e**

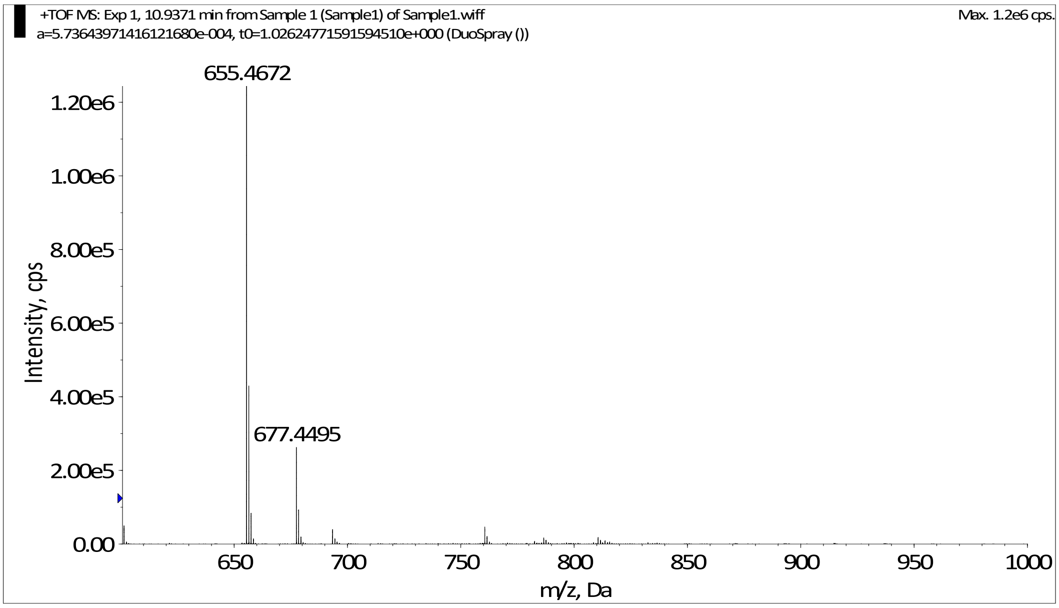

Figure S32. HR-MS of compound **12f**

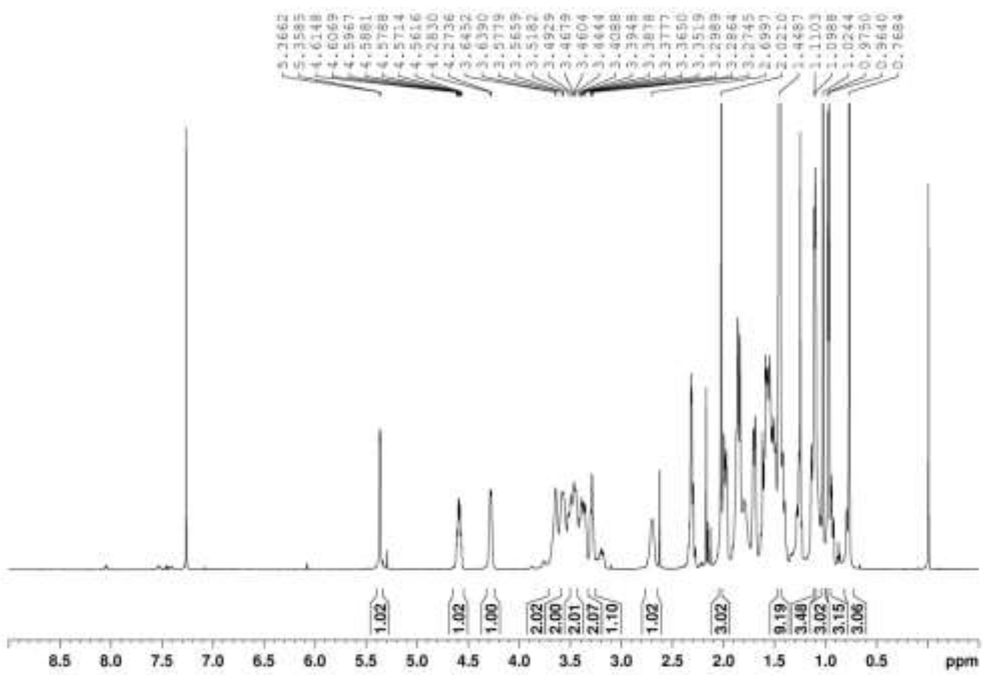

Figure S33. ^{1}H NMR of compound **12f**

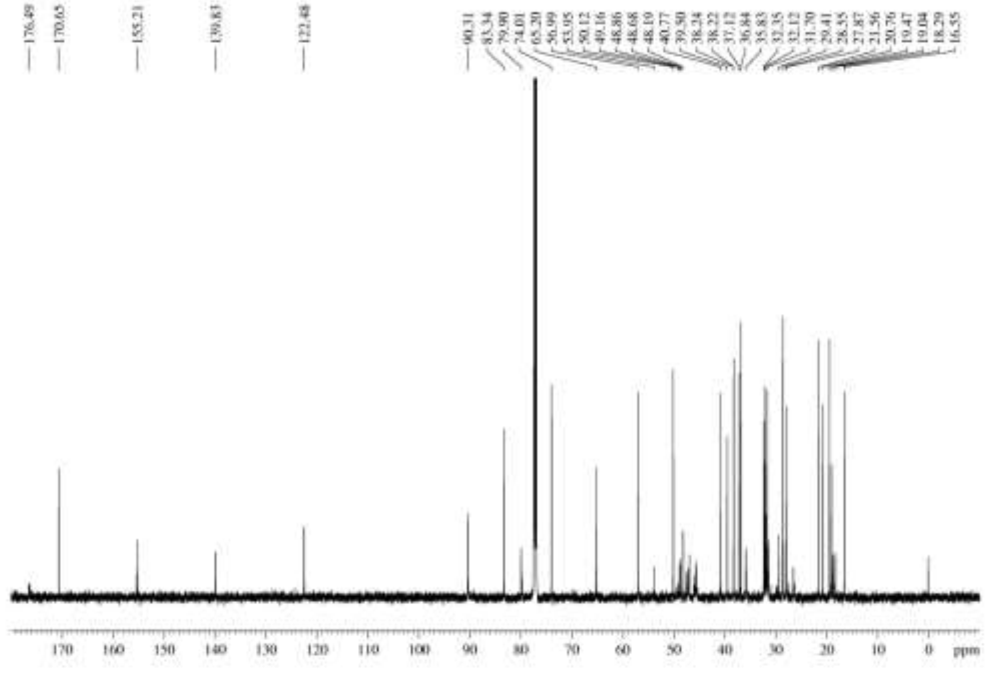

Figure S34. ^{13}C NMR of compound **12f**

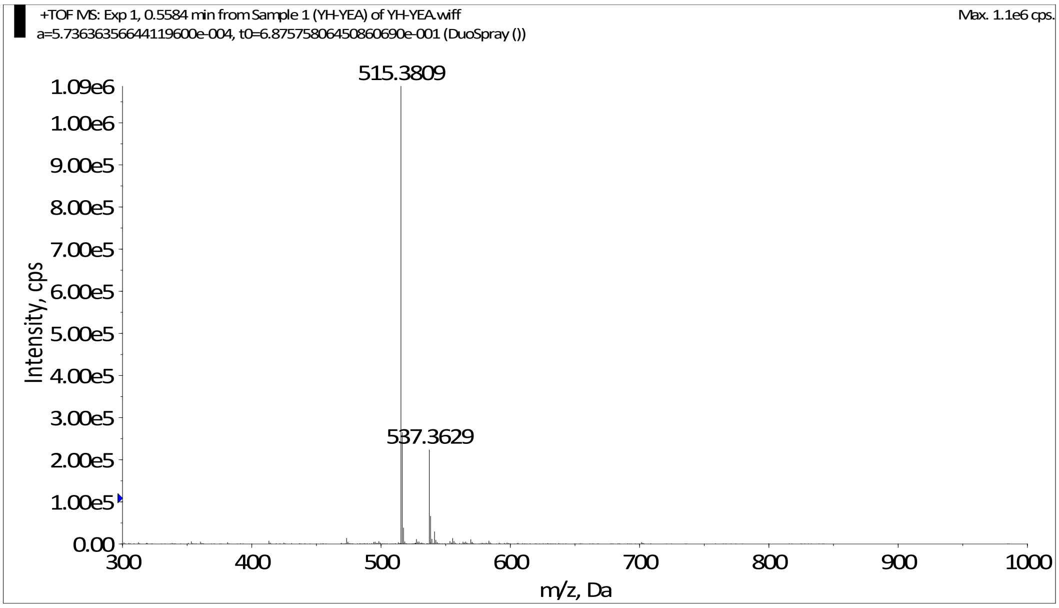

Figure S35. HR-MS of compound **13a**

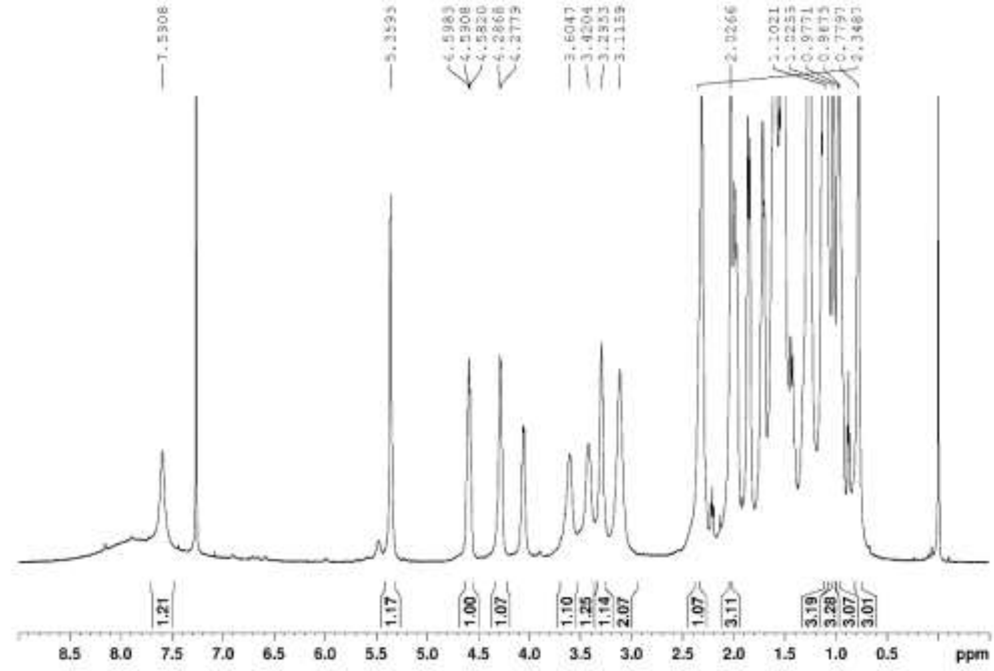

Figure S36. ^{1}H NMR of compound **13a**

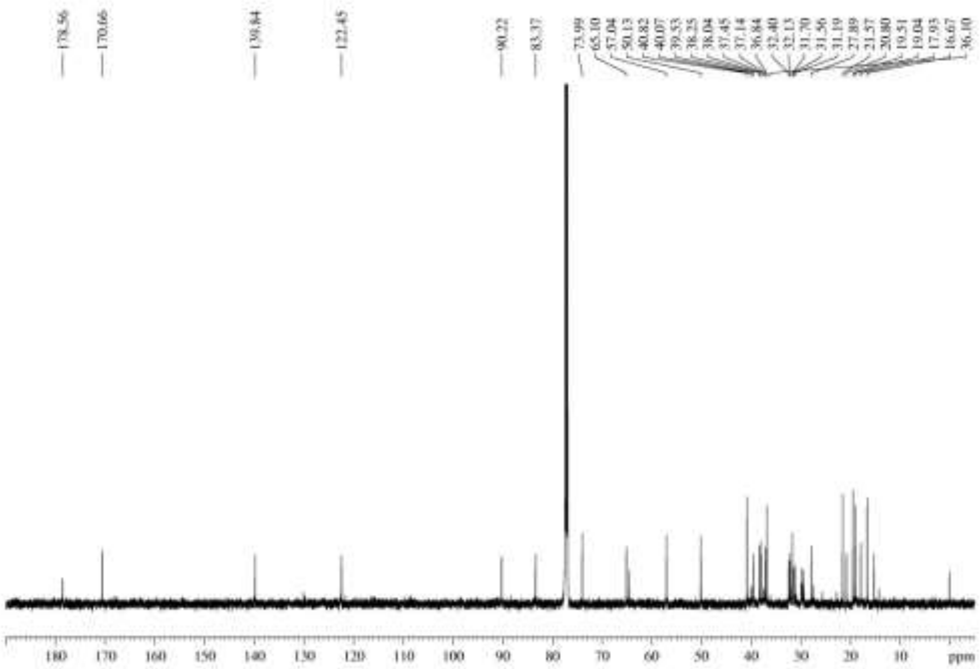

Figure S37. ^{13}C NMR of compound **13a**

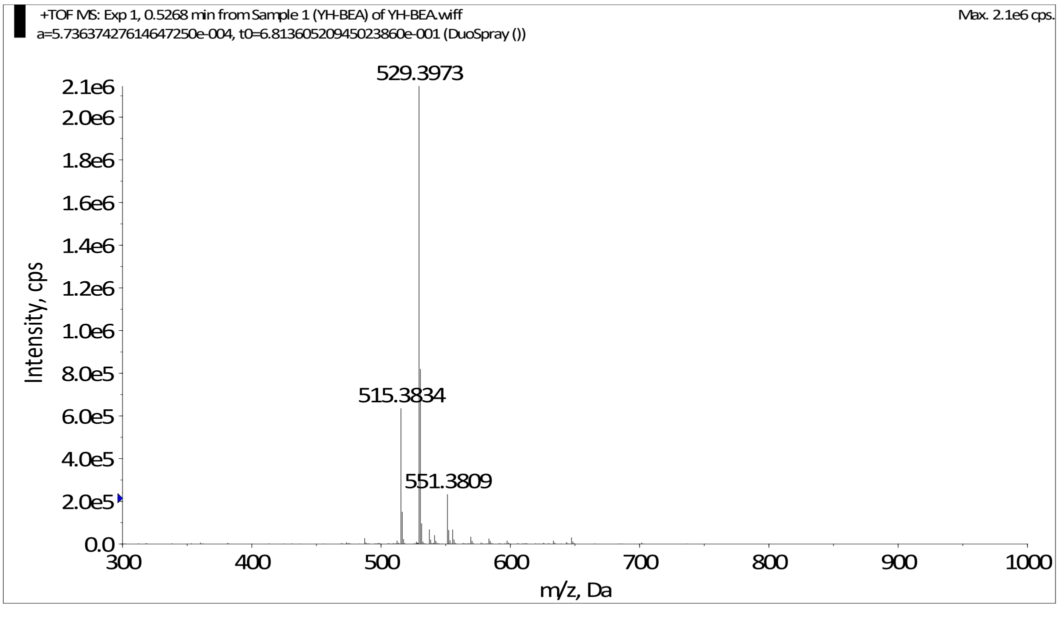

Figure S38. HR-MS of compound **13b**

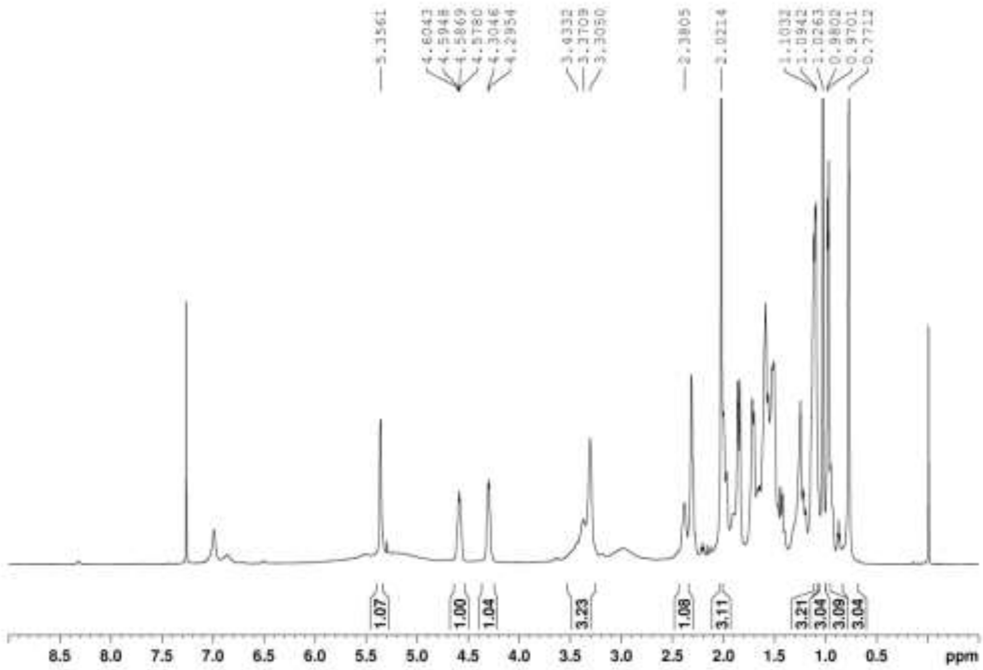

Figure S39. ^{1}H NMR of compound **13b**

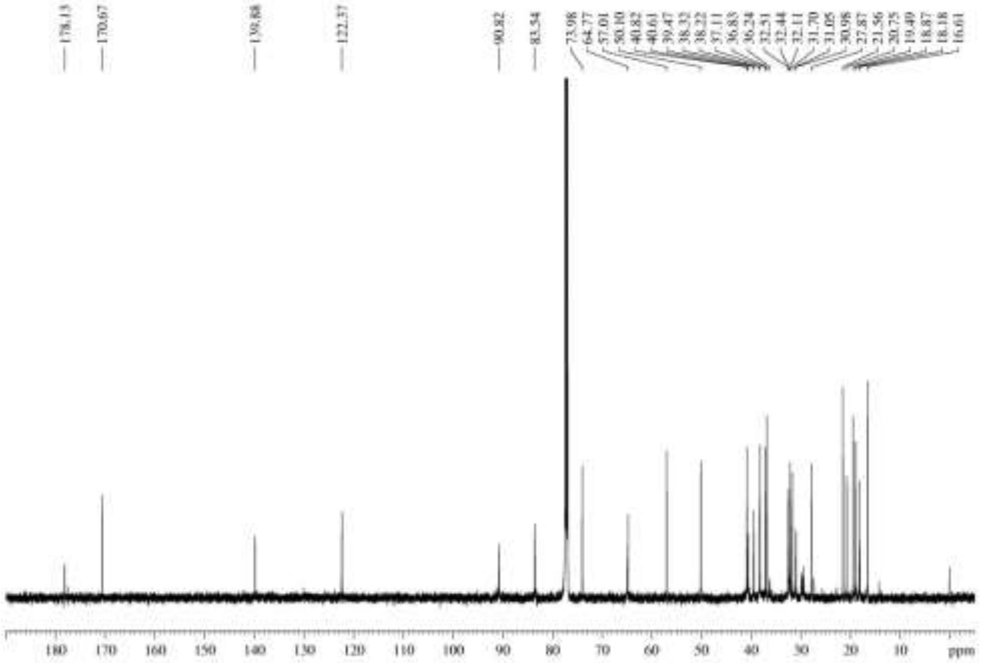

Figure S40. ^{13}C NMR of compound **13b**

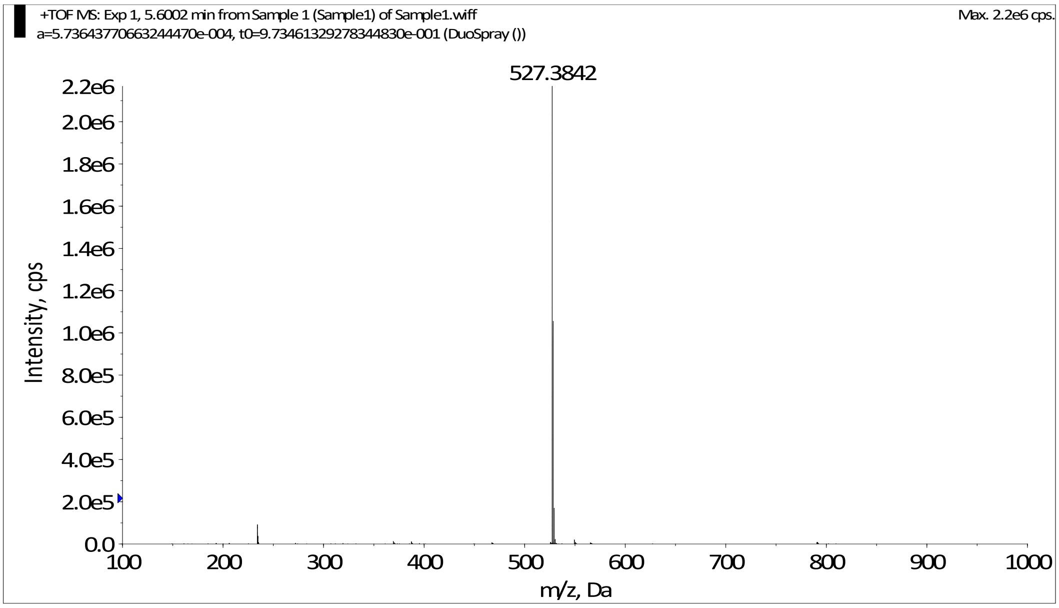

Figure S41. HR-MS of compound **13c**

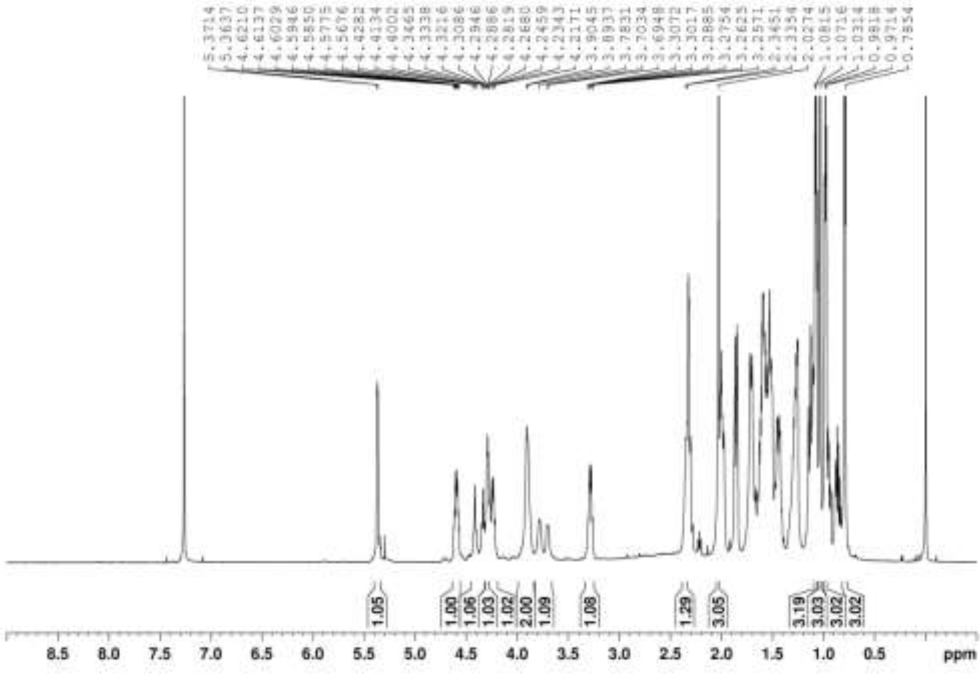

Figure S42. ^{1}H NMR of compound **13c**

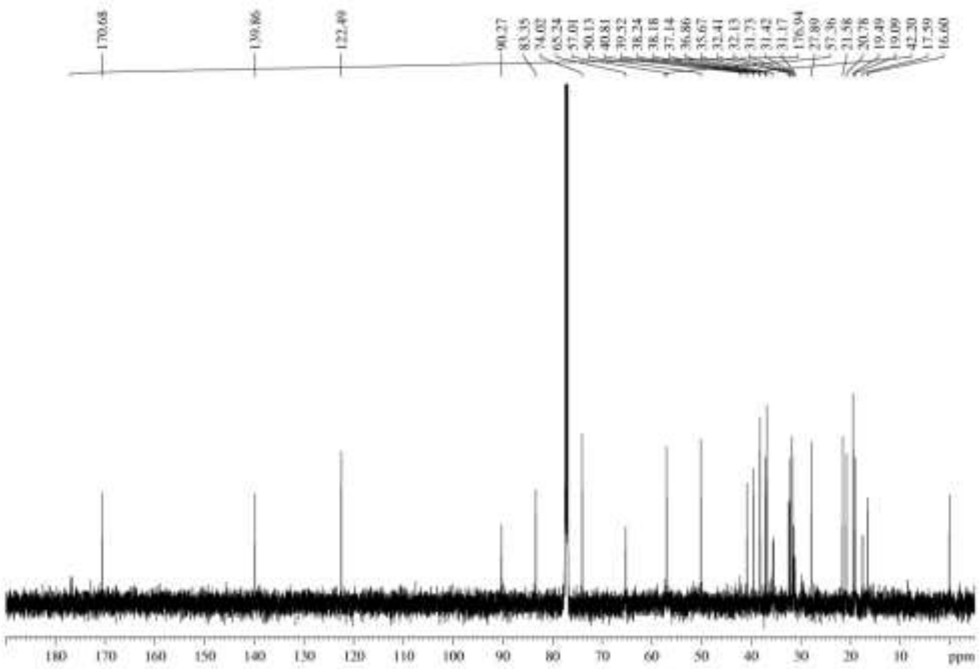

Figure S43. ^{13}C NMR of compound **13c**

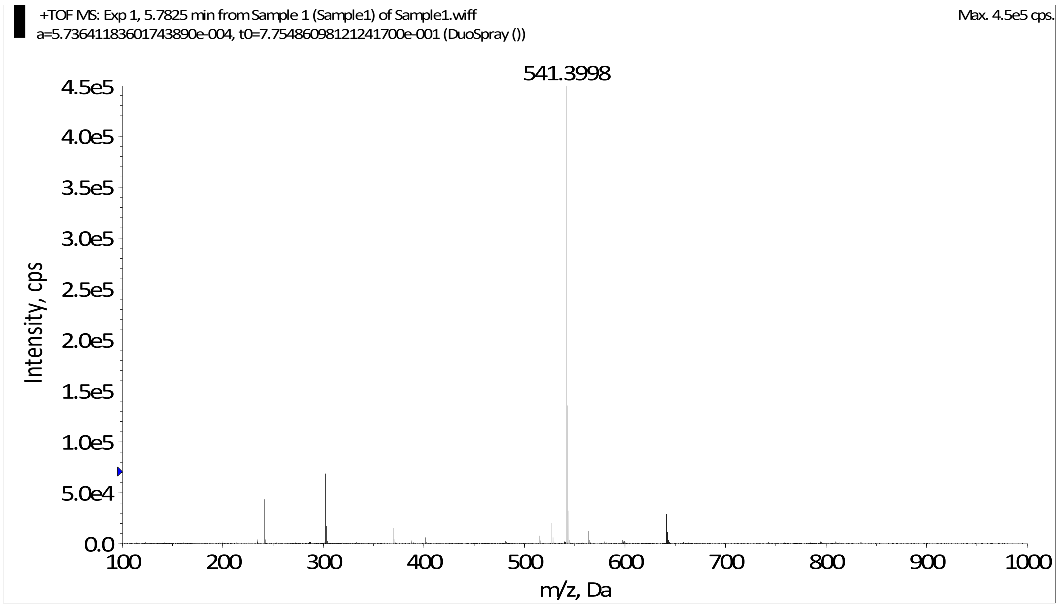

Figure S44. HR-MS of compound **13d**

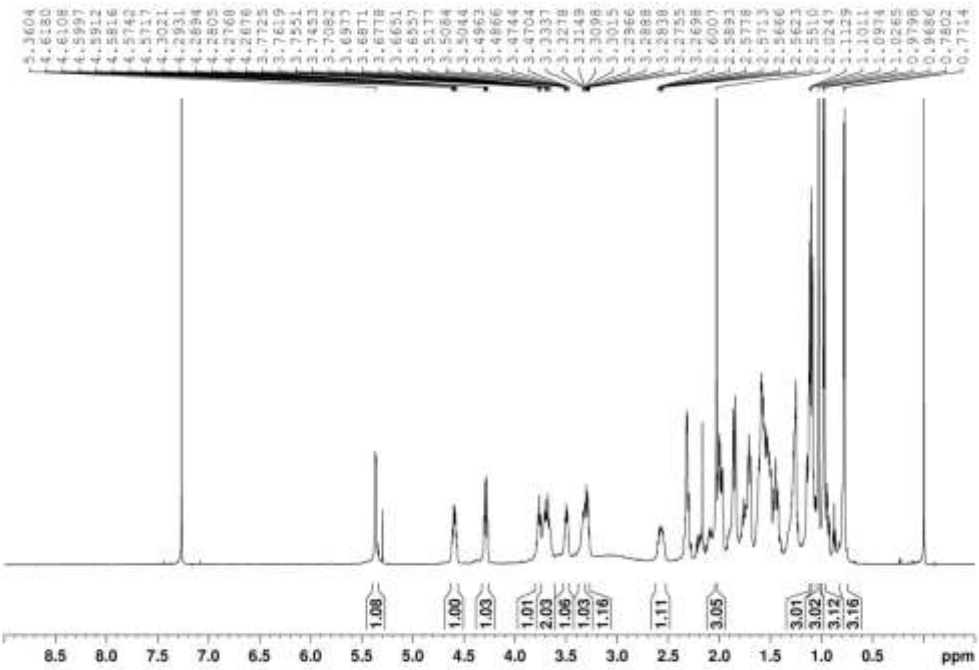

Figure S45. ¹H NMR of compound **13d**

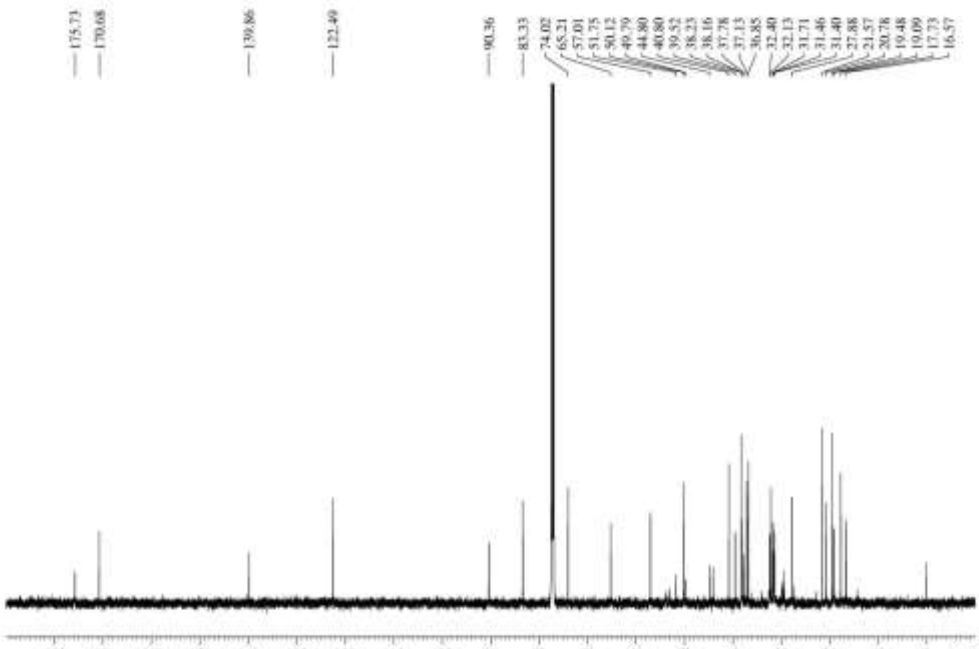

Figure S46. ¹³C NMR of compound **13d**

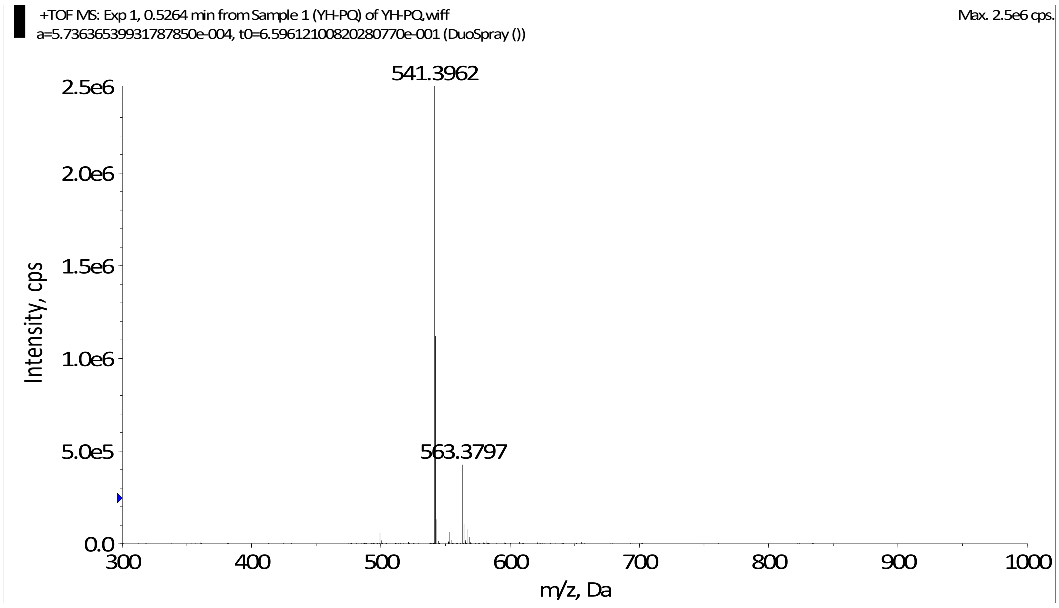

Figure S47. HR-MS of compound **13e**

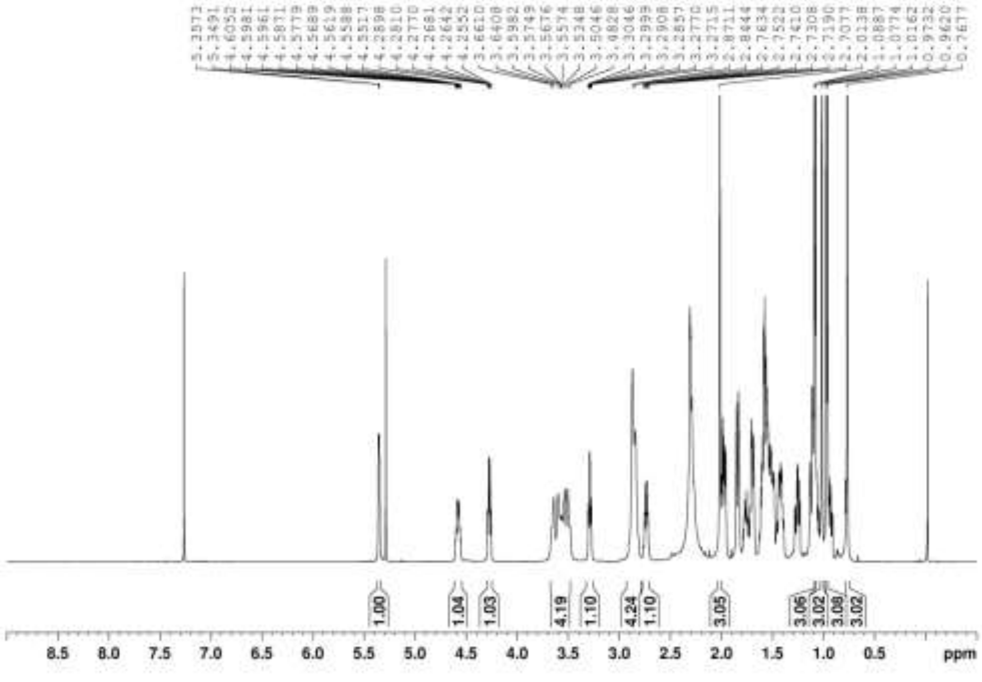

Figure S48. ^{1}H NMR of compound **13e**

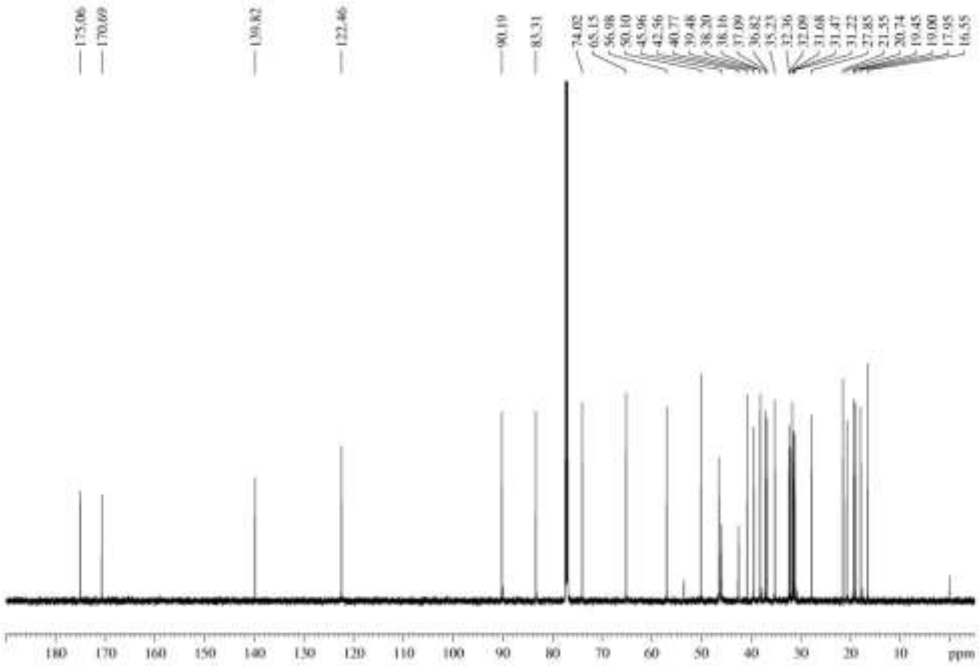

Figure S49. ^{13}C NMR of compound **13e**

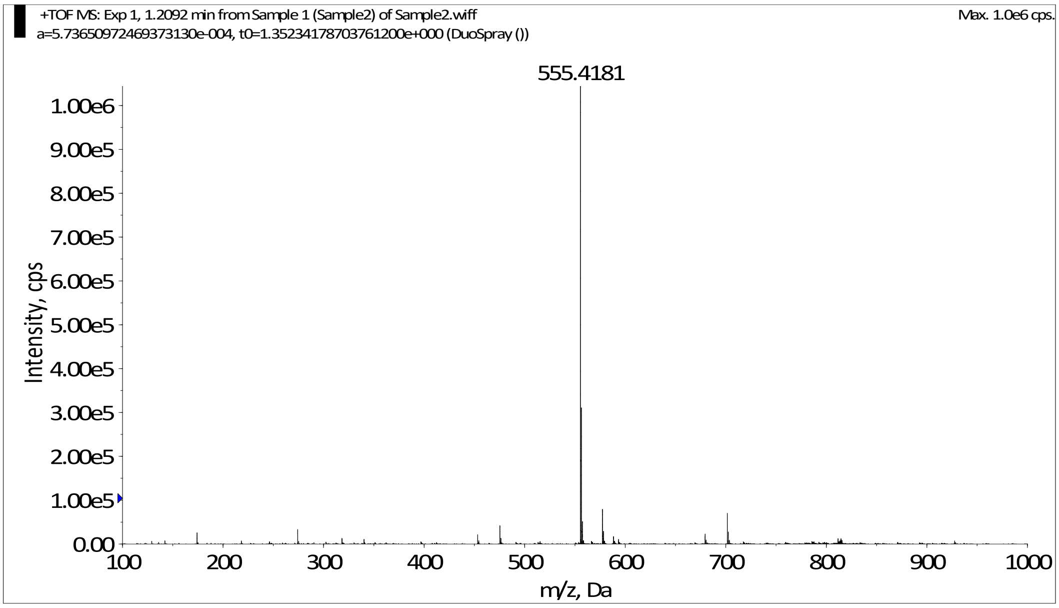

Figure S50. HR-MS of compound **13f**

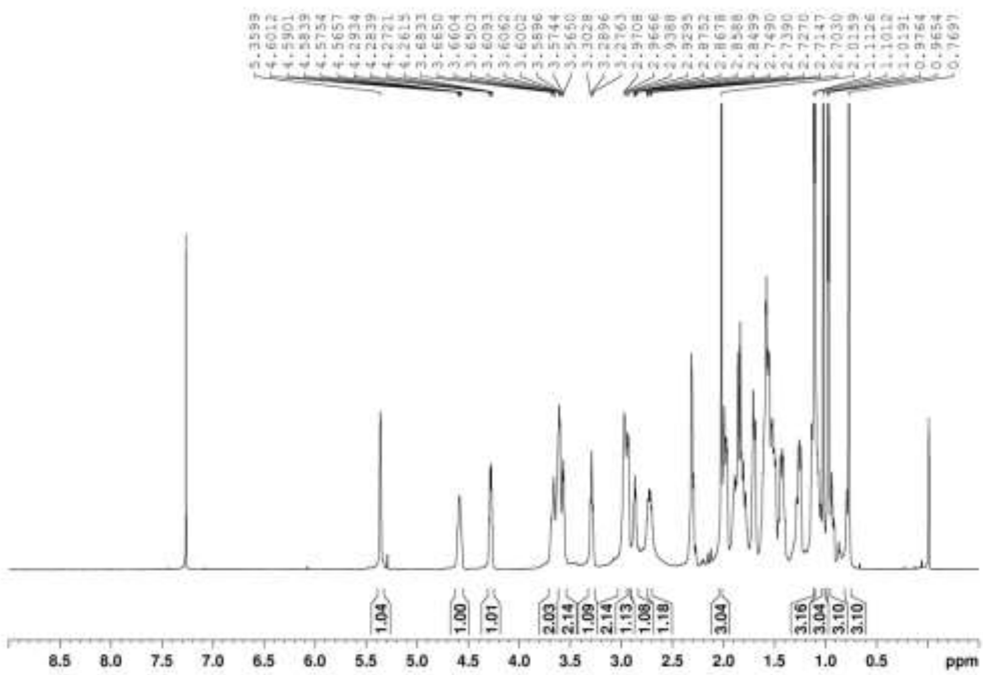

Figure S51. ^{1}H NMR of compound **13f**

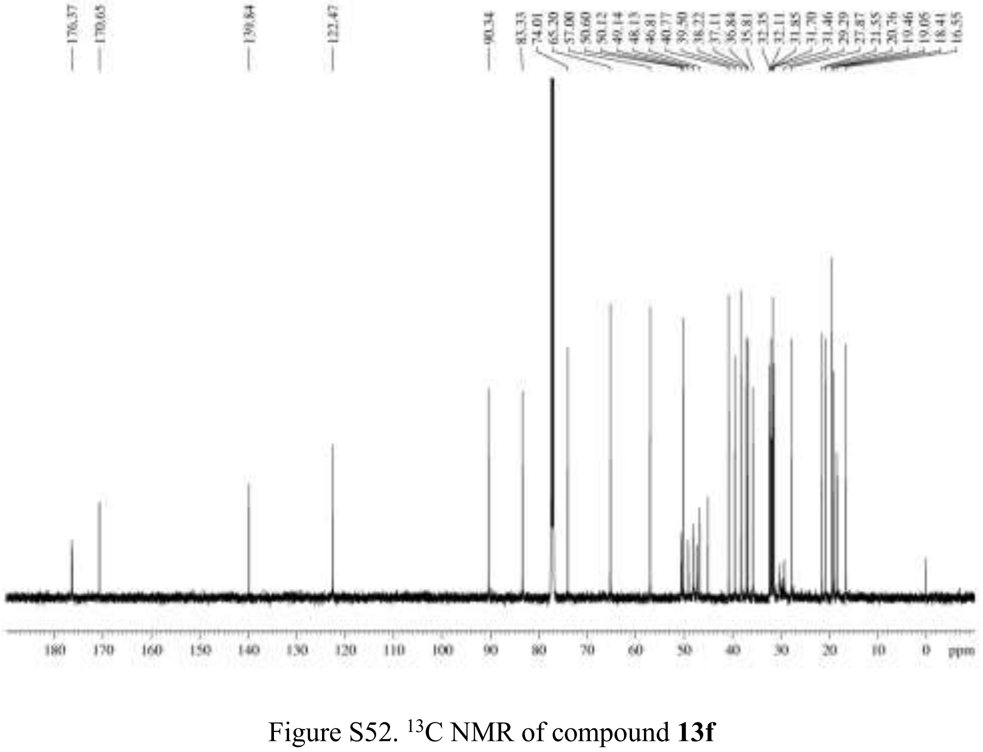

Figure S52. ^{13}C NMR of compound **13f**

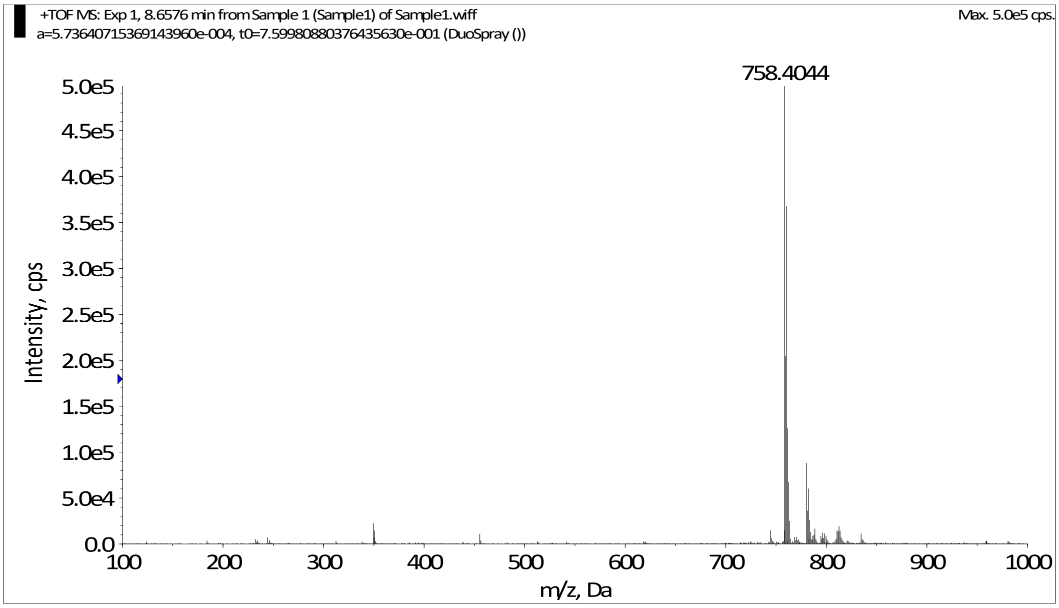

Figure S53. HR-MS of compound **14a**

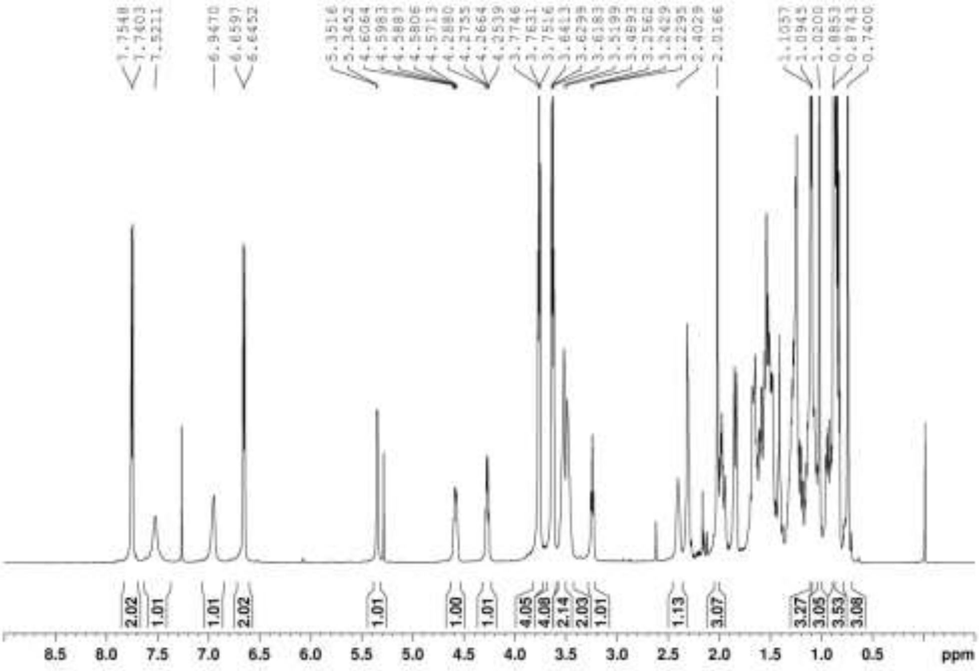

Figure S54. ^{1}H NMR of compound **14a**

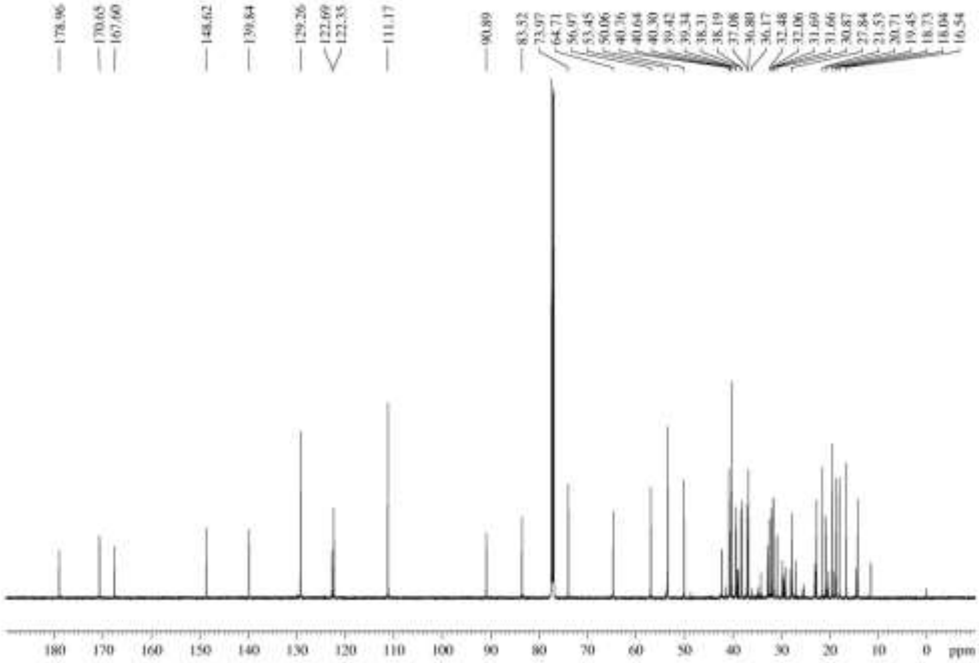

Figure S55. ^{13}C NMR of compound **14a**

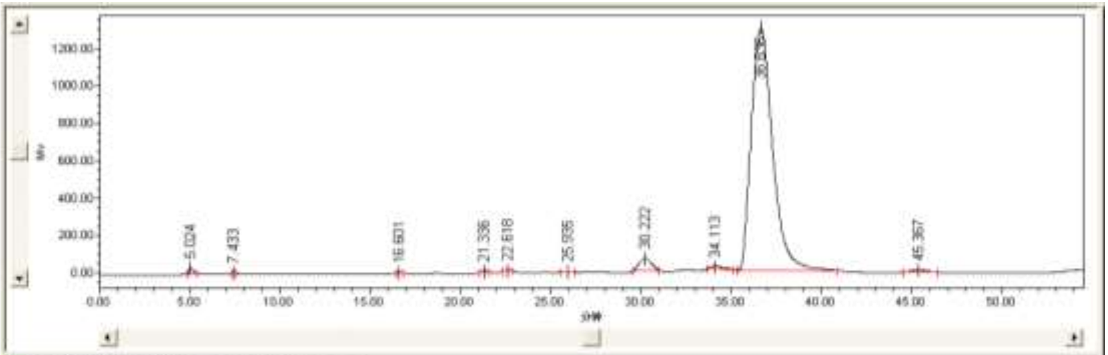

	名称	保留时间 (分钟)	面积 (微伏*秒)	% 面积	高度 (微伏)	峰类型
1		5.024	500609	0.45	30920	未知
2		7.433	37725	0.03	3950	未知
3		16.601	48790	0.04	3173	未知
4		21.336	98682	0.09	5119	未知
5		22.618	114186	0.10	5535	未知
6		25.935	83405	0.07	3093	未知
7		30.222	3004862	2.69	65609	未知
8		34.113	690047	0.62	13025	未知
9		36.636	106276143	95.16	1295927	未知
10		45.367	826235	0.74	13206	未知

Figure S56. HPLC spectrum of compound **14a**

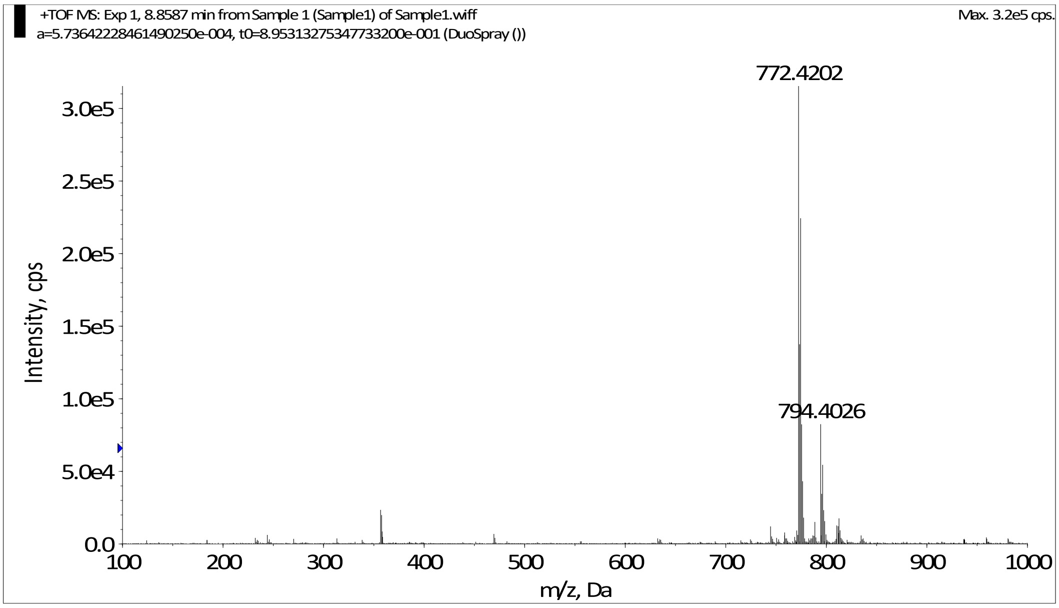

Figure S57. HR-MS of compound **14b**

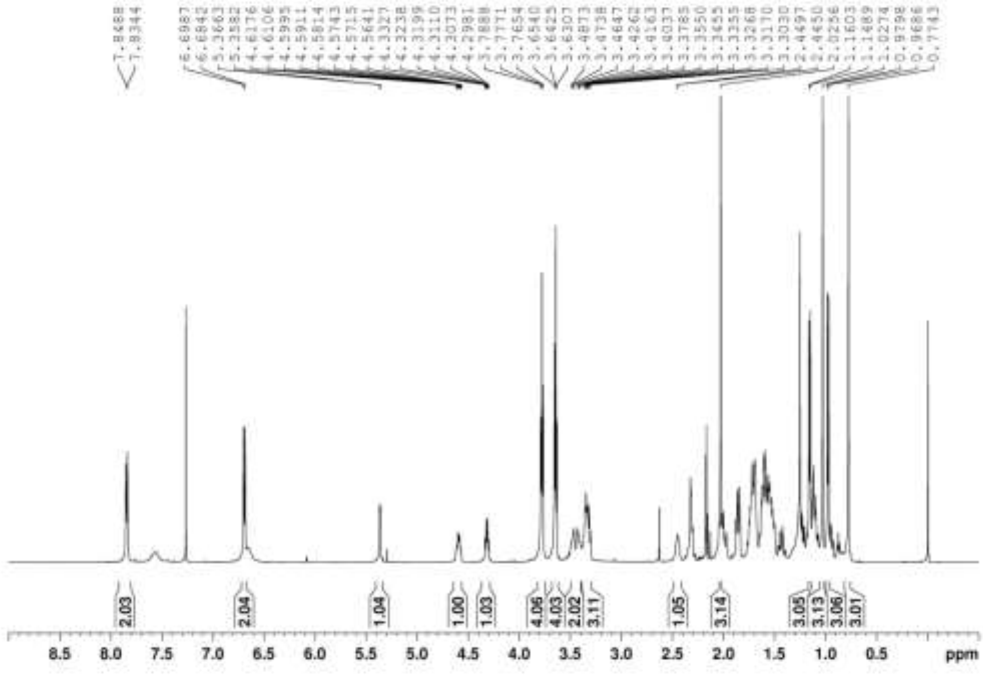

Figure S58. ^{1}H NMR of compound **14b**

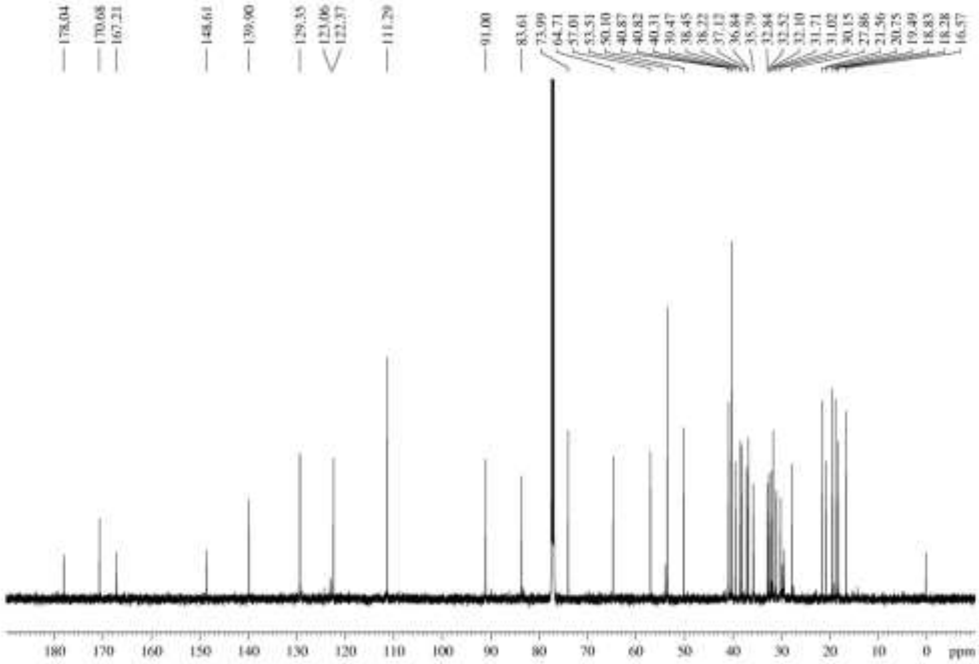

Figure S59. ^{13}C NMR of compound **14b**

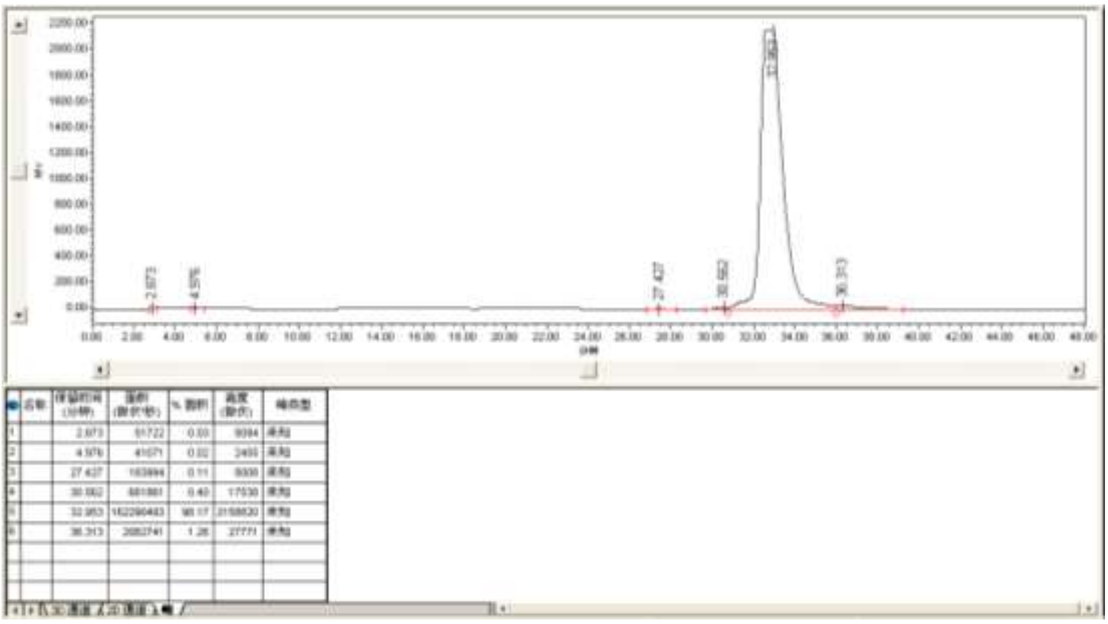

Figure S60. HPLC spectrum of compound **14b**

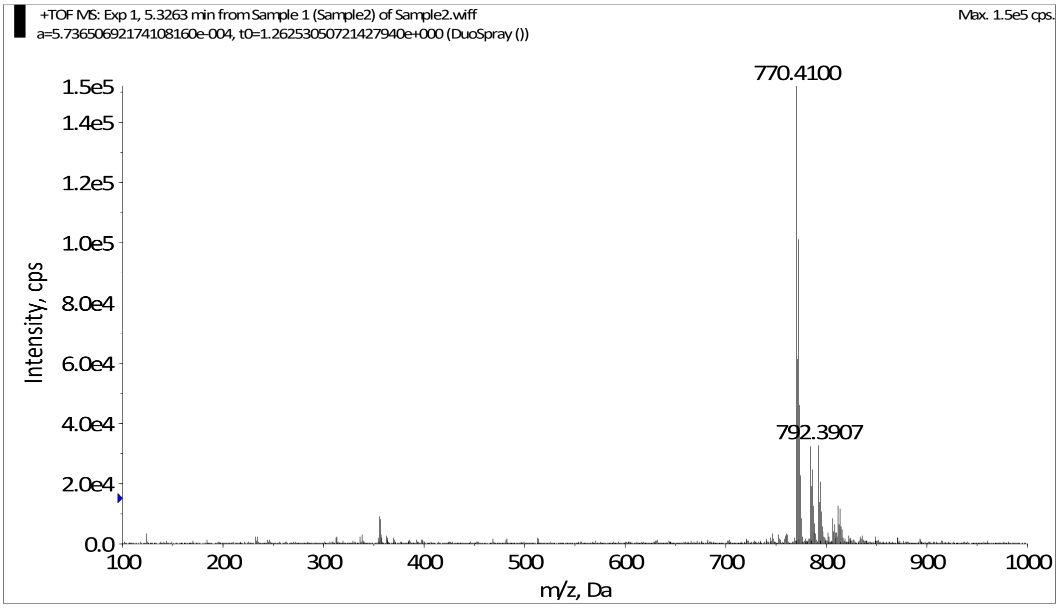

Figure S61. HR-MS of compound **14c**

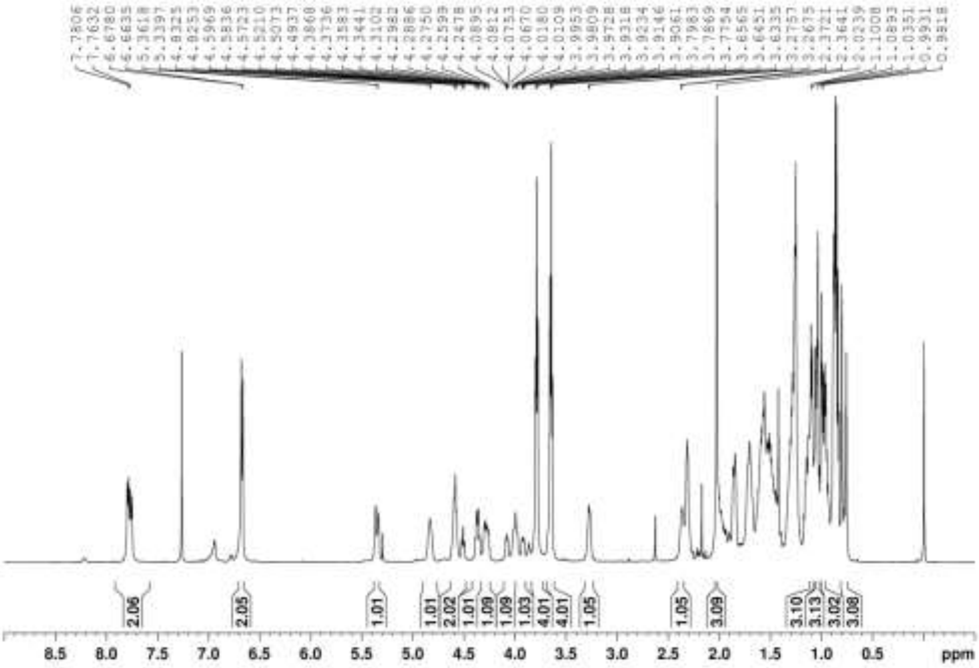

Figure S62. ^{1}H NMR of compound **14c**

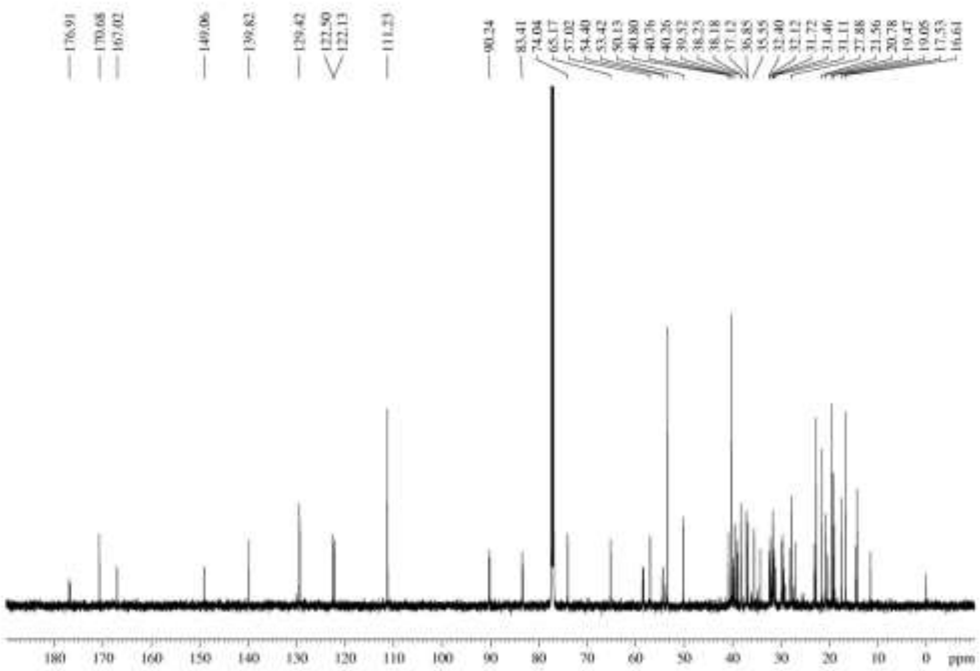

Figure S63. ^{13}C NMR of compound **14c**

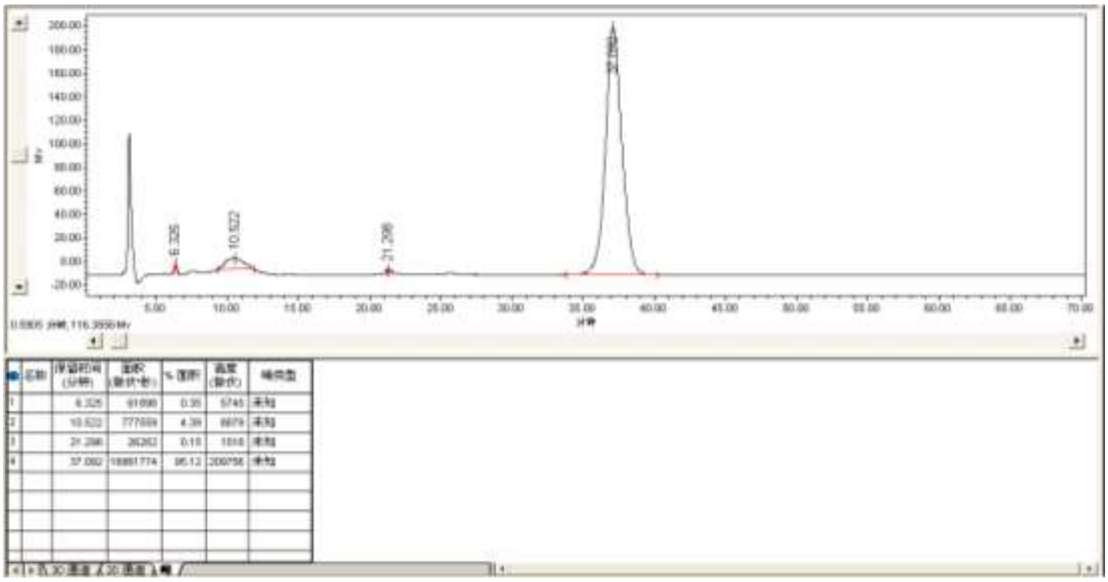

Figure S64. HPLC spectrum of compound **14c**

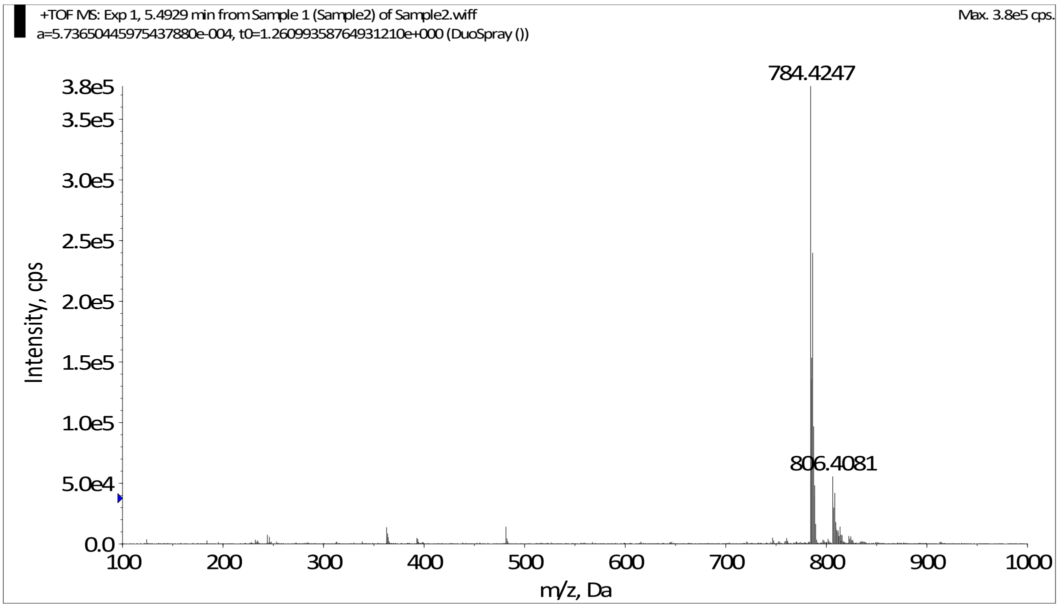

Figure S65. HR-MS of compound **14d**

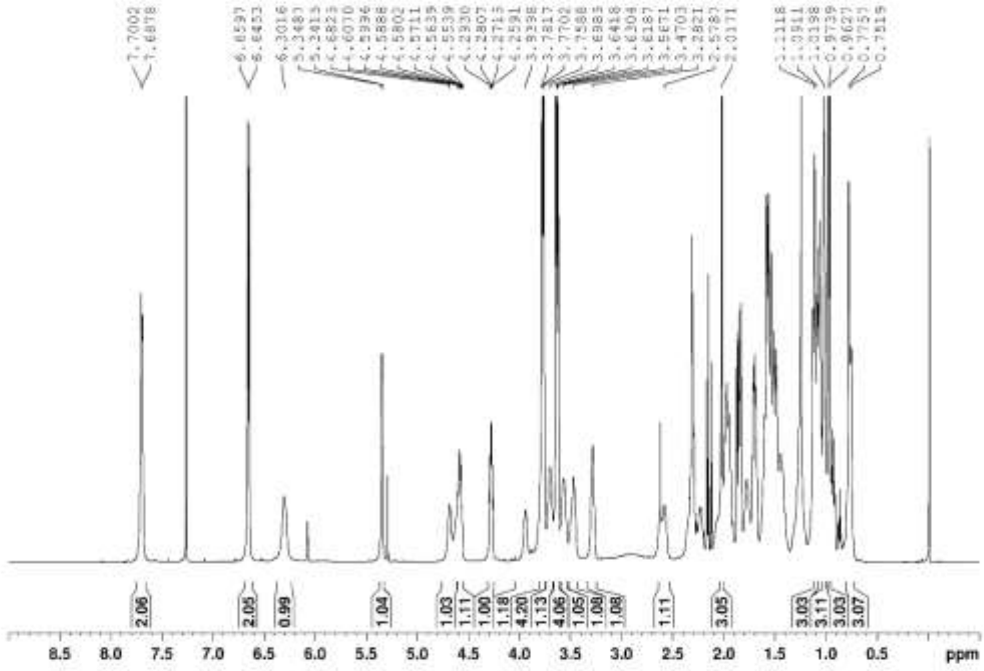

Figure S66. ^{1}H NMR of compound **14d**

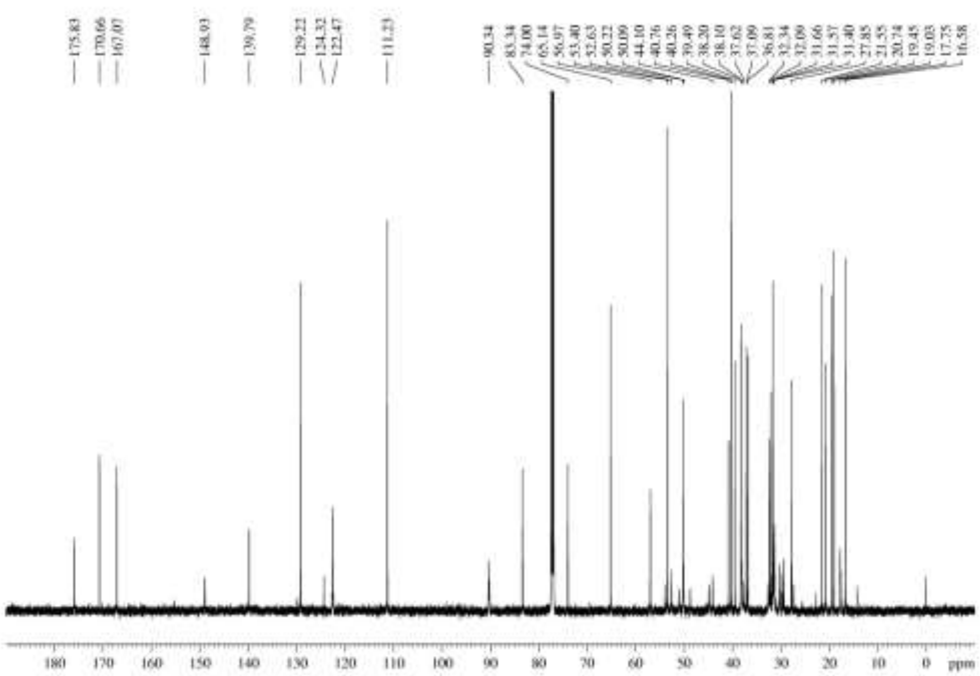

Figure S67. ^{13}C NMR of compound **14d**

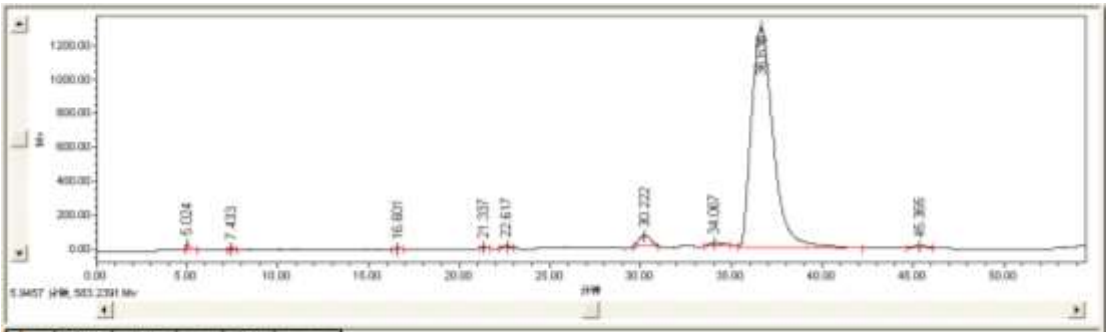

	名称	保留时间 (分钟)	面积 (微伏*秒)	% 面积	高度 (微伏)	峰类型
1		5.024	533182	0.47	30613	未知
2		7.433	135502	0.12	8453	未知
3		16.601	142984	0.13	6179	未知
4		21.337	154596	0.14	6760	未知
5		22.617	270068	0.24	9584	未知
6		30.222	2807909	2.48	63302	未知
7		34.087	799107	0.71	15708	未知
8		36.636	107790686	95.21	1297764	未知
9		45.365	582219	0.51	10806	未知

Figure S68. HPLC spectrum of compound **14d**

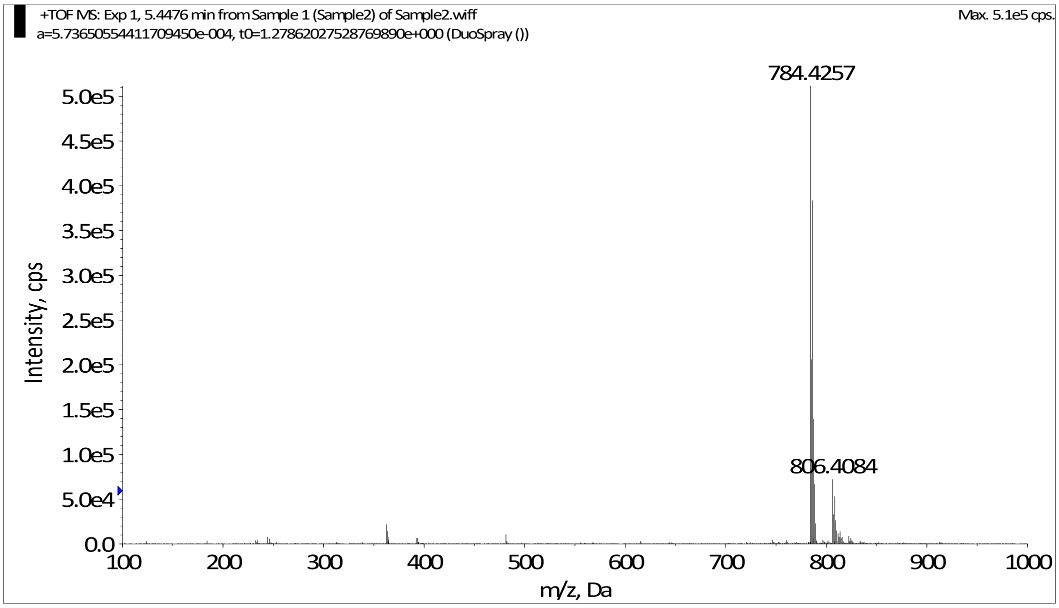

Figure S69. HR-MS of compound **14e**

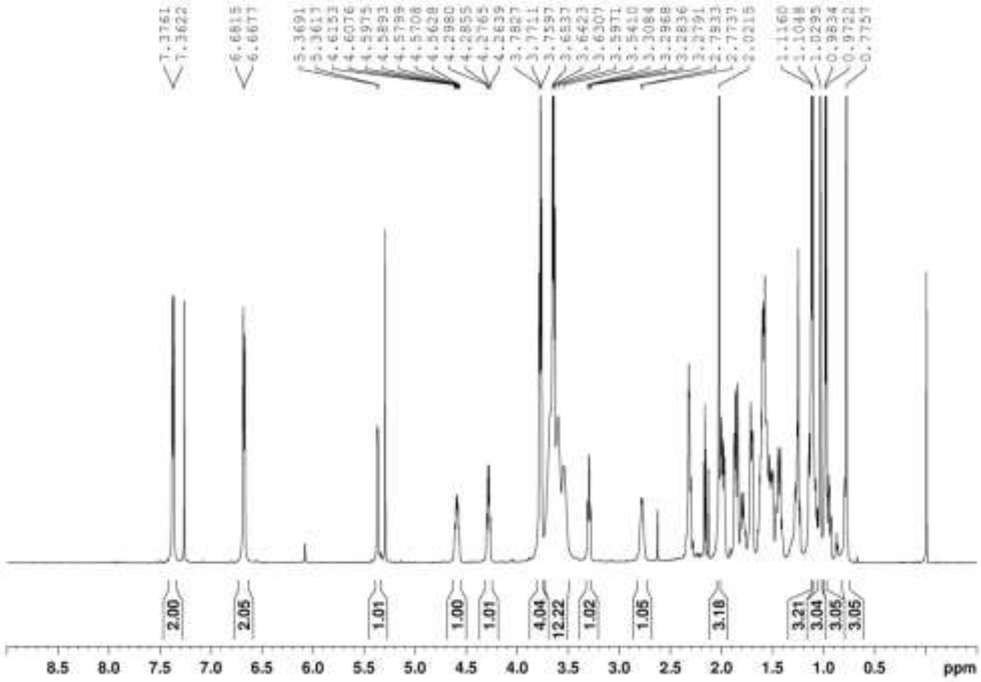

Figure S70. ^{1}H NMR of compound **14e**

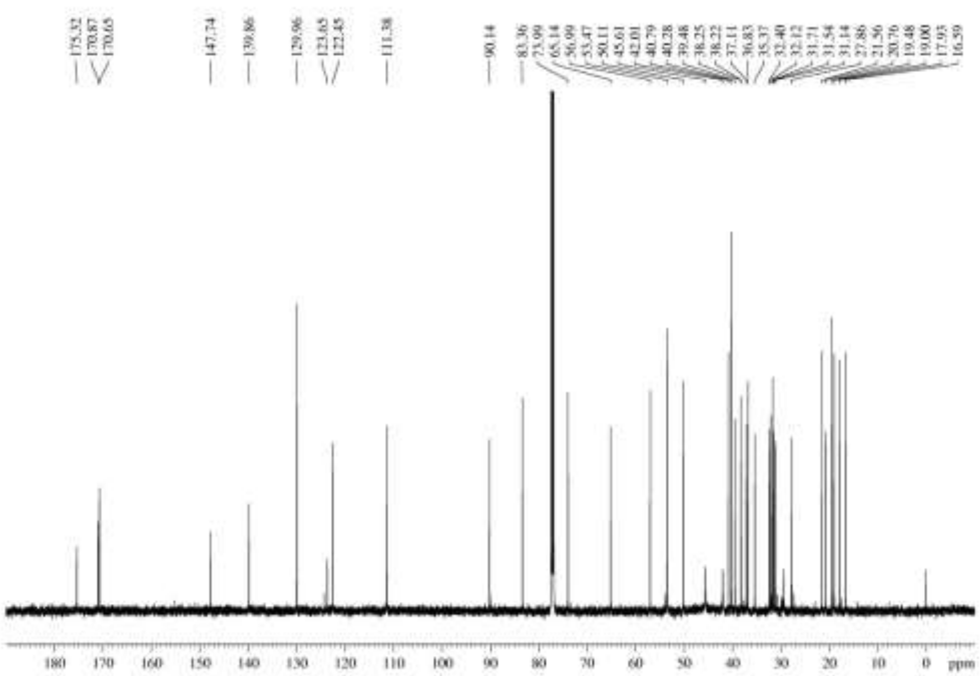

Figure S71. ^{13}C NMR of compound **14e**

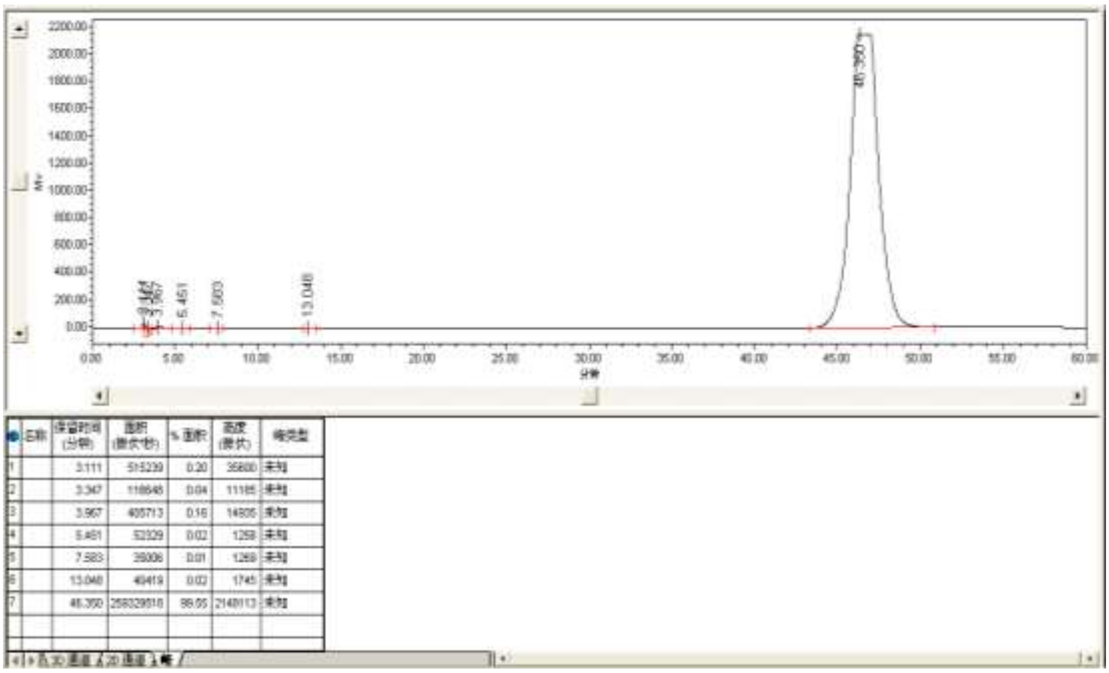

Figure S72. HPLC spectrum of compound **14e**

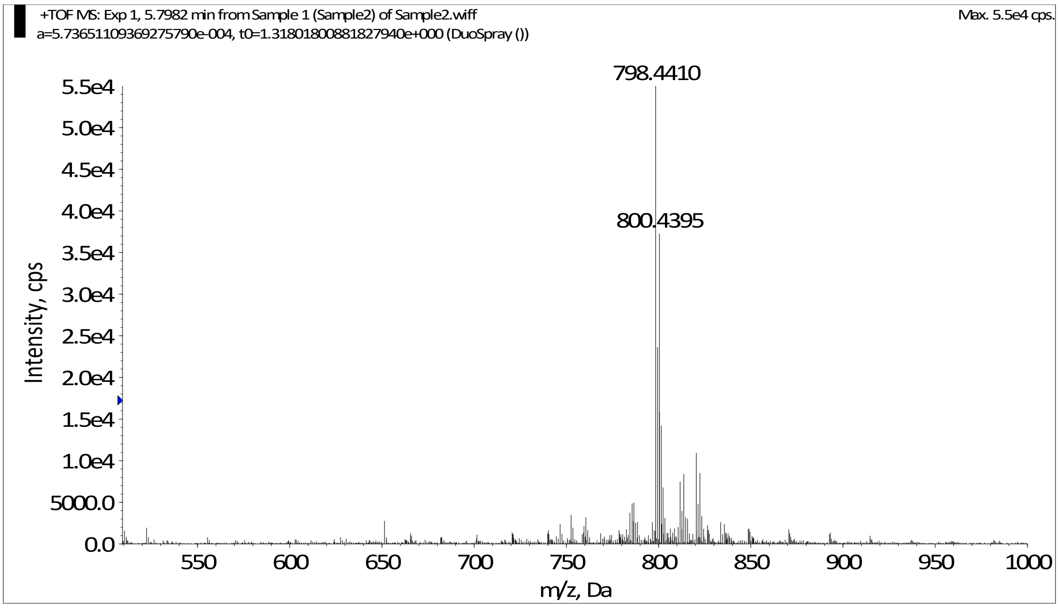

Figure S73. HR-MS of compound **14f**

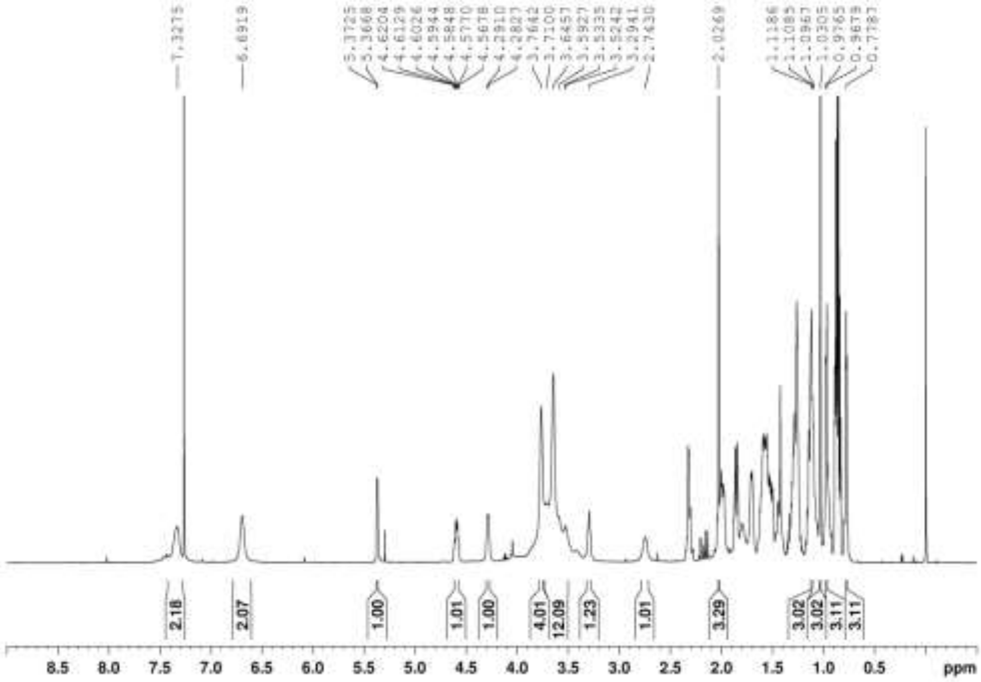

Figure S74. ^{1}H NMR of compound **14f**

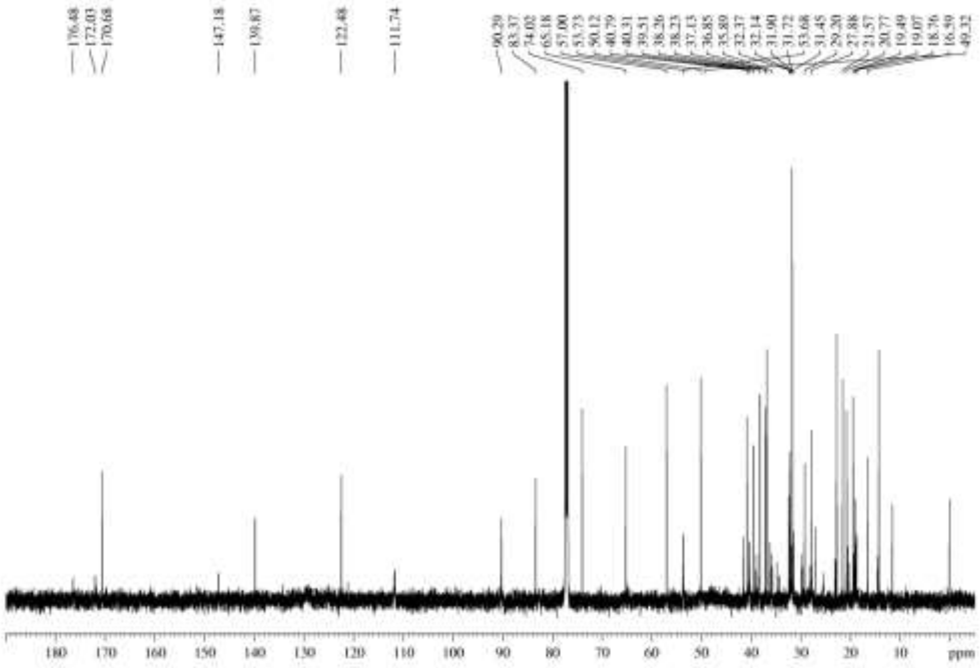

Figure S75. ^{13}C NMR of compound **14f**

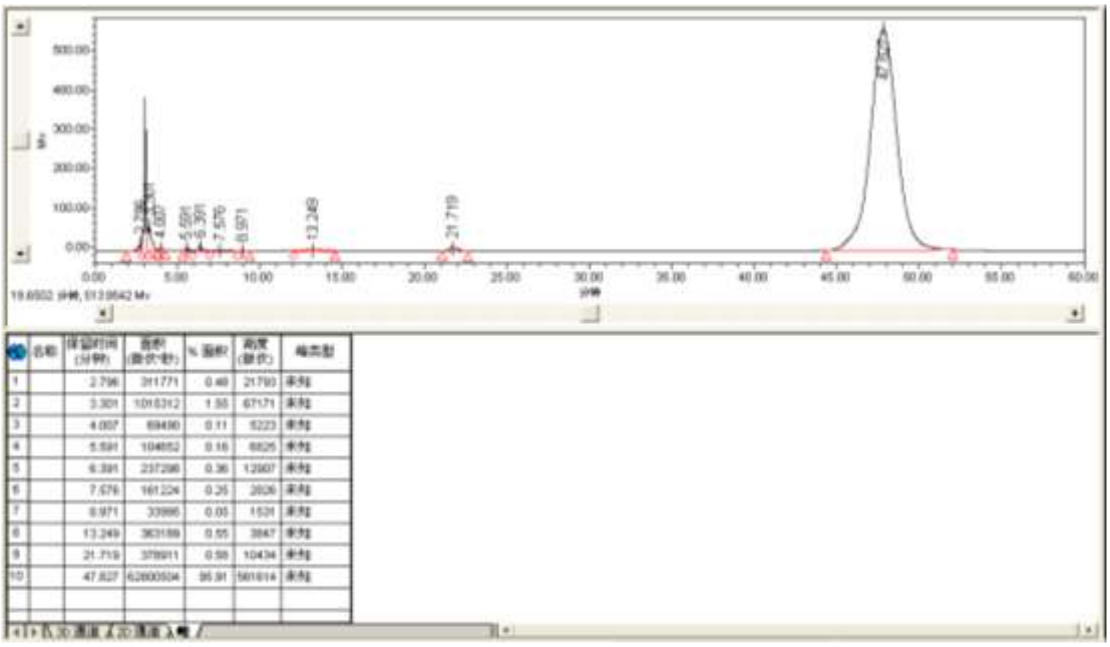

Figure S76. HPLC spectrum of compound **14f**

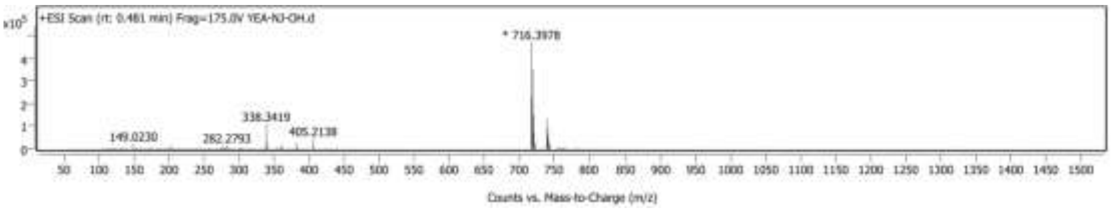

Figure S77. HR-MS of compound **15a**

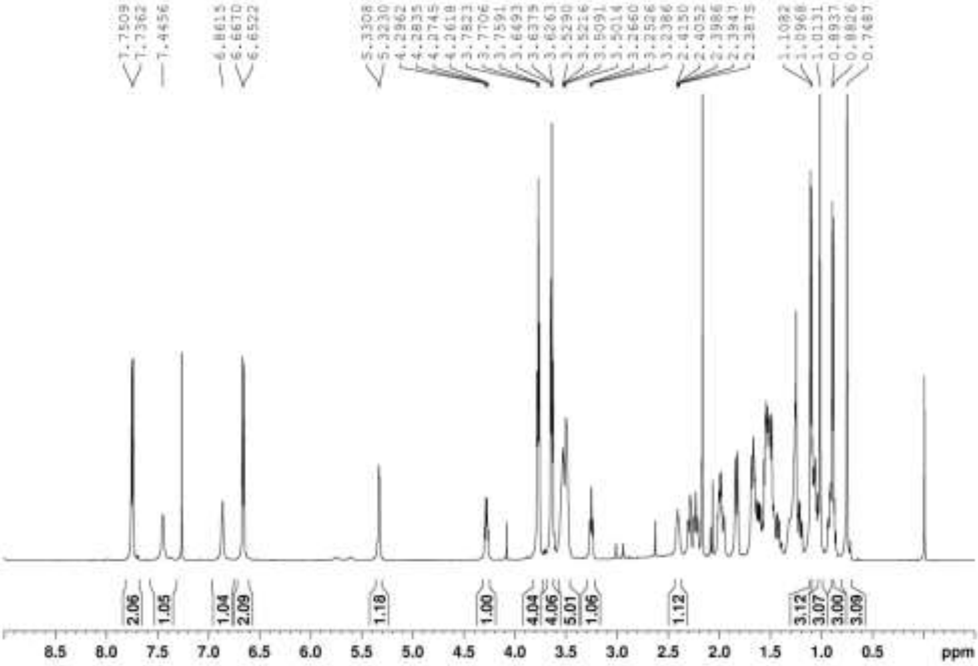

Figure S78. ^{1}H NMR of compound **15a**

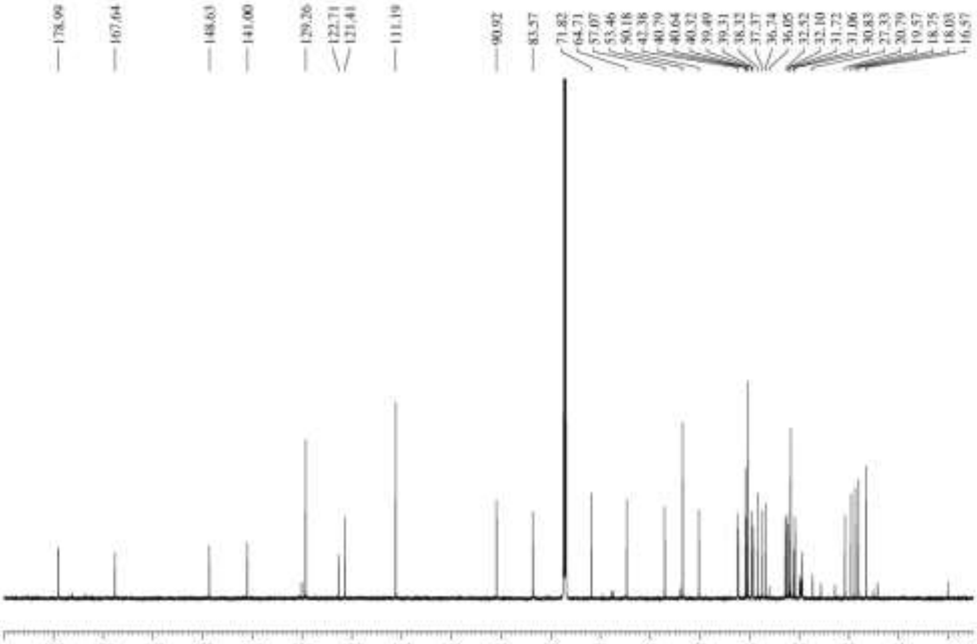

Figure S79. ^{13}C NMR of compound **15a**

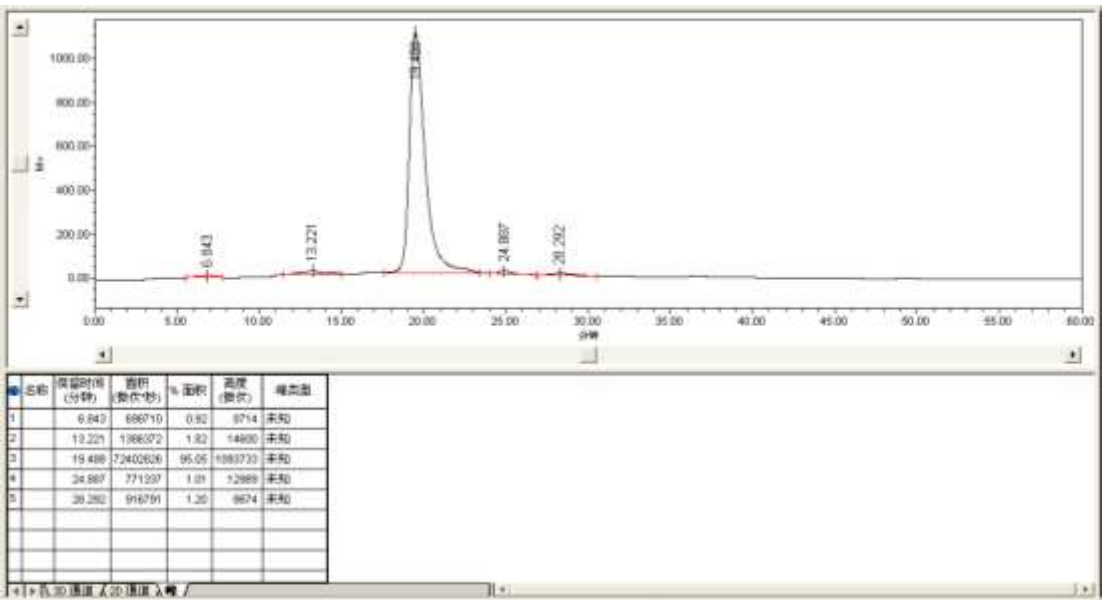

Figure S80. HPLC spectrum of compound **15a**

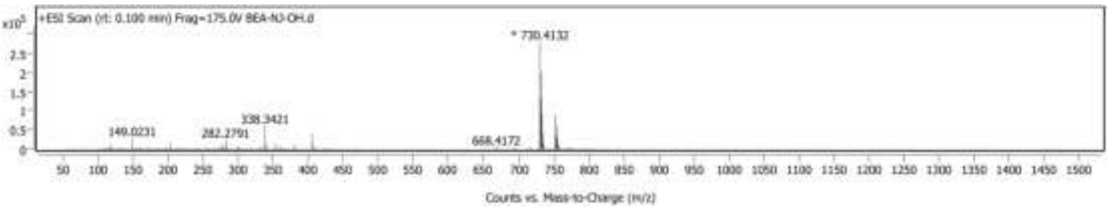

Figure S81. HR-MS of compound **15b**

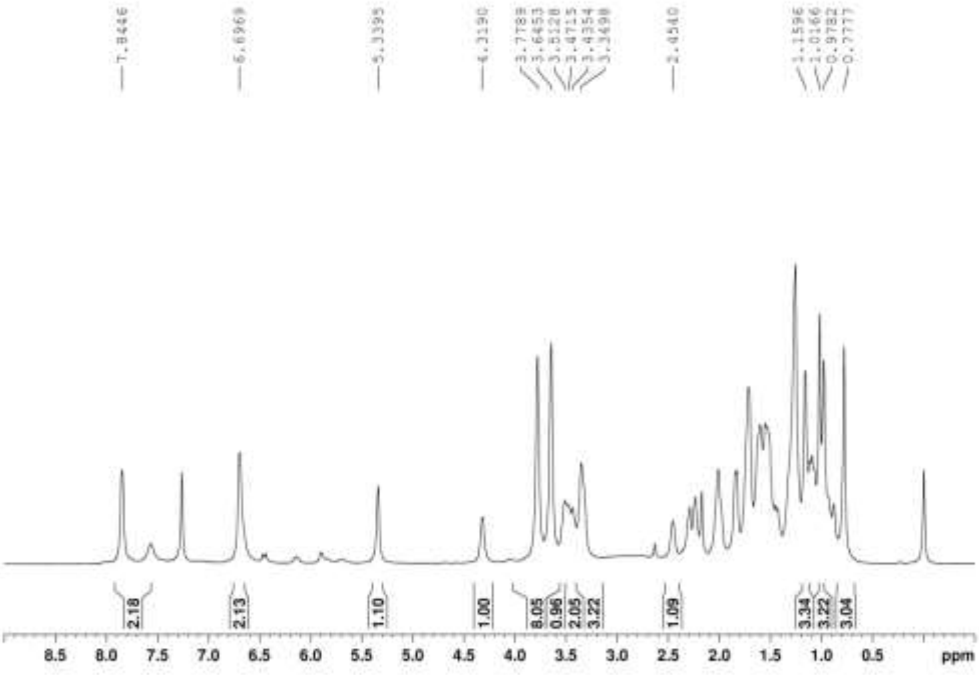

Figure S82. ^{1}H NMR of compound **15b**

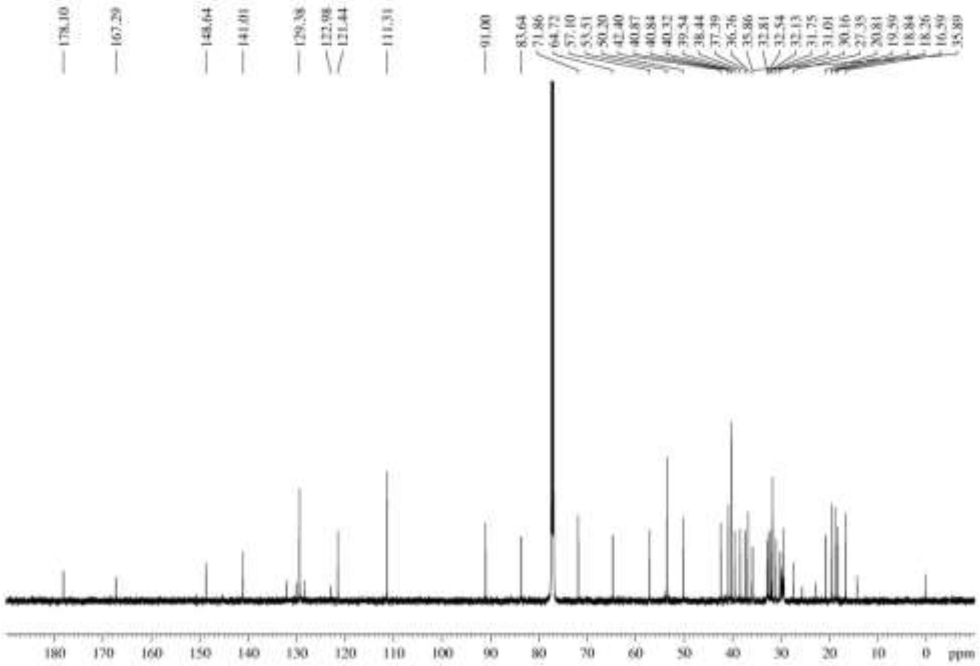

Figure S83. ^{13}C NMR of compound **15b**

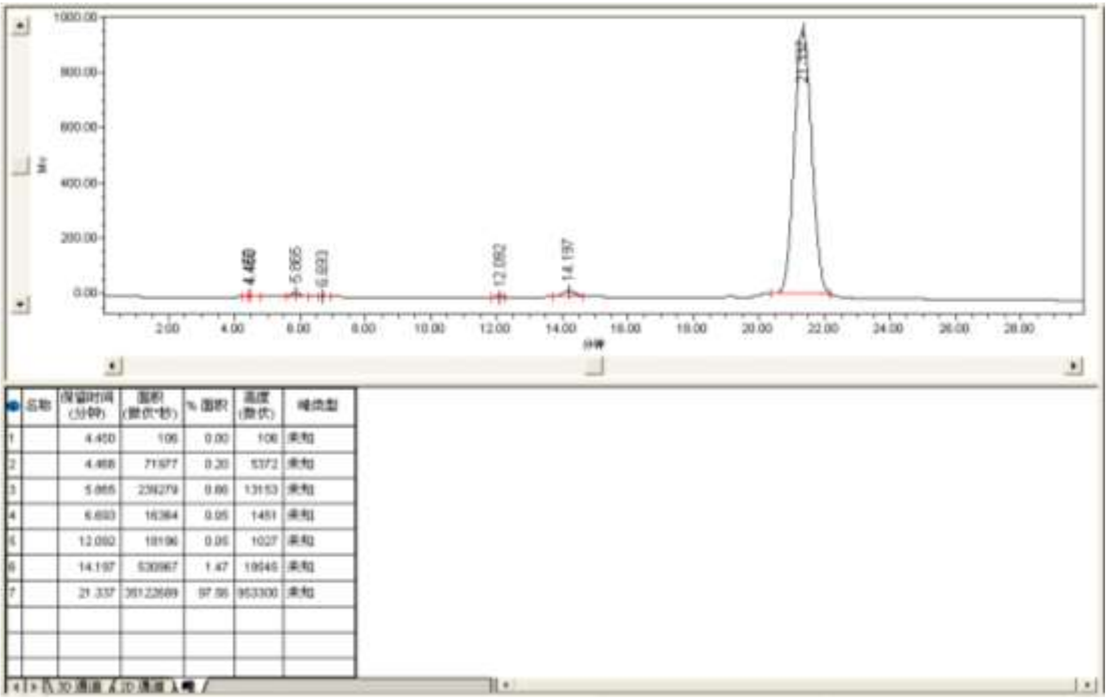

Figure S84. HPLC spectrum of compound **15b**

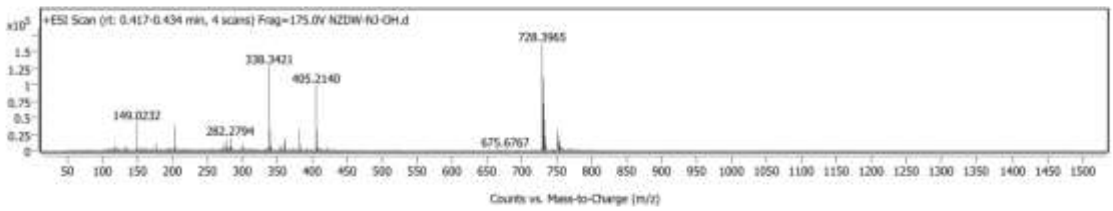

Figure S85. HR-MS of compound **15c**

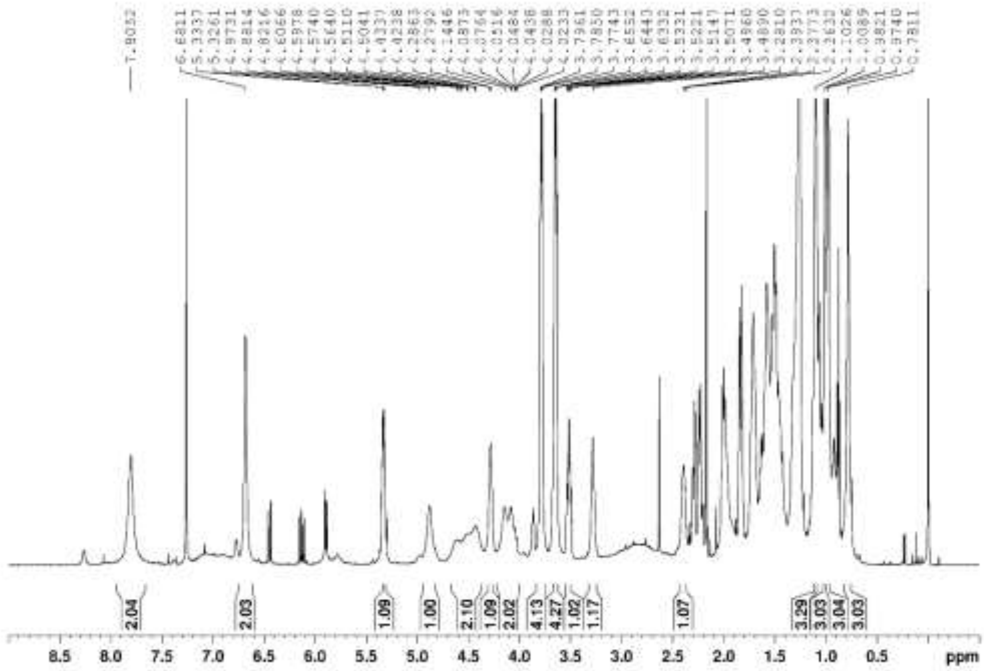

Figure S86. ^{1}H NMR of compound **15c**

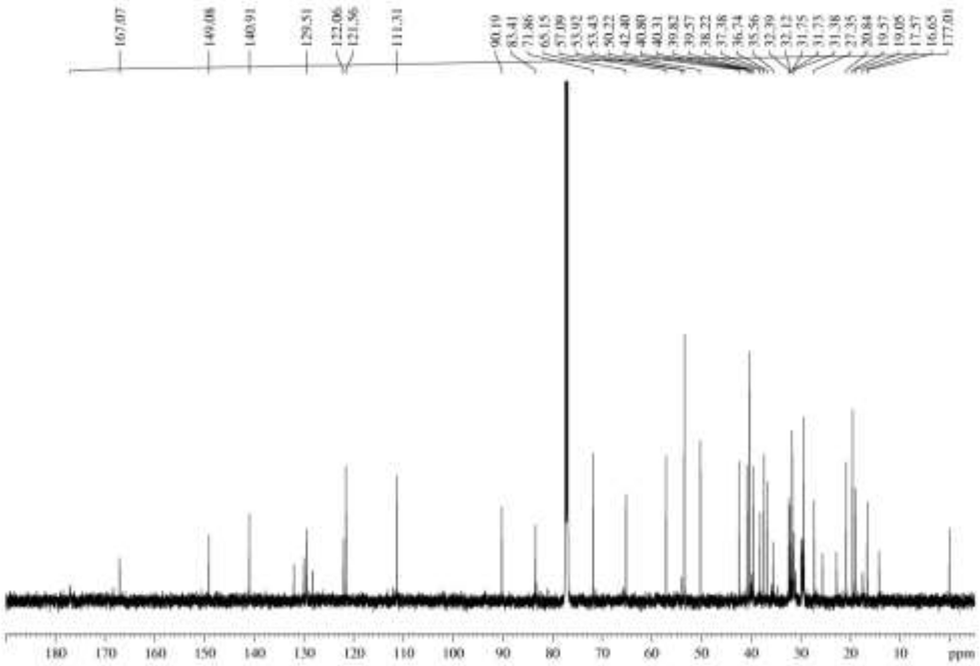

Figure S87. ^{13}C NMR of compound **15c**

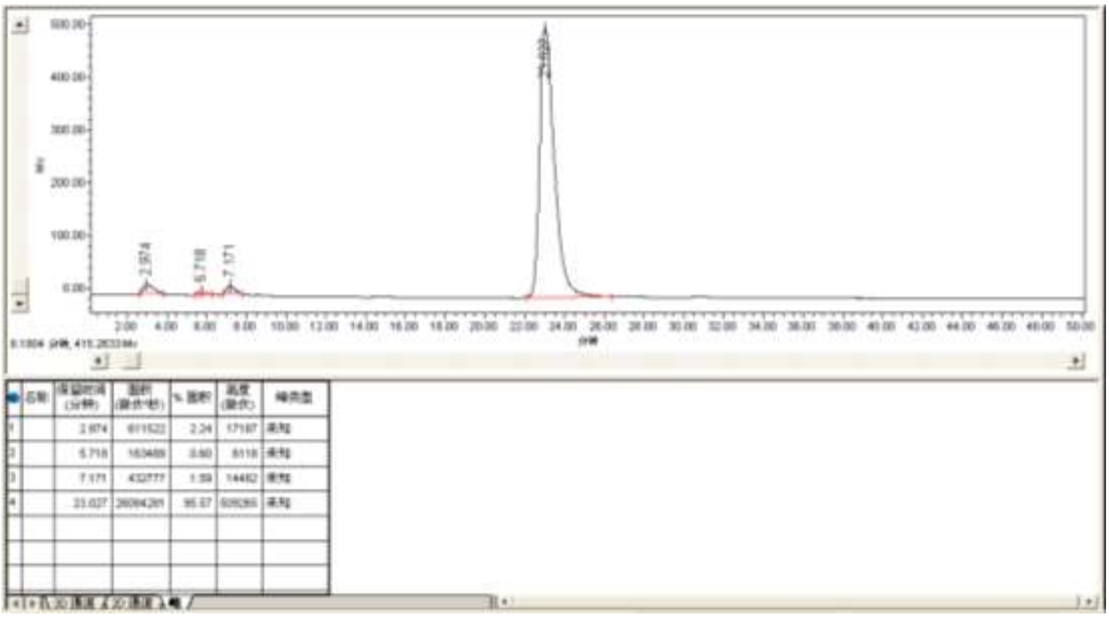

Figure S88. HPLC spectrum of compound **15c**

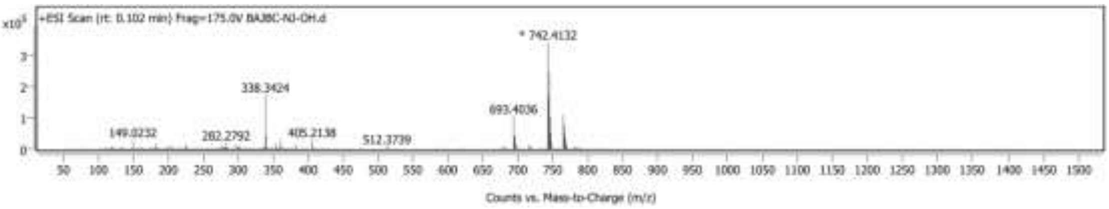

Figure S89. HR-MS of compound **15d**

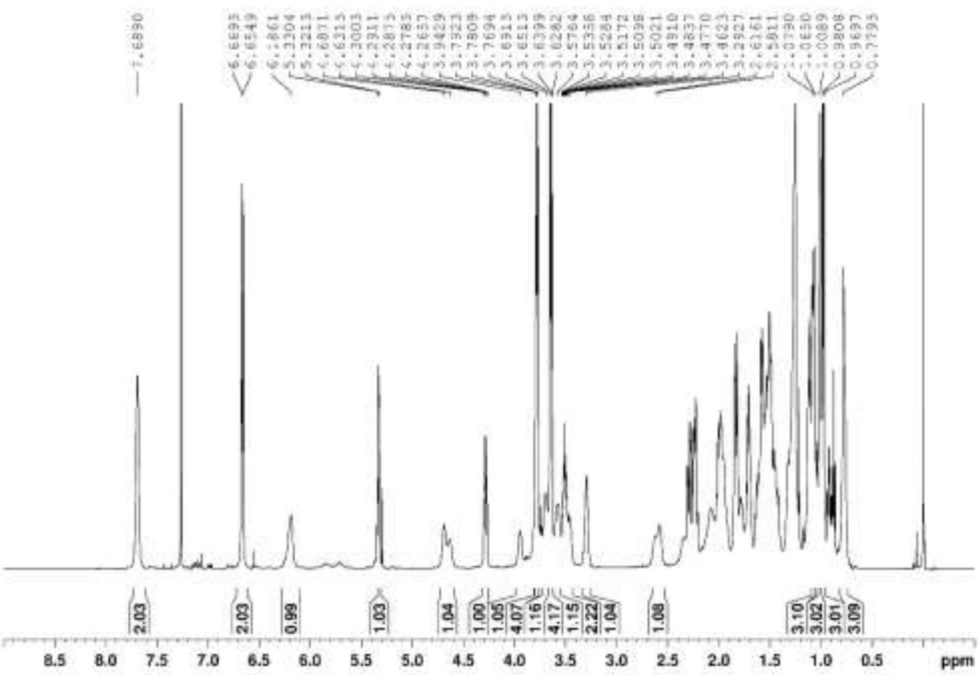

Figure S90. ^{1}H NMR of compound **15d**

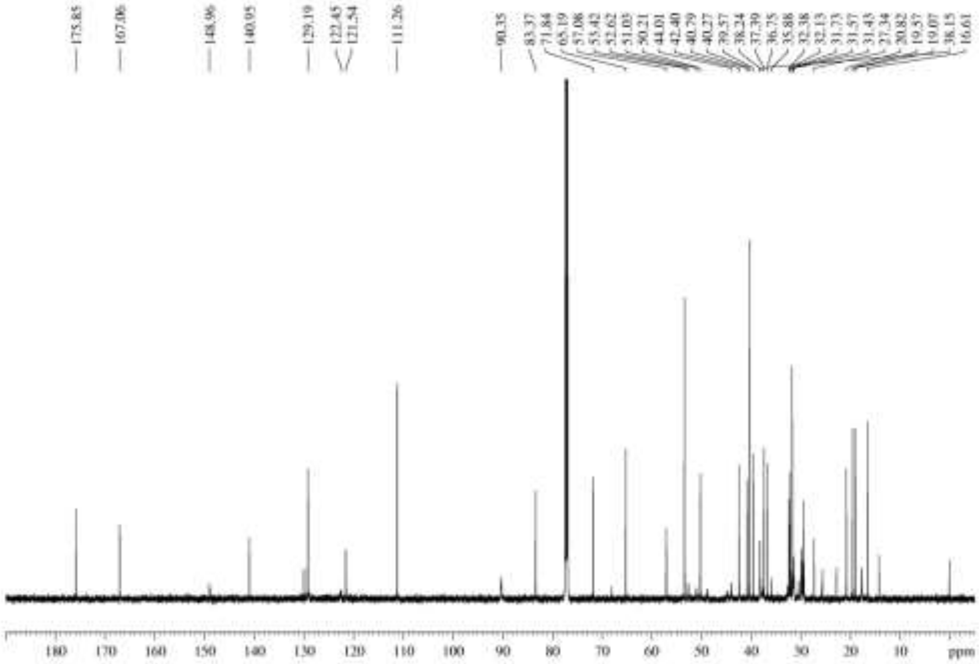

Figure S91. ^{13}C NMR of compound **15d**

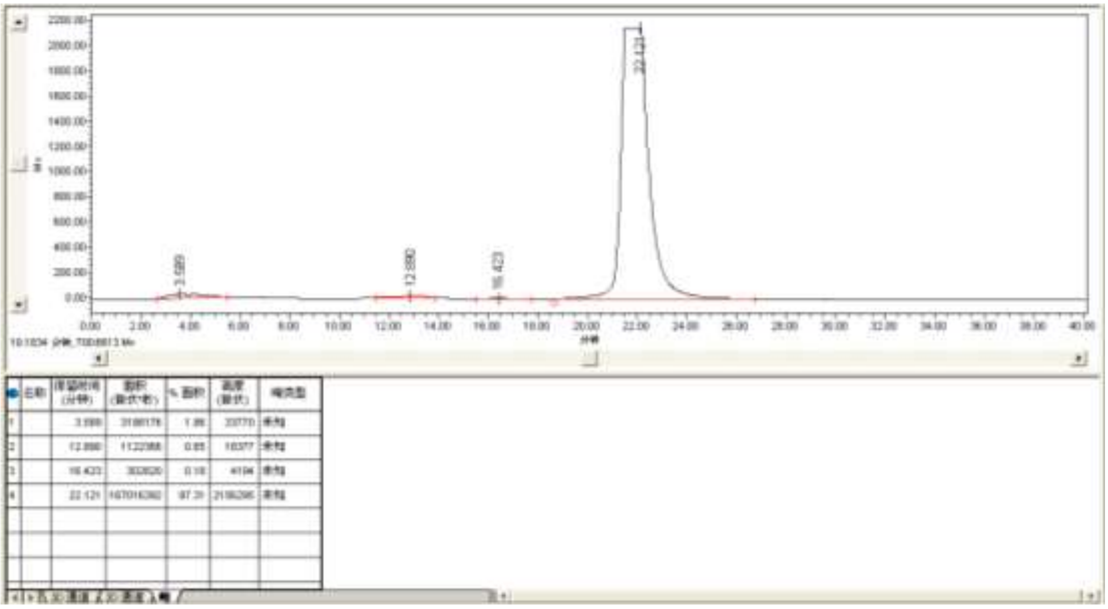

Figure S92. HPLC spectrum of compound **15d**

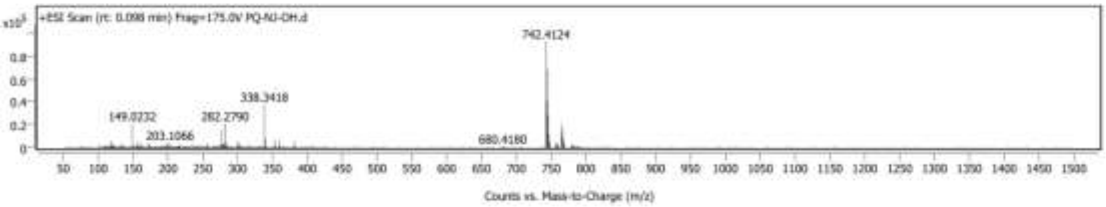

Figure S93. HR-MS of compound **15e**

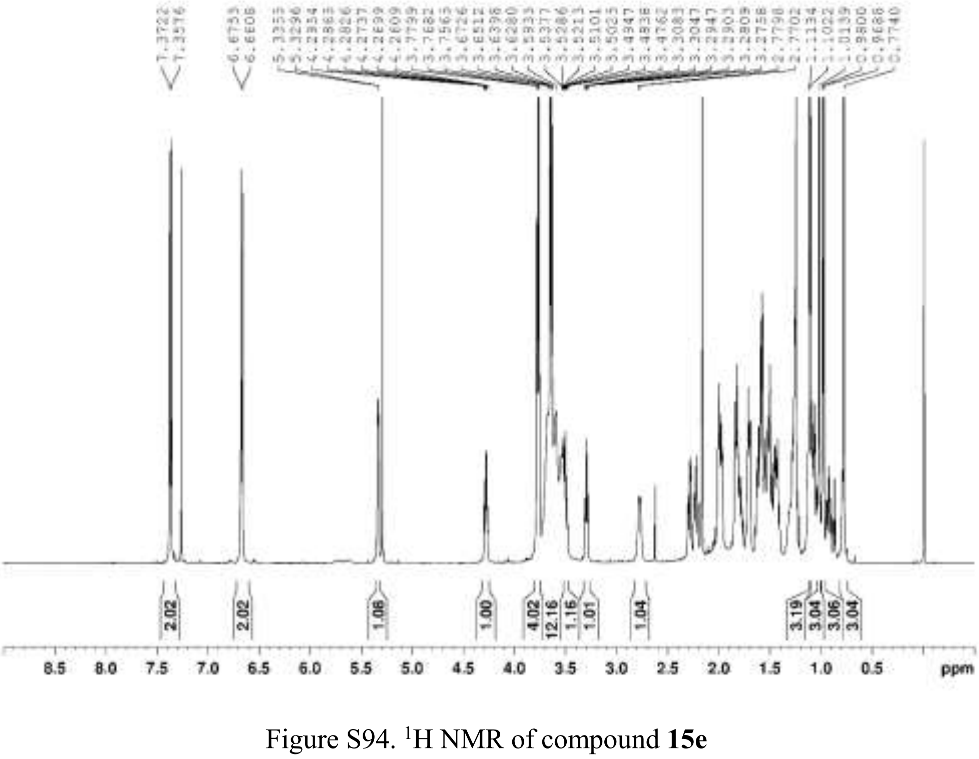

Figure S94. ^{1}H NMR of compound **15e**

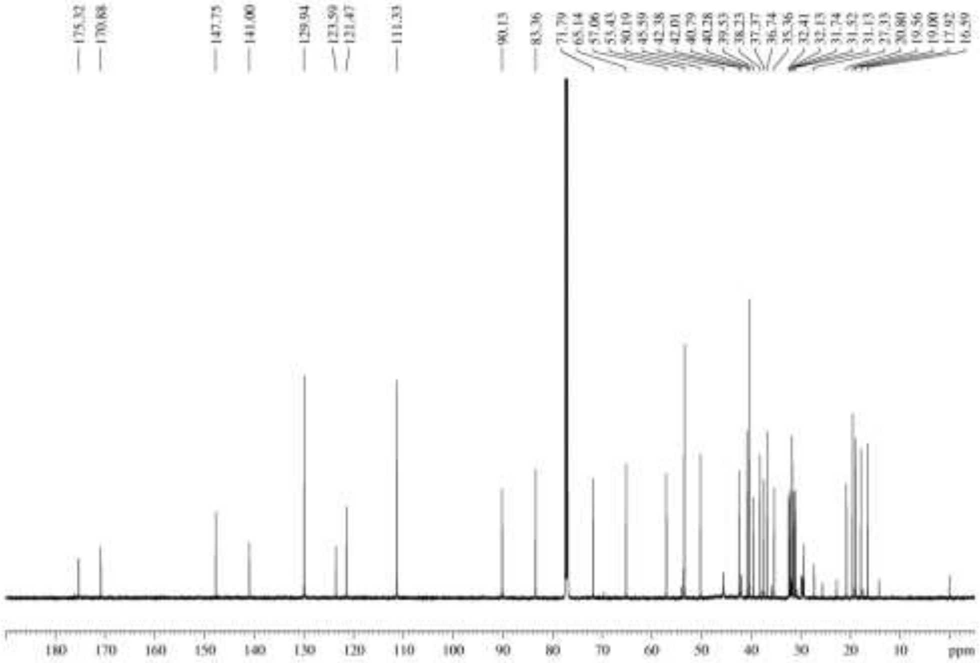

Figure S95. ^{13}C NMR of compound **15e**

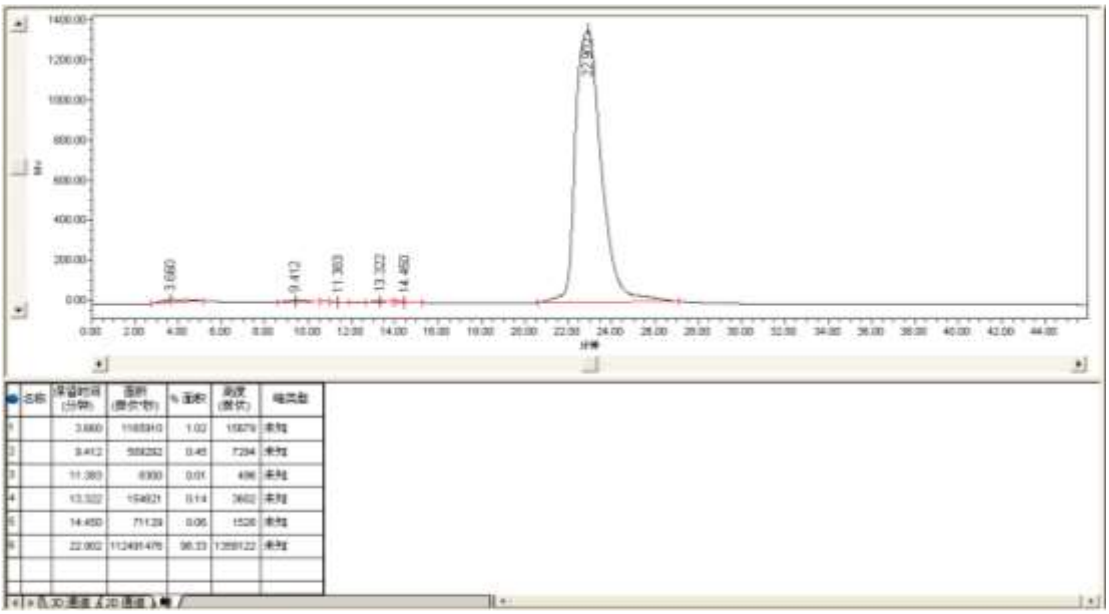

Figure S96. HPLC spectrum of compound **15e**

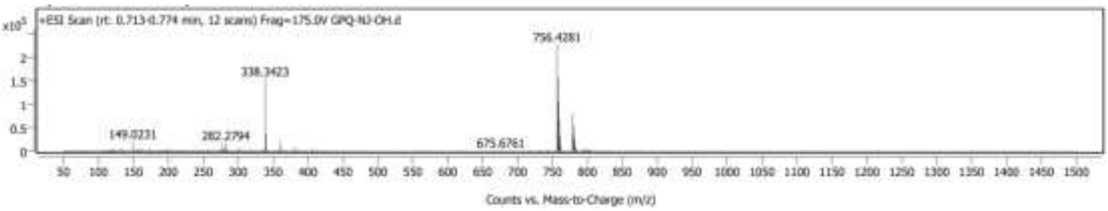

Figure S97. HR-MS of compound **15f**

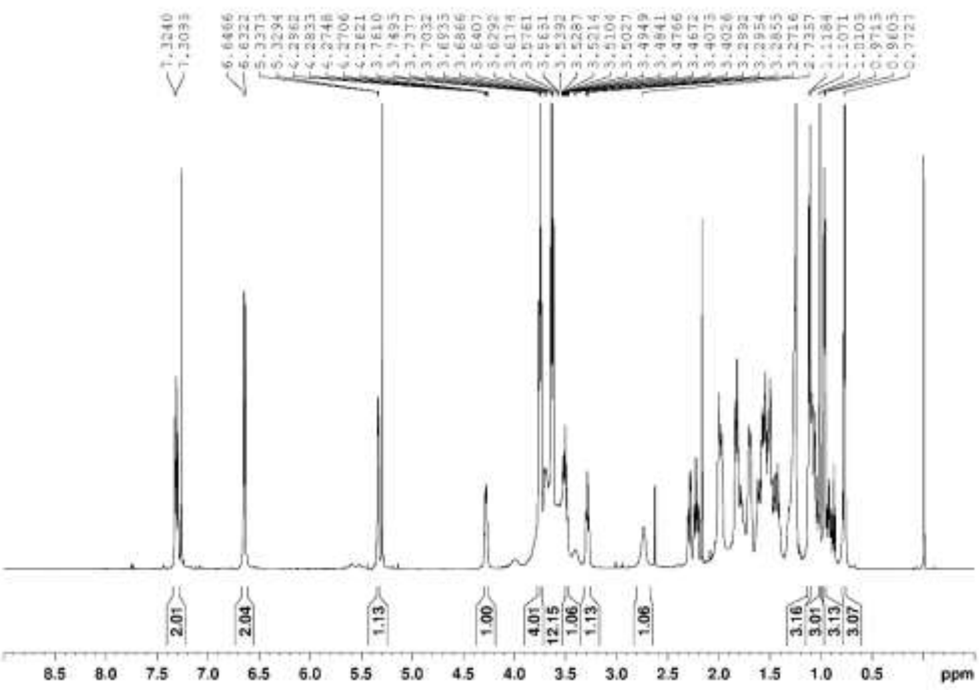

Figure S98. ^{1}H NMR of compound **15f**

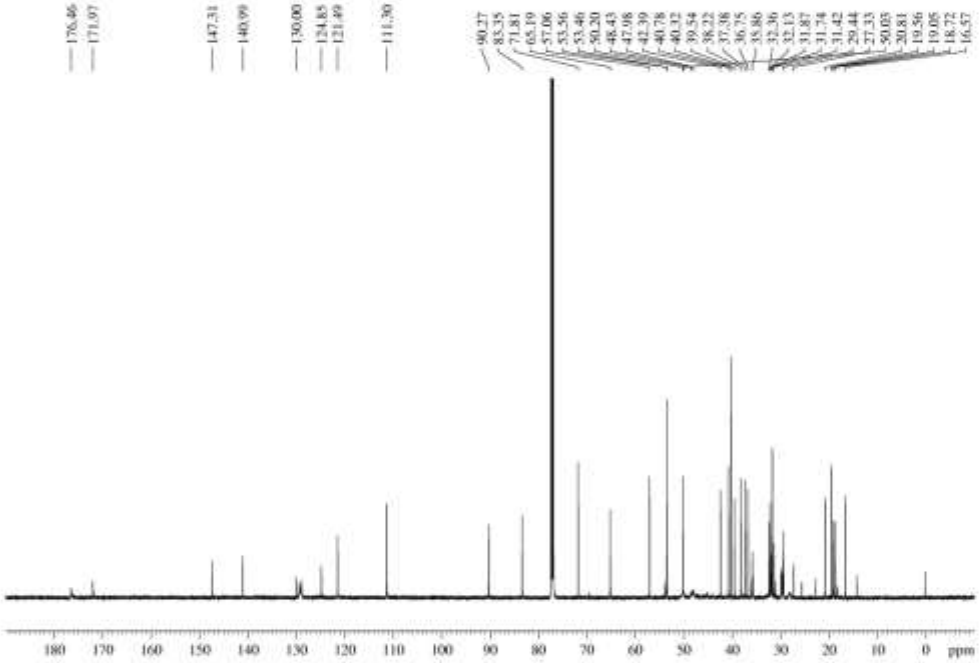

Figure S99. ^{13}C NMR of compound **15f**

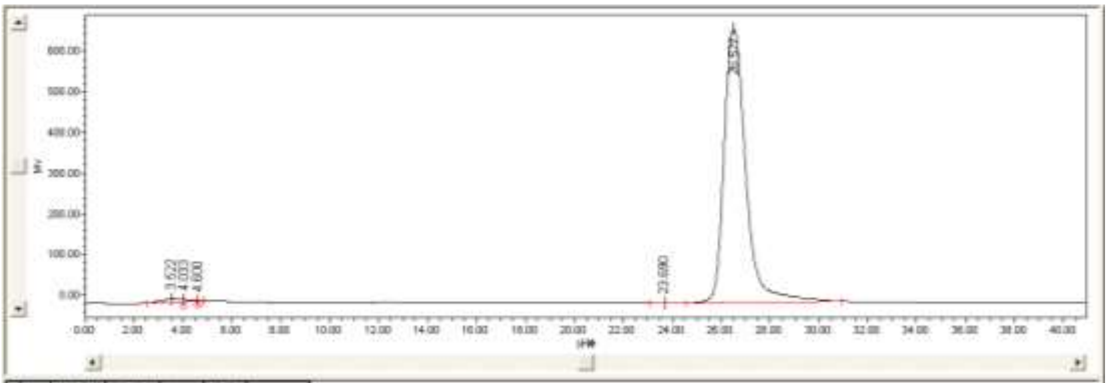

	名称	保留时间 (分钟)	面积 (微伏*秒)	% 面积	高度 (微伏)	峰类型
1		3.522	456214	1.09	8861	未知
2		4.033	83108	0.20	5003	未知
3		4.600	9622	0.02	1467	未知
4		23.690	51681	0.12	1062	未知
5		26.527	41341071	98.57	671581	未知

Figure S100. HPLC spectrum of compound **15f**
